# Different Patterns of Akt and ERK Feedback Activation in Response to Rapamycin, Active-Site mTOR Inhibitors and Metformin in Pancreatic Cancer Cells

**DOI:** 10.1371/journal.pone.0057289

**Published:** 2013-02-21

**Authors:** Heloisa P. Soares, Yang Ni, Krisztina Kisfalvi, James Sinnett-Smith, Enrique Rozengurt

**Affiliations:** 1 Division of Digestive Diseases, Department of Medicine; CURE: Digestive Diseases Research Center David Geffen School of Medicine and Molecular Biology Institute, University of California at Los Angeles, Los Angeles, California, United States of America; 2 Division of Hematology-Oncology, Department of Medicine, University of California at Los Angeles, Los Angeles, California, United States of America; 3 Department of Thoracic Surgery, Provincial Hospital Affiliated to Shandong University, Jinan, Shandong, China; Duke University Medical Center, United States of America

## Abstract

The mTOR pathway is aberrantly stimulated in many cancer cells, including pancreatic ductal adenocarcinoma (PDAC), and thus it is a potential target for therapy. However, the mTORC1/S6K axis also mediates negative feedback loops that attenuate signaling via insulin/IGF receptor and other tyrosine kinase receptors. Suppression of these feed-back loops unleashes over-activation of upstream pathways that potentially counterbalance the antiproliferative effects of mTOR inhibitors. Here, we demonstrate that treatment of PANC-1 or MiaPaCa-2 pancreatic cancer cells with either rapamycin or active-site mTOR inhibitors suppressed S6K and S6 phosphorylation induced by insulin and the GPCR agonist neurotensin. Rapamycin caused a striking increase in Akt phosphorylation at Ser^473^ while the active-site inhibitors of mTOR (KU63794 and PP242) completely abrogated Akt phosphorylation at this site. Conversely, active-site inhibitors of mTOR cause a marked increase in ERK activation whereas rapamycin did not have any stimulatory effect on ERK activation. The results imply that first and second generation of mTOR inhibitors promote over-activation of different pro-oncogenic pathways in PDAC cells, suggesting that suppression of feed-back loops should be a major consideration in the use of these inhibitors for PDAC therapy. In contrast, metformin abolished mTORC1 activation without over-stimulating Akt phosphorylation on Ser^473^ and prevented mitogen-stimulated ERK activation in PDAC cells. Metformin induced a more pronounced inhibition of proliferation than either KU63794 or rapamycin while, the active-site mTOR inhibitor was more effective than rapamycin. Thus, the effects of metformin on Akt and ERK activation are strikingly different from allosteric or active-site mTOR inhibitors in PDAC cells, though all these agents potently inhibited the mTORC1/S6K axis.

## Introduction

The mammalian target of rapamycin (mTOR) is a highly evolutionarily conserved protein kinase that plays a key role in the integration of growth factor, nutrient and energy status of the cells [1]. mTOR functions as a catalytic subunit in two distinct multiprotein complexes, mTOR complex 1 (mTORC1) and mTORC2. mTORC1, characterized by the regulatory subunit Raptor, controls at least two regulators of protein synthesis, the 40S ribosomal protein subunit S6 kinase (S6K) and the eukaryotic translation initiation factor 4E (eIF4E)-binding protein 1, referred as 4E-BP1 [1], [2]. The heterodimer of the tumor suppressor TSC2 (tuberin) and TSC1 (hamartin) represses mTORC1 signaling by acting as the GTPase-activator protein for the small G protein Rheb (Ras homolog enriched in brain), a potent activator of mTORC1 signaling in its GTP-bound state [3], [4]. Phosphorylation of TSC2 by Akt and/or ERK/p90RSK suppresses its GTPase activating activity towards Rheb, leading to mTORC1 activation [5]. mTORC1 is acutely and allosterically inhibited by rapamycin through binding to FKBP12. mTORC2, characterized by Rictor, is not inhibited by short-term treatment with this agent and phosphorylates several AGC protein kinases, including Akt at Ser^473^ [6], [7]. The mTORC1 pathway plays a key role in insulin/IGF receptor signaling [8], [9] and is aberrantly activated in many cancers, including pancreatic ductal adenocarcinoma (PDAC), one of the most lethal human diseases. Accordingly, PDAC cells express insulin and IGF-1 receptors and over-express IRS-1 and IRS-2 [10]–[12] and PDAC (but not normal) tissue display activated (phosphorylated) IGF-1R [13]. Gene variations in the IGF-1 signaling system have been associated to worse survival in patients with PDAC [14]. Inactivation of p53, as seen during the progression of 50–70% of PDAC, up-regulates the insulin/IGF-1/mTORC1 pathway [15]. Crosstalk between insulin/IGF-1 receptors and G protein-coupled receptor (GPCR) signaling systems potently stimulate mTORC1, DNA synthesis and cell proliferation in a panel of PDAC cells [16]–[20]. mTORC1 signaling plays a pivotal role in the proliferation and survival of PDAC cells [21] and is activated in pancreatic cancer tissues [20], [22]–[24]. Consequently, mTORC1 has emerged as an attractive therapeutic target in PDAC and other common malignancies.

In addition to growth-promoting signaling, mTORC1/S6K also mediates negative feedback loops that restrain signaling through insulin/IGF receptor and other tyrosine kinase receptors via phosphorylation and transcriptional repression of IRS-1 [25]–[30] and phosphorylation of Grb10 [31], [32]. Consequently, suppression of mTORC1 activity by rapamycin prevents inhibitory IRS-1 phosphorylations and degradation, thereby augmenting PI3K/Akt activation in several cancer cell types [30], [33]–[35]. These studies imply that the potential anti-cancer activity of rapamycin (or analogs) can be counterbalanced by release of feedback inhibition of PI3K/Akt activation [25], [30], [33]–[35]. Furthermore, rapamycin incompletely inhibits 4E-BP-1 phosphorylation [36]–[40]. Accordingly, the clinical antitumor activity of rapamycin and its analogs (rapalogs) has been rather limited in many types of cancer [41], [42], including PDAC [43], [44]. In an effort to target the mTOR pathway more effectively, novel inhibitors of mTOR that act at the catalytic active site (active-site mTOR inhibitors) have been identified, including PP242 [37], Torin [45], KU63794 [38] and its analogue AZD8055 [46]. These compounds inhibit 4E-BP-1 phosphorylation at rapamycin-resistant sites (e.g. Thr^37/46^) and block Akt phosphorylation at Ser^473^ through inhibition of mTORC2. However, active-site mTOR inhibitors also eliminate feedback loops that restrain PI3K activation [25] and consequently, their therapeutic effectiveness can also be diminished by activation of upstream pathways that oppose their anti-proliferative effects.

mTORC1 is also negatively regulated by metformin, the most widely used drug in the treatment of type 2 diabetes mellitus (T2DM). Metformin is emerging as a potential novel agent in cancer chemoprevention. Recent epidemiological studies linked administration of metformin to reduced incidence, recurrence and mortality of a variety of cancers in T2DM patients [20], [47]–[56], including PDAC [54], [56]. At the cellular level, metformin indirectly stimulates AMP–activated protein kinase (AMPK) activation [57], though other mechanisms of action have been proposed at very high concentrations of this biguanide. AMPK inhibits mTORC1 activation through stimulation of TSC2 function [58]–[60], leading to accumulation of Rheb-GDP (the inactive form) and by direct phosphorylation of Raptor, which disrupts its association with mTOR, leading to dissociation of the mTORC1 complex [61]. The precise consequence of suppression of negative feedback loops mediated by the mTORC1/S6K axis in response to metformin remains poorly defined and, in particular, it is not known whether rapamycin, active-site mTOR inhibitors and metformin lead to over-activation of similar upstream pathways in PDAC cells.

Here, we demonstrate that treatment of PANC-1 or MiaPaCa-2 pancreatic cancer cells with either rapamycin or active-site mTOR inhibitors suppressed S6K and S6 phosphorylation induced by insulin, a combination of insulin and the GPCR agonist neurotensin or serum. Rapamycin caused a striking augmentation of Akt phosphorylation at Ser^473^ while the active-site mTOR inhibitors KU63794 and PP242 completely abrogated Akt phosphorylation at this site. A salient feature of the results presented here is that active-site inhibitors of mTOR, in contrast to rapamycin, cause a marked increase in ERK activation in PDAC cells. The results imply that first and second generation mTOR inhibitors promote over-activation of different pro-oncogenic pathways in PDAC cells, namely Akt and ERK. Metformin also abolished mTORC1 activation but without over-stimulating Akt phosphorylation on Ser^473^. Furthermore, metformin prevented ERK activation in response to cross-talking agonists in PDAC cells. Our results demonstrate that the effects of metformin on Akt and ERK activation are strikingly different from those elicited by allosteric or active-site mTOR inhibitors, though all these agents potently inhibited the mTORC1/S6K axis.

## Materials and Methods

### Cell culture

The human pancreatic cancer cell lines PANC-1 and MiaPaCa-2 were obtained from the American Type Culture Collection (ATCC, Manassas, VA). These cell lines harbor activating mutations in the *KRAS* oncogene. Cells were grown in Dulbecco's modified Eagle Medium (DMEM) with 2 mM glutamine, 1 mM Na-pyruvate, 100 units/mL penicillin, and 100 µg/mL streptomycin and 10% fetal bovine serum (FBS) at 37°C in a humidified atmosphere containing 10% CO_2_.

### Western blot analysis

Confluent cultures of PANC-1 or MIA PaCa-2 cells grown on 3 cm dishes were washed and then incubated for 24 hr with DMEM containing 5 mM glucose and 1% FBS. The cells were washed twice with DMEM containing 5 mM glucose and incubated in serum-free medium for 4 h and then treated as described in individual experiments. The cultures were then directly lysed in 2× SDS-PAGE sample buffer [200 mM Tris-HCl (pH 6.8), 2 mM EDTA, 0.1 M Na_3_VO_4_, 6% SDS, 10% glycerol, and 4% 2-mercaptoethanol], followed by SDS-PAGE on 10% gels and transfer to Immobilon-P membranes (Millipore, Billerica, MA). Western blots were then performed on membranes incubated overnight with the specified antibodies in phosphate-buffered saline (PBS) containing 0.1% Tween-20. The immunoreactive bands were detected with ECL (enhanced chemiluminescence) reagents (GE Healthcare Bio-Sciences Corp, Piscataway, NJ). The antibodies used detected the phosphorylated state of S6K at Thr^389^, S6 at Ser^235/236^, 4E-BP1 at Thr^37/46^ and Thr^70^, Akt at Ser^473^ and Thr^308^ and ERK1/2 at Thr^202^ and Tyr^204^ or the total levels of these proteins.

#### Anchorage-dependent cell proliferation

PANC-1 cells (10^5^) were plated on 35 mm tissue culture dishes in DMEM containing 10% FBS. After 24 h of incubation at 37°C, groups of cultures were incubated with neurotensin and insulin with or without metformin in DMEM containing 0.25% FBS or Rapamycin, KU63794 or Metformin in DMEM containing 2.5% FBS. The cultures were then incubated for 4 d, and the total cell count was determined from a minimum of six wells per condition using a Coulter counter, after cell clumps were disaggregated by passing the cell suspension 10 times through a 19-gauge, and subsequently, a 21-gauge needle.

### Materials

DMEM was obtained from Invitrogen (Carlsbad, CA). Neurotensin and insulin were obtained from Sigma Chemical (St. Louis, MO). Rapamycin, KU63794 and PP242 were from R&D Systems, Inc. Minneapolis. All antibodies were purchased from Cell Signaling Technology (Danvers, MA). Horseradish peroxidase–conjugated anti-rabbit IgG and anti-mouse IgG were from GE Healthcare Bio-Sciences Corp (Piscataway, NJ). All other reagents were of the highest grade available.

## Results

### Stimulation of p70S6K and S6 phosphorylation in response to insulin and neurotensin in PDAC cells is completely abolished by rapamycin or KU63794

**Figure 1:**
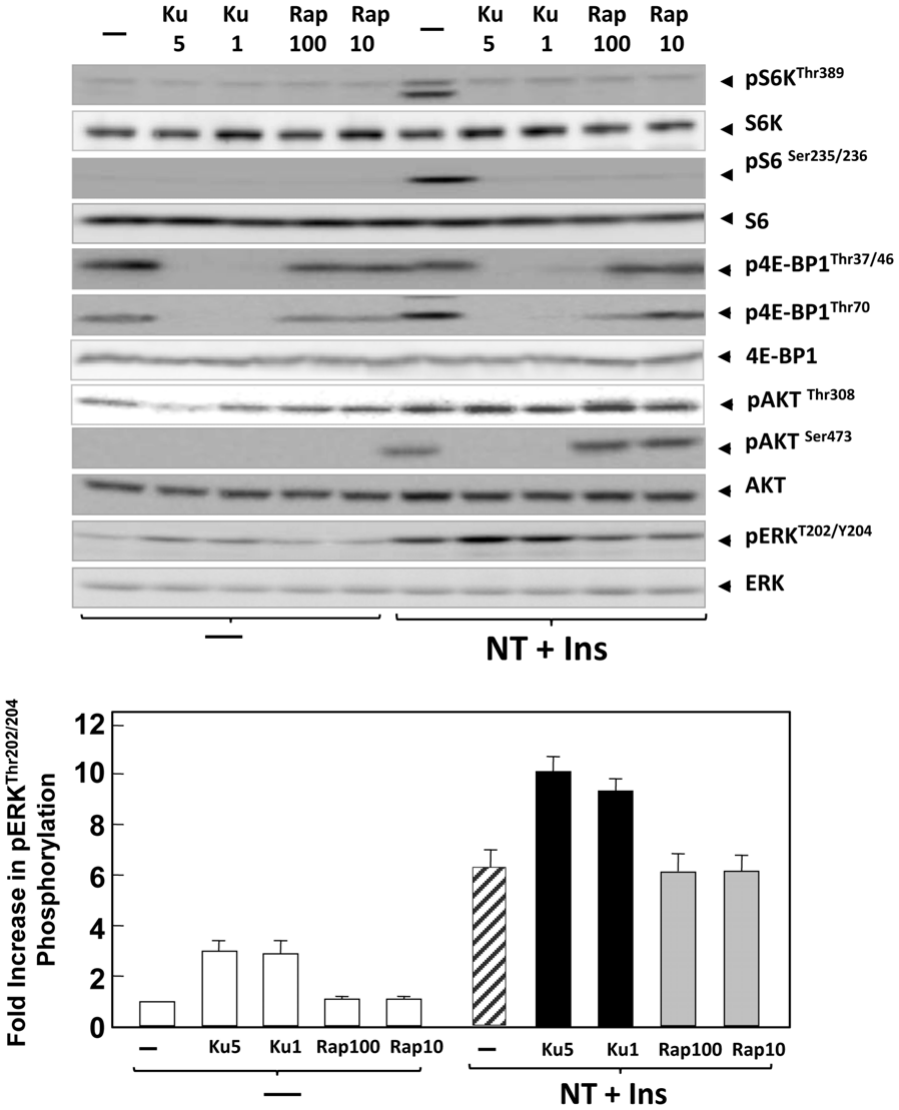
Differential feedback activation of Akt and ERK phosphorylation by rapamycin and KU63794 in PANC-1 cells. Cultures of PANC-1 cells were incubated in the absence (−) or in the presence of KU63794 (Ku) at 1 µM or 5 µM or rapamycin (Rap) at 10 or 100 nM for 2 h in DMEM containing 5 mM glucose, as indicated. Then, the cells were stimulated for 2 h with 5 nM neurotensin (NT) and 10 ng/ml insulin (Ins) and lysed with 2×SDS–PAGE sample buffer. The samples were analyzed by SDS-PAGE and immunoblotting with antibodies that detect the phosphorylated state of S6K at Thr^389^, S6 at Ser^235/236^, 4E-BP1 at Thr^37/46^ and Thr^70^, Akt at Ser^473^ and Thr^308^ and ERK at Thr^202^ and Tyr^204^. Immunoblotting with antibodies that recognize total S6K, S6, 4E-BP1, Akt and ERK was used to verify that the cell treatments did not change the total level of these proteins and confirm equal gel loading. Fold increase in ERK phosphorylation was quantified using Multi Gauge V3.0 and plotted as bars. Similar results were obtained in 3 independent experiments.

**Figure 2:**
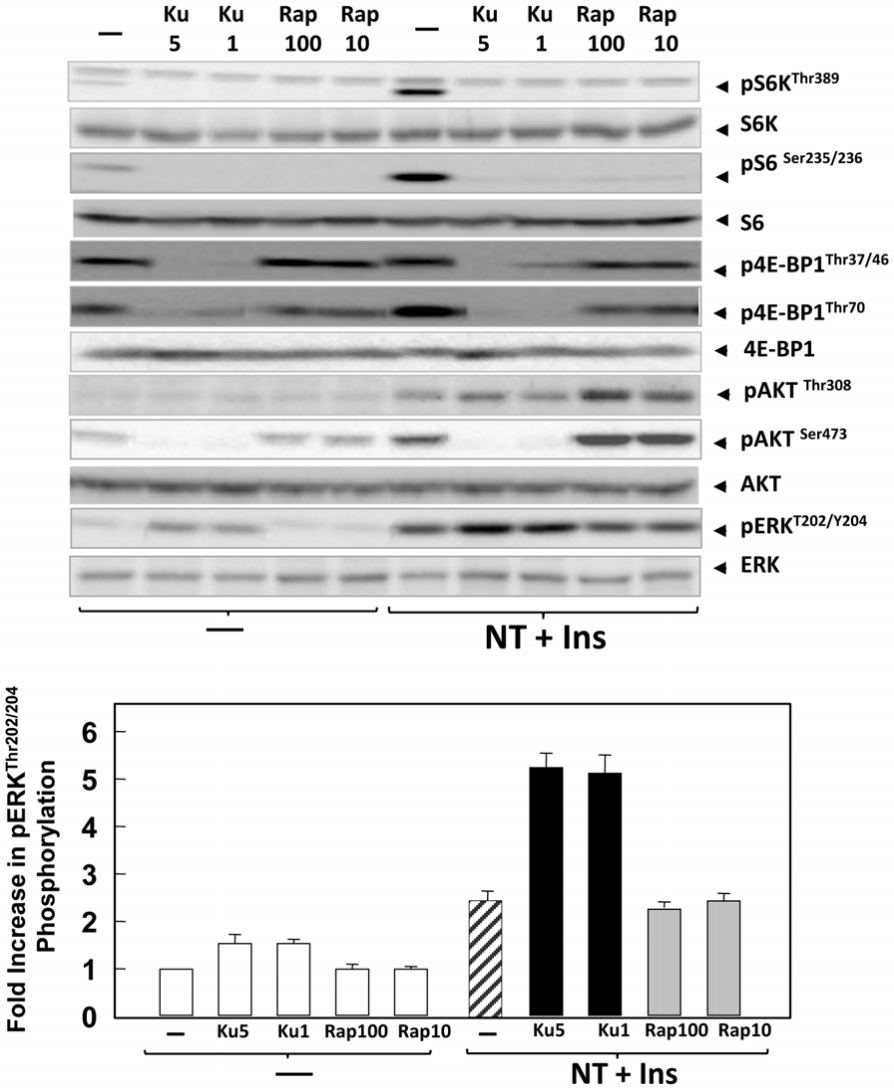
Differential feedback activation of Akt and ERK phosphorylation by rapamycin and KU63794 in MiaPaCa-2 cells. Cultures of MiaPaCa-2 cells were incubated in the absence (−) or in the presence of KU63794 (Ku) at 1 µM or 5 µM or rapamycin (Rap) at 10 or 100 nM for 2 h in DMEM containing 5 mM glucose, as indicated. Then, the cells were stimulated for 2 h with 5 nM neurotensin (NT) and 10 ng/ml insulin (Ins) and lysed with 2×SDS–PAGE sample buffer. The samples were analyzed by SDS-PAGE and immunoblotting with antibodies that detect the phosphorylated state of S6K at Thr^389^ (pS6K), S6 at Ser^235/236^ (pS6), 4E-BP1 at Thr^37/46^ and Thr^70^, Akt at Ser^473^ and ERK at Thr^202^ and Tyr^204^. Immunoblotting with antibodies that recognize total S6K, S6, 4E-BP1, Akt and ERK was used to verify that the cell treatments did not change the total level of these proteins and confirm equal gel loading. Fold increase in ERK phosphorylation was quantified using Multi Gauge V3.0 and plotted as bars. Similar results were obtained in 3 independent experiments.

Initially, we determined the influence of rapamycin and KU63794 on mTORC1-mediated phosphorylation of S6K in PDAC cells. Rapamycin is an allosteric inhibitor of mTORC1 that acts via FKBP-12 whereas KU63794 is a highly specific ATP-competitive inhibitor of mTOR that inhibits both mTORC1 and mTORC2. Cultures of PANC-1 (**Fig. 1**) or MiaPaCa-2 (**Fig. 2**) cells were incubated for 2 h in the absence or presence of rapamycin (at 10 or 100 nM) or KU63794 (at 1 or 5 µM). Then, the cultures were stimulated with a combination of insulin (10 ng/ml) and the GPCR agonist neurotensin (5 nM) for 2 h to elicit positive crosstalk [18], [20]. Phosphorylation of S6K on Thr^389^, a direct target of mTORC1, and phosphorylation of S6 (Ser^235/236^), a substrate of S6K, was monitored using specific antibodies that detect the phosphorylated state of those residues. Stimulation of either PANC-1 or MiaPaCa-2 cells with insulin and neurotensin induced robust phosphorylation of S6K on Thr^389^ and S6 (**Figs. 1** and **2**). Exposure to either rapamycin or KU63794 completely prevented the increase in the phosphorylation of these proteins in response to stimulation by insulin and neurotensin in either PANC-1 (**Fig. 1**) or MiaPaCa-2 cells (**Fig. 2**). We verified that the total levels of S6K and S6 did not change in response to the treatments. The results indicate that allosteric or active-site inhibitors of mTOR potently blocked the mTORC1/S6K axis at the concentrations used in PDAC cells.

### Differential regulation of 4EBP1 phosphorylation sites in response to mitogenic stimulation, rapamycin and KU63794 in PDAC cells

The phosphorylation of 4EBP1 was also monitored by using site-specific 4E-BP1 antibodies that detect p-Thr^37/46^ or p-Thr^70^ in lysayes of PANC-1 (**Fig. 1**) or MiaPaCa-2 (**Fig. 2**) cells. These cells displayed a high basal level of 4E-BP1 phosphorylation at Thr^37/46^ that was not further increased by stimulation with insulin and neurotensin. However, cell stimulation reduced the mobility of 4E-BP1 in SDS/PAGE, a response suggestive of increased phosphorylation at other sites. Indeed, treatment of PANC-1 or MiaPaCa-2 cells with neurotensin and insulin markedly stimulated 4E-BP1phosphorylation on Thr^70^. The constituve phosphorylation of 4E-BP1 on Thr^37/46^ was abolished by treatment with KU63794 but was not affected by rapamycin at either 10 or 100 nM, in agreement with reports that rapamycin and its analogs do not inhibit 4E-BP1 phosphorylation at these sites in other cell types. In contrast, the signal responsive phosphorylation of 4E-BP1 on Thr^70^ was prevented by treatment with either KU63794 or rapamycin at 100 nM. We verified that the total levels of 4E-BP1 did not change in response to the treatments.These results revealed an unappreciated regulation of 4E-BP1 phosphorylation on different residues in response to external signals and demonstrate that rapamycin inhibits inducible but not constitutive 4E-BP1 phosphorylation in PDAC cells whereas active-site mTOR inhibitors suppress phosphorylation of 4E-BP1 at all sites.

### Rapamycin and KU63794 induce over-stimulation of different upstream pathways in PDAC cells stimulated with insulin and neurotensin or insulin alone

In order to determine whether allosteric and active-site mTOR inhibitors eliminate feedback loops that restrain the activity of upstream signaling pathways in PDAC cells, we examined the effect of these inhibitors on the phosphorylation of Akt in response to mitogenic signaling in PANC-1 and Mia PaCa-2 cells. Stimulation of these cells with insulin and neurotensin induced a marked increase in Akt phosphorylation on Ser^473^ (**Figs. 1** and **Fig. 2**). Treatment with either 10 nM or 100 nM rapamycin promoted over-stimulation of Akt phosphorylation on Ser^473^, consistent with suppression of mTORC1/S6K axis feedback loops. In contrast, prior exposure to the active-site mTOR inhibitor KU63794, which inhibits both mTORC1 and mTORC2, blocked Akt phosphorylation on Ser^473^ in PANC-1 (**Fig. 1**) and MiaPaCa-2 cells (**Fig. 2**), in line with the notion that mTORC2 is the major protein kinase that phosphorylates Akt on Ser^473^ in PDAC cells. KU63794 did not prevent Akt phosphorylation at Thr^308^.

The ERK/RSK pathway, which plays a pivotal role in PDAC cell proliferation also leads to mTORC1 activation [5], [62]. In breast and bladder cancer cells, inhibition of the mTORC1/S6K axis by rapamycin induced feedback activation of ERK [63]. Consequently, we examined the effects of rapamycin and KU63794 on ERK activation in PDAC cells. In agreement with previous studies [16], [64], [65], stimulation of either PANC-1 or MiaPaCa-2 cells with insulin and neurotensin markedly activated ERK (ERK phosphorylated on Thr^202^ and Tyr^204^), as illustrated in **Figs. 1** and **2**. In contrast to the results obtained in other cell types [63], treatment with either 10 or 100 nM rapamycin for 2 h did not alter the basal or the stimulated level of ERK phosphorylation in PANC-1 and MiaPaCa-2 cells. Similar results were obtained when these PDAC cells were treated with rapamycin for 4 or 24 h (results not shown). In contrast, exposure to KU63794 (1–5 µM) increased the basal level of ERK phosphorylation and strikingly enhanced the stimulation of ERK phosphorylation induced by insulin and neurotensin in either PANC-1 or MiaPaCa-2 cells. Quantification of the results with ERK is illustrated in the lower panels of **Figs. 1** and **2** (bars). These results demonstrate that rapamycin, an allosteric inhibitor of mTORC1, and KU63794, an active-site inhibitor of mTOR, lead to over-activation of different upstream pro-oncogenic pathways in PDAC cells.

**Figure 3:**
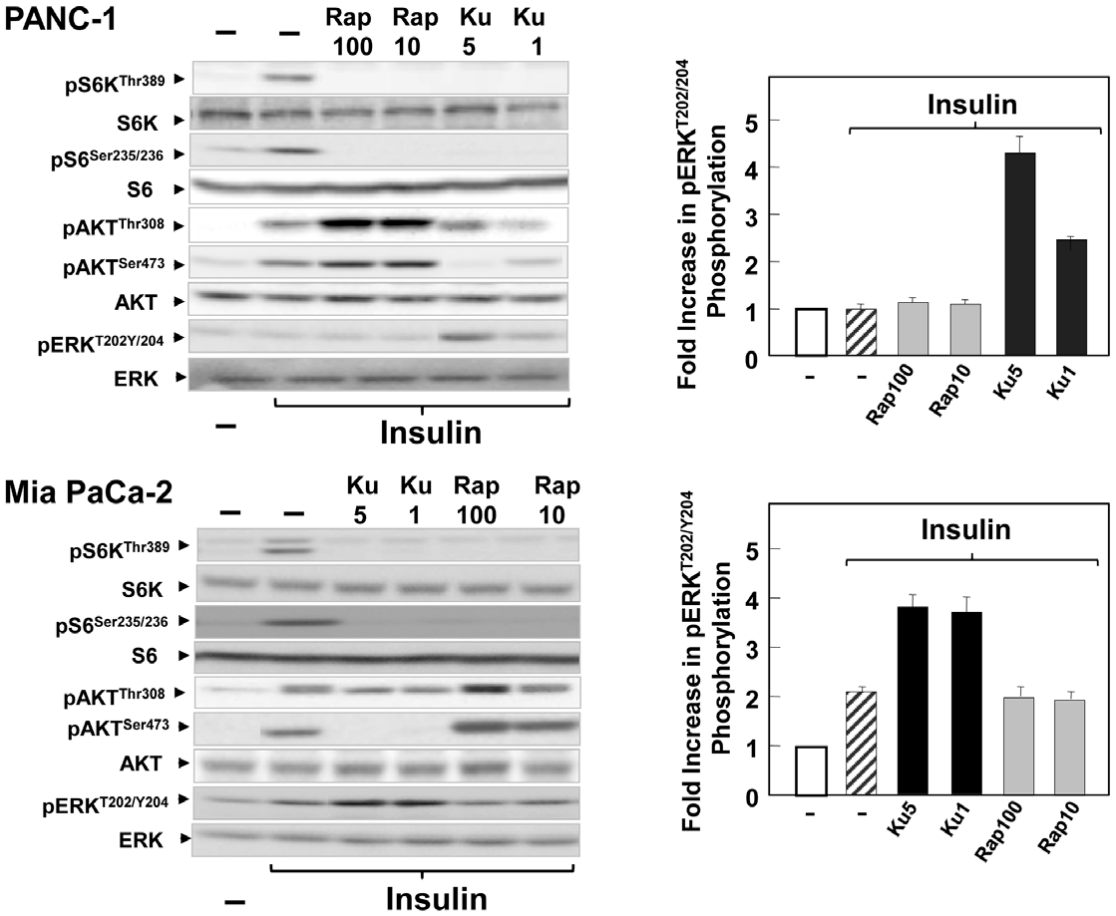
Differential feedback activation of Akt and ERK phosphorylation by rapamycin and KU63794 in insulin-stimulated MiaPaCa-2 and PANC-1 cells. The cultures of PANC-1 (upper panels) and MiaPaCa-2 (lower panels) were incubated in the absence (−) or in the presence of KU63794 (Ku) at 1 µM or 5 µM or rapamycin (Rap) at 10 or 100 nM for 2 h in DMEM containing 5 mM glucose, as indicated. Then, the cells were stimulated for 2 h with 10 ng/ml insulin and lysed with 2×SDS–PAGE sample buffer. The samples were analyzed by SDS-PAGE and immunoblotting with antibodies that detect the phosphorylated state of S6K at Thr^389^, S6 at Ser^235/236^, Akt at Ser^473^ and Thr^308^ and ERK at Thr^202^ and Tyr^204^. Immunoblotting with total S6K, S6, Akt and ERK was used to verify equal gel loading. Fold increase in ERK phosphorylation was quantified using Multi Gauge V3.0 and plotted as bars. Similar results were obtained in 3 independent experiments.

Stimulation of PANC-1 cells or MiaPaCa-2 with insulin alone induced robust increase in PI3K/Akt/mTORC1 but does not induce significant increase in ERK phosphorylated on Thr^202^ and Tyr^204^ (**Fig. 3**). Consequently, we determined whether the differential effects of rapamycin and KU63794 depicted in PDAC stimulated with the combination of insulin and neurotensin (Figs. 1 and 2) can also be produced when PANC-1 and MiaPaCa-2 cells are challenged with insulin alone. Cultures of these cells were incubated for 2 h in the absence or presence of rapamycin (10–100 nM) or KU63794 (1–5 µM) and then stimulated with insulin (10 ng/ml). We monitored phosphorylation of S6K on Thr^389^, S6 on Ser^235/236^, Akt on Ser^473^ and Thr^308^ and ERK on Thr^202^ and Tyr^204^. Prior exposure to either rapamycin or KU63794 abolished the increase in the phosphorylation of S6K and S6 in response to insulin in either PANC-1 or MiaPaCa-2 cells (**Fig. 3**). Exposure to rapamycin over-activated whereas treatment with KU63794 abolished Akt phosphorylation on Ser^473^ in the insulin-stimulated PDAC cells. Rapamycin did not produce any detectable effect on ERK activation in un-stimulated or insulin-treated cells. A salient feature of the results shown in **Fig. 3** is that exposure to KU63794 induced a marked increase in the phosphorylation of ERK on Thr^202^ and Tyr^204^. These results corroborated that the allosteric inhibitor of mTORC1 and the active-site site inhibitor of mTOR promote over-activation of different upstream pathways in PDAC cells challenged with insulin or insulin and neurotensin, a combination that elicits crosstalk between insulin/IGF and GPCR signaling systems.

### PP242, like KU63794, enhances ERK activation in PANC-1 cells stimulated with insulin and neurotensin

**Figure 4:**
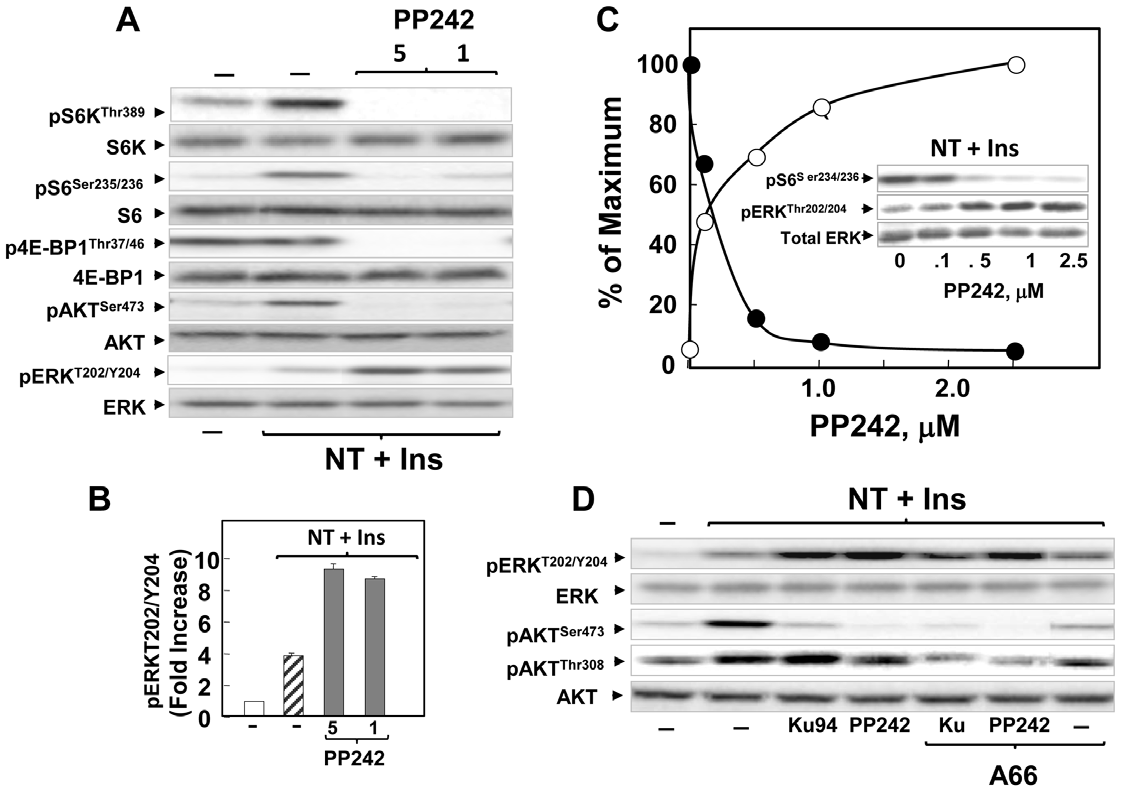
Feedback activation of ERK phosphorylation by PP242: role of PI3K. ***A*** **,** Cultures of PANC-1 cells were incubated in the absence (−) or in the presence of PP242 at 1 µM or 5 µM for 2 h in DMEM containing 5 mM glucose, as indicated. Then, the cells were stimulated for 2 h with 5 nM neurotensin (NT) and 10 ng/ml insulin (Ins) and lysed with 2×SDS–PAGE sample buffer. The samples were analyzed by SDS-PAGE and immunoblotting with antibodies that detect the phosphorylated state of S6K at Thr^389^, S6 at Ser^235/236^, 4E-BP1 at Thr^37/46^, Akt at Ser^473^ and ERK at Thr^202^ and Tyr^204^. Immunoblotting with antibodies that recognize total S6K, S6, 4E-BP1, Akt and ERK was used to verify that the cell treatments did not change the total level of these proteins and confirm equal gel loading. Similar results were obtained in 3 independent experiments. ***B*** **,** The bars represent the increase in ERK phosphorylation induced by insulin (Ins) and neurotensin (NT) in cells without or with prior exposure to PP242. Quantification was performed using Multi Gauge V3.0 ***C,*** Cultures of PANC-1 cells were incubated as in panel **A** but in the presence of increasing concentrations of PP242. The samples were analyzed to detect the phosphorylated state of S6 at Ser^235/236^ and ERK at Thr^202^ and Tyr^204^. Immunoblotting with total ERK and S6 (not shown) was used to verify equal gel loading. Quantification was performed using Multi Gauge V3.0. ***D*** **,** Cultures of PANC-1 cells were incubated in the absence (−) or in the presence of either KU63794 (Ku) or PP242 at 5 µM for 2 h. Then, the cells were stimulated for 2 h with 5 nM neurotensin (NT) and 10 ng/ml insulin (Ins) and lysed with 2×SDS–PAGE sample buffer. The samples were analyzed by SDS-PAGE and immunoblotting with antibodies that detect the phosphorylated state of ERK at Thr^202^ and Tyr^204^, Akt at Ser^473^ and Thr^308^. Immunoblotting with total Akt and ERK was used to verify equal gel loading.

Subsequently, we determined whether the striking over-activation of ERK by the active-site mTOR inhibitor KU63794 could be also produced by a structurally unrelated active-site mTOR inhibitor. Cultures of PANC-1 were incubated for 2 h in the absence or presence of PP242 (1–5 µM), a recently identified active-site mTOR inhibitor [37], and stimulated for 2 h with insulin and neurotensin. We monitored phosphorylation of S6K on Thr^389^, S6 on Ser^235/236^, 4EBP1 on Thr^37/46^, Akt on Ser^473^ and ERK on Thr^202^ and Tyr^204^. As shown in **Fig. 4 A**, prior exposure to PP242 abolished the phosphorylation of S6K, S6, 4EBP1 and Akt in PANC-1 cells. The key feature of the results is that PP242, like KU63794, induced a marked increase in the phosphorylation of ERK on Thr^202^ and Tyr^204^ (**Fig. 4A** and quantification in **Fig. 4B**). Because PP242 is a less selective mTOR inhibitor [66], we determined whether the concentrations of PP242 that promoted ERK activation coincide with those that inhibit mTORC1 activity. As shown in **Fig. 4C**, PP242 enhanced ERK activation and inhibited S6 phosphorylation at almost identical concentrations.

We verified that the active-site mTOR inhibitors KU63794 and PP242, at concentrations that markedly enhanced ERK activation and inhibited Akt phosphorylation on Ser^473^ did not prevent Akt phosphorylation at Thr^308^ in PDAC cells (**Fig. 4D**). In fact, the specific mTOR inhibitor KU63794 slightly enhanced Akt phosphorylation at Thr^308^, consistent with suppression of feedback loops that restrain PI3K activity (**Fig. 4D**). PP242 was less effective than KU63794 in enhancing Akt phosphorylation at Thr^308^, most likely reflecting off-target inhibition of PI3K [66]. Thus, the specific active-site site mTOR inhibitors KU63794 and PP242 suppressed Akt phosphorylation on Ser^473^, did not decrease Akt phosphorylation on Thr^308^ and stimulated over-activation of ERK phosphorylation at Thr^202^ and Tyr^204^ in PDAC cells.

The mechanism by which active-site inhibitors enhance ERK activation is not well understood. Our results do not support the existence in PDAC cells of a putative mTORC1/S6K/PI3K/ERK feedback loop, proposed in other cell types [63], since potent inhibition of the mTORC1/S6K axis by either rapamycin or everolimus did not produce overstimulation of ERK in PDAC cells. To substantiate this conclusion, PANC-1 cells were treated with KU63794 or PP242 and stimulated with insulin and neurotensin in the absence or presence of A66 [67], a selective inhibitor of the 110α catalytic subunit of PI3K. As shown in **Fig. 4D**, exposure to A66 did not prevent enhancement of ERK activation in response to exposure to either KU63794 or PP242. We corroborated that A66, at the concentration used, potently inhibited PI3K within PANC-1 cells since it prevented insulin-induced Akt phosphorylation at Thr^308^, the key residue in the Akt activation loop phosphorylated by PI3K-dependent PDK1.

In order to obtain further insight of the mechanism by which treatment with KU63794 induces over-activation of ERK we also determined the effect of this active-site mTOR inhibitor on the activation of MEK, the upstream kinase that phosphorylates ERK. MEK activation was scored by assessing the phosphorylation of Ser^217^ and Ser^221^ in its activation loop. As shown in **Fig. S1**, treatment of PANC-1 or MiaPaCa-2 cells with KU63794 markedly enhanced MEK phosphorylation induced by insulin and neurotensin. Collectively, the results demonstrate that active-site mTOR inhibitors led to MEK/ERK hyper-activation through a PI3K/S6K-independent feedback loop in PDAC cells.

### Differential patterns of Akt and ERK activation in response to rapamycin, everolimus, KU63794 and PP242 in PANC-1 cells stimulated with serum

**Figure 5:**
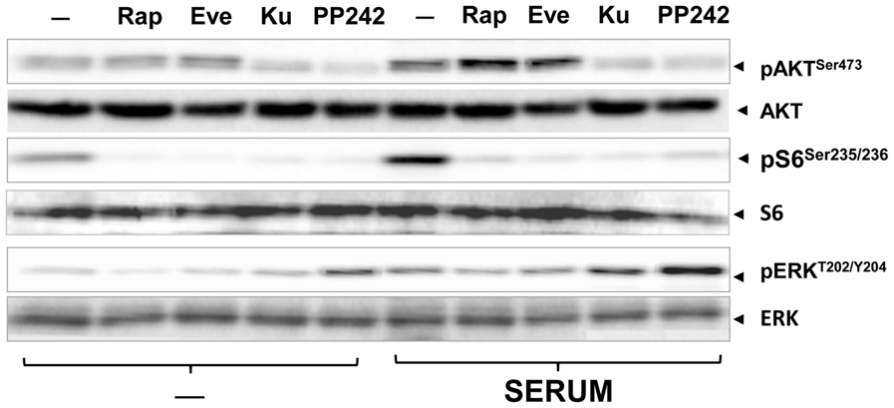
Differential feedback activation of Akt and ERK by rapamycin, everolimus, KU63794 and PP242 in serum-stimulated PANC-1 cells. The cultures were incubated in the absence (−) or in the presence of 5 µM KU63794 (Ku), 5 µM PP242, 100 nM rapamycin (Rap) or 100 nM everolimus for 2 h in DMEM containing 5 mM glucose, as indicated. Then, the cells were stimulated for 2 h with fetal bovine at a final dilution of 2% (SERUM) and lysed with 2×SDS–PAGE sample buffer. The samples were analyzed by SDS-PAGE and immunoblotting with antibodies that detect the phosphorylated state of Akt at Ser^473^, S6 at Ser^235/236^, and ERK at Thr^202^ and Tyr^204^. Immunoblotting with total Akt, S6 and ERK was used to verify equal gel loading. Similar results were obtained in 3 independent experiments.

The preceding results were obtained with PDAC cells stimulated with defined mitogens that act through specific receptors. To extend further these findings we also tested whether differential patterns of Akt and ERK activation are produced when the cells are stimulated with fetal bovine serum. Cultures of PANC-1 cells were incubated for 2 h in the absence or presence of rapamycin (100 nM), everolimus (100 nM), KU63794 (1 µM) or PP242 (1 µM) and stimulated with medium containing fetal bovine serum. We monitored phosphorylation of S6 on Ser^235/236^, Akt on Ser^473^ and ERK on Thr^202^ and Tyr^204^. Prior exposure to rapamycin, everolimus, KU63794 or PP242 abolished the increase in the phosphorylation of S6 in response to serum (**Fig. 5**). Exposure to rapamycin or everolimus over-activated whereas treatment with KU63794 or PP242 abolished Akt phosphorylation on Ser^473^ in serum-stimulated PDAC cells. Rapamycin or everolimus did not produce any detectable effect on ERK activation whereas exposure to KU63794 or PP242 induced a marked increase in the phosphorylation of ERK on Thr^202^ and Tyr^204^ in serum-treated cells (**Fig. 5**). These results corroborated that allosteric and active-site site inhibitors of mTOR promote over-activation of different upstream pathways in PDAC cells under a variety of experimental conditions, including cells challenged with insulin, insulin and the GPCR agonist neurotensin or with fresh fetal bovine serum.

### Metformin, in contrast to allosteric and active-site mTOR inhibitors, inhibits ERK activation and does not induce over-stimulation of Akt in PDAC cells

**Figure 6:**
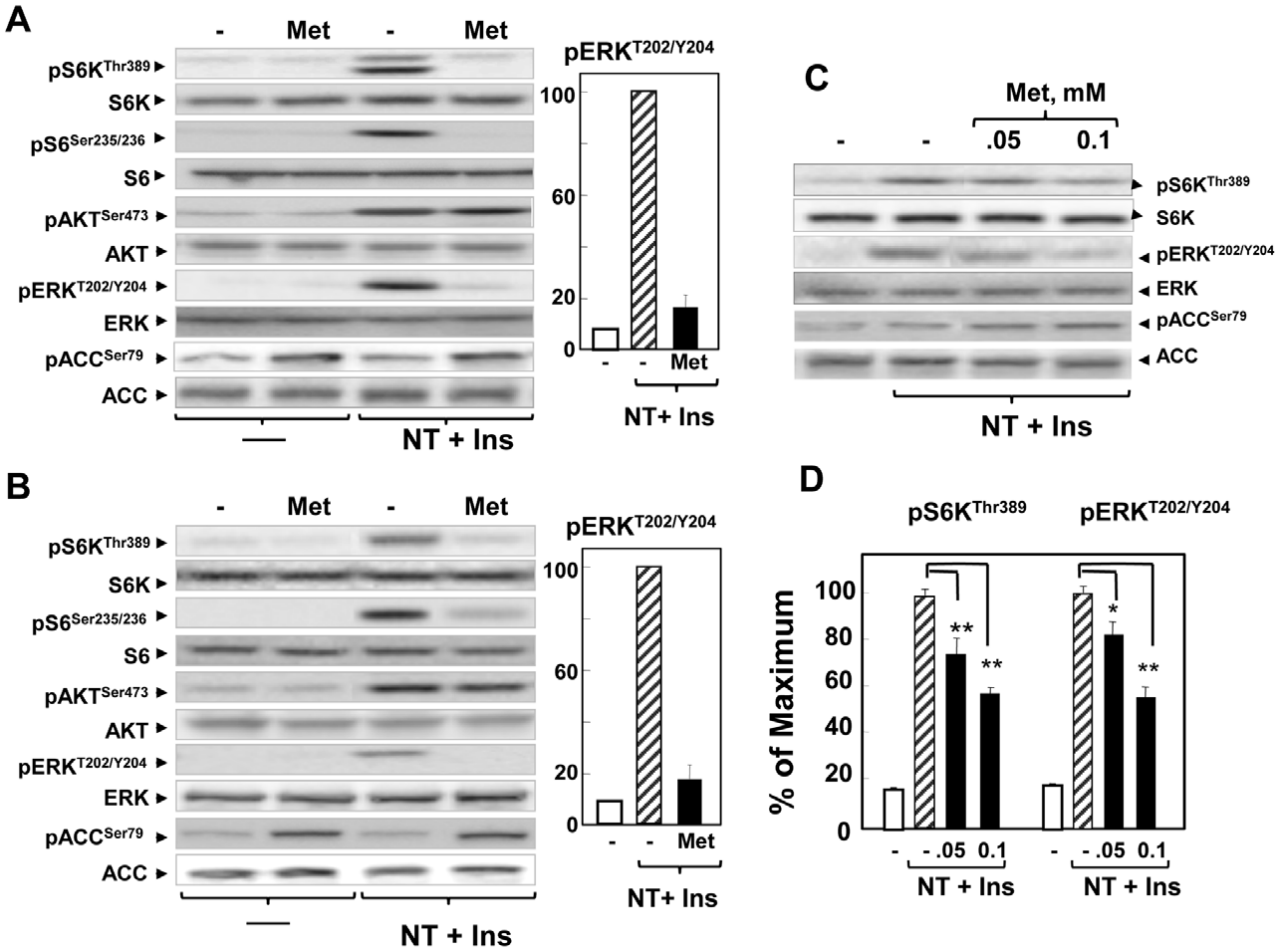
Metformin inhibits mTORC1 and ERK signaling without over-activating Akt in PDAC cells incubated in medium containing a physiological glucose concentration. **A**) Cultures of MiaPaca-2 (**A**) and PANC-1 (**B**) cells were incubated in the absence (−) or in the presence of 1 mM metformin (Met) for 16 h in DMEM containing 5 mM glucose, as indicated. Then, the cells were stimulated for 2 h with 5 nM neurotensin and 10 ng/ml insulin (NT+Ins) and lysed with 2×SDS–PAGE sample buffer. The samples were analyzed by SDS-PAGE and immunoblotting with antibodies that detect the phosphorylated state of S6K at Thr^389^, S6 at Ser^235/236^, ACC at Ser^79^, Akt at Ser^473^ and ERK at Thr^202^ and Tyr^204^. Immunoblotting with antibodies that recognize total S6K, S6, Akt, ERK and ACC was used to verify that the cell treatments did not change the total level of these proteins and confirm equal gel loading. Similar results were obtained in 3 independent experiments. The bars in panels ***A*** and ***B*** represent the % of the maximal ERK phosphorylation (mean ±SEM) induced by insulin (Ins) and neurotensin (NT) in cells without or with prior treatment with 1 mM metformin. The results of ERK phosphorylation were obtained in multiple independent experiments (N = 12 for PANC-1 and N = 8 for MiaPaca-2) Quantification was performed using Multi Gauge V3.0 **C**). Mia PaCa-2 cells were incubated with DMEM containing 5 mM glucose either in absence or presence of 0.05 mM or 0.1 mM metformin for 16 h. Then, the cells were treated with NT+Ins, as above, and lysates analyzed by immunoblotting. Similar results were obtained in 6 independent experiments. **D**) The experiment presented in panel C was representative of 6 independent experiments. Quantification of these experiments was performed using Multi Gauge V3.0. Results are expressed as the percentage of maximum mean ±SEM, n = 6. P values were determined using the t-test (Sigma Plot 12.) *p<0.05; **p<0.001; n = 6.

Like rapamycin and active-site mTOR inhibitors, metformin also inhibits stimulation of the mTORC1/S6K axis but its effects on feedback loops regulating Akt and ERK activation have not been examined in PDAC cells. Recently, we demonstrated that the sensitivity of PDAC cells to the inhibitory effects of metformin are markedly enhanced by culturing PDAC cells in medium containing physiological (5 mM) rather than supra-physiological (25 mM) concentrations of glucose [68]. In order to determine the effect of metformin on Akt and ERK signaling in PDAC cells, PANC-1 and MiaPaCa-2 cells grown in medium containing 5 mM glucose were treated with or without metformin (1 mM) and then stimulated with insulin and the GPCR agonist neurotensin. mTORC1 activity was determined by phosphorylation of S6K at Thr^389^ and phosphorylation of S6 (Ser^235/236^) and ERK activation by detecting ERK phosphorylated on Thr^202^ and Tyr^204^. Metformin, like rapamycin, virtually abolished mTORC1 activation induced by insulin and neurotensin in PANC-1 and MiaPaCa-2 cells (pS6K, pS6 in **Fig. 6, A** and **B**) without changing the total levels of either S6K or S6. However, metformin did not over-stimulated Akt phosphorylation on Ser^473^ in the PDAC cells (p-Akt^473^ in **Fig. 6, A** and **B**), a result strikingly different from that obtained with rapamycin and everolimus. The salient feature of the results in **Fig. 6 A** and **B** is that metformin, in sharp contrast to the effects of active-site mTOR inhibitors, prevented ERK activation in PANC-1 and MiaPaCa-2 cells in multiple independent experiments (depicted by the bars) but did not alter the level of total ERK. We verified that under these experimental conditions, metformin markedly induced AMPK activation, as shown by the phosphorylation of acetyl-CoA carboxylase (ACC) at Ser^79^, a residue directly phosphorylated by AMPK and used as a biomarker of its activity within intact cells.

We next determined whether metformin inhibits ERK activation at concentrations (0.05–0.1 mM) that are close to the therapeutic range. As shown in **Fig. 6 C**, metformin dose-dependently inhibited phosphorylation S6K at Thr^389^ and ERK activation at concentrations as low as 0.05–0.1 mM. Metformin, at these concentrations, also induced AMPK activation, as shown by ACC phosphorylation at Ser^79^. Quantification of the immunoblotting results is illustrated in **Fig. 6 D**. Our results demonstrate that the effects of metformin on Akt and ERK activation are strikingly different from those elicited by allosteric or active-site mTOR inhibitors, though all these agents potently inhibited the mTORC1/S6K axis.

### Effects of metformin, KU63974 and rapamycin on the proliferation of PANC-1 cells

**Figure 7:**
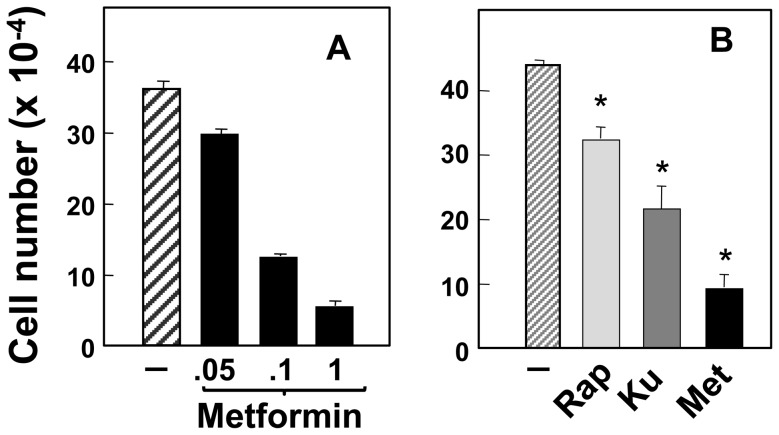
Metformin inhibits PANC-1 cell proliferation more potently than rapamycin or KU63794. ***A***, Single-cell suspensions of PANC-1 cells were plated at a density of 10^5^ cells per dish. After 24 h, the cultures were shifted to media containing 0.25% FBS without (−) or with 10 nM neurotensin and 10 ng/ml insulin (NT+Ins) in the absence (open bars) or presence (closed bars) of increasing concentrations of metformin, as indicated. After 4 days, cell numbers were determined from 6 plates per condition. Results are presented as mean ± SEM. Similar results were obtained in two independent experiments. p values compared to control were all <0.05 n = 6. ***B,*** Single-cell suspensions of PANC-1 cells were plated at a density of 10^5^ cells per dish. After 24 h, the cultures were shifted to media containing 2.5% FBS without (−) or with 1 mM metformin, 5 µM KU63794 (Ku) or 100 nM rapamycin (Rap) as indicated. After 4 days, cell numbers were determined from 6–8 plates per condition. Results are presented as mean ± SEM. Similar results were obtained in two independent experiments. * All p values compared to control were <0.05 n = 6. Anova analysis showed that metformin inhibition of cell proliferation was statistically significant (<p<0.05) from either rapamycin or KU63794. In turn, KU63794 was statistically different from rapamycin (p<0.05).

The differential effects of metformin, KU63794 and rapamycin on the activation of PI3K/Akt and MEK/ERK in PDAC cells, prompted us to determine the effects of these agents on the proliferation of these cells. Initially, we assessed the effect of increasing concentrations of metformin on the increase in the number of PANC-1 cells induced by stimulation with neurotensin and insulin in the presence of 0.25% serum for 4 days (**Fig. 7, A**). Metformin prevented the increase in the number of PANC-1 cells in a dose-dependent manner. A marked inhibitory effect was induced by metformin at a concentration as low as 0.1 mM and complete suppression of cell proliferation was achieved by metformin at 1 mM. The concentrations of metformin that inhibited PANC-1 cell proliferation coincided with the concentration of metfornin that prevented mTORC1 and ERK signaling in these cells.

Next, we examined the effect of 1 mM metformin, 5 µM KU63794 and 100 nM rapamycin on the proliferation of PANC-1 incubated in medium containing serum. Each agent was tested at a concentration that produced maximal inhibition of the mTORC1/S6K axis in PDAC cells. As seen in **Fig. 7 B**, the agents inhibited PANC-1 cell proliferation but with important differences in their efficacy. Metformin induced a more pronounced inhibition of proliferation than either KU63794 or rapamycin while, the active-site mTOR inhibitor was more effective than rapamycin (all these differences were statistically significative). The results suggest that the effects of inhibiting mTORC1/S6K by the allosteric or active-site inhibitors is compensated by over-activation of Akt (rapamycin) or ERK (KU63794). The comparatively stronger inhibition of PDAC cell proliferation by metformin could be attributed, at least in part, to inhibition of ERK signaling.

## Discussion

Aberrant stimulation of the mTOR pathway in many cancer cells, including PDAC, is eliciting intense interest for targeting this pathway [1]. However, it is increasingly appreciated that the mTORC1/S6K axis also mediates negative feedback loops that attenuate signaling via insulin/IGF receptor and other tyrosine kinase receptors. Suppression of these feed-back loops unleashes over-activation of upstream pathways that potentially counterbalance the anti-proliferative effects of mTOR inhibitors. Consequently, the identification of negative feedback loops by either allosteric or active-site mTOR inhibitors has emerged as an area of major interest in cancer therapy. Because the operation of these complex feedback loops is cell-context specific, we examined the patterns of Akt and ERK feedback activation in response to mTORC1 inhibition by rapamycin, active-site mTOR inhibitors and metformin in human PDAC cells. PDAC is one of the most lethal human diseases, with overall 5-year survival rate of only 3–5% and a median survival period of 4–6 months. The incidence of this disease in the US has increased to more than 44,000 new cases in 2011 and is now the fourth leading cause of cancer mortality in both men and women [69]. As the current therapies offer very limited survival benefits, novel molecular therapeutic targets and strategies are urgently needed to treat this aggressive disease.

Our results demonstrate that treatment of PDAC cells with allosteric mTORC1 inhibitors (rapamycin, everolimus) augmented Akt phosphorylation at Ser^473^ while the active-site inhibitors of mTOR (KU63794 and PP242) completely abrogated Akt phosphorylation at this site consistent with the notion that mTORC2 is the major kinase that phosphorylates Akt at Ser^473^. A salient feature of our results is that active-site inhibitors of mTOR promoted a marked increase in ERK activation in PDAC cells stimulated with insulin, insulin and neurotensin or serum. These results indicate that first and second generations of mTOR inhibitors promote over-activation of different upstream pro-oncogenic pathways in PDAC cells.

While augmentation of Akt phosphorylation at Ser^473^ by rapalogs is well known in other cell types [30], [33]–[35], the enhancing effect of active-site mTOR inhibitors on ERK has been much less explored. In order to understand the mechanism by which active-site mTOR inhibitors promote ERK activation in PDAC cells, we determined here the role of a feedback loop involving mTORC1/S6K/PI3K/ERK, proposed to mediate ERK activation in other cell types [63]. Several lines of evidence dissociated this feedback loop from the enhancement of ERK activation induced by active-site mTOR inhibitors in PDAC cells. Firstly, neither rapamycin nor everolimus, at concentrations that completely blocked the mTORC1/S6K axis, produced any detectable enhancement of ERK activation in PDAC cells under a variety of experimental conditions. Secondly, KU63794 induced ERK hyper-activation even in PDAC cells treated with A66, a potent and selective inhibitor of the 110α catalytic subunit of PI3K [67]. These results, indicating that active-site inhibitors enhance ERK through a PI3K-independent pathway, are in agreement with a recent report using PP242 in multiple myeloma cells [70]. However, PP242 inhibits a number of protein kinases *in vitro*, including MEK, whereas KU63794 did not inhibit any protein kinase other than mTOR [66]. The possibility that PP242 could induce ERK via off-target effects is an important consideration. Our results demonstrate, for the first time, that the highly selective inhibitor of mTOR KU63794 enhances MEK/ERK activation through a PI3K-independent pathway. Given that active-site mTOR inhibitors are increasingly considered for clinical use [1], the findings presented here imply that suppression of feed-back loops by these inhibitors should be a major consideration in the use of these inhibitors for PDAC therapy.

Many epidemiological studies have linked obesity and long-standing type 2 diabetes mellitus (T2DM), with increased risk for developing PDAC and other clinically aggressive cancer [71], [72]. Obesity and T2DM are multifaceted but characterized by peripheral insulin resistance and compensatory overproduction of insulin by the β cells of the islet leading to increase circulating levels of insulin and enhanced bioavailability of IGF-1. Further, epidemiological studies are linking administration of metformin, the most widely used drug in the treatment of T2DM, with reduced incidence, recurrence and mortality of a variety of cancers in T2DM patients [20], [47]–[56], including PDAC. Indeed, T2DM patients who had taken metformin had a 62% lower adjusted incidence of PDAC compared with those who had not taken metformin [54], a result recently substantiated in a different patient population [56]. Here we demonstrate that metformin abolishes mTORC1/S6K activation in PDAC cells but in contrast to rapamycin, metformin treatment did not overactivate Akt phosphorylation on Ser^473^. We verified that, under our conditions, metformin stimulated AMPK in PDAC cells. In this context it is relevant that AMPK not only blocks mTORC1 activation but also mediates IRS-1 phosphorylation at Ser^794^, an inhibitory site that attenuates PI3K/Akt activation [29], [73]. We confirmed that metformin (but not rapamycin) induced IRS-1 phosphorylation at Ser^794^ in PANC-1 cells (our unpublished results). Consequently, it is plausible that metformin, via AMPK-mediated phosphorylation of IRS-1 at Ser^794^, attenuates PI3K overstimulation caused by interrupting feedback loops mediated by the mTOR/S6K axis and thereby avoids hyper-activation of Akt phosphorylation on Ser^473^. More importantly, we found that metformin inhibited rather than enhanced ERK activation (like active-site mTOR inhibitors) in PDAC cells. The inhibitory effects of metformin on ERK were elicited at low concentrations when the cells were cultured in medium containing physiological concentrations of glucose [68]. Thus, the effects of metformin on Akt phosphorylation and ERK activation are strikingly different from allosteric or active-site mTOR inhibitors, though all these agents potently inhibited the mTORC1/S6K axis. In line with differential effects on feedback loops, further findings revealed that metformin inhibited cell proliferation of PDAC cells more efficiently than allosteric or active-site mTOR inhibitors in PDAC cells. Although the elucidation of the precise mechanism(s) involved requires further experimental work, it is tempting to speculate that the favorable effects of metformin on preventing over-activation of pro-oncogenic pathways, such as Akt and ERK, may contribute to its antiproliferative effects *in vitro* and ultimately explain its beneficial anticancer effects.

## Supporting Information

Figure S1 **Treatment with KU63794 causes over-activation of MEK and ERK phosphorylation in PANC-1 and MiaPaCa-2 cells.** The cultures of PANC-1 and MiaPaCa-2 were incubated in the absence (−) or in the presence of KU63794 (Ku) at 5 mM for 2 h in DMEM containing 5 mM glucose, as indicated. Then, the cells were stimulated with 10 ng/ml insulin and 5 nM neurotensin (NT+Ins) for 2 h and lysed with 2×SDS–PAGE sample buffer. The samples were analyzed by SDS-PAGE and immunoblotting with antibodies that detect the phosphorylated state of MEK at Ser^217/221^, ERK at Thr^202^ and Tyr^204^ and Akt at Ser^473^ Immunoblotting with total MEK, ERK and Akt was used to verify equal gel loading.
(TIF)
