# Ex pression of Concern: Different Patterns of Akt and ERK Feedback Activation in Response to Rapamycin, Active-Site mTOR Inhibitors and Metformin in Pancreatic Cancer Cells

**DOI:** 10.1371/journal.pone.0292422

**Published:** 2023-09-28

**Authors:** The PLOS ONE Editors

Following the publication of this article [1], concerns were raised regarding multiple figures. Specifically,

- Lanes 1–4 of the ERK panel in Fig 3 (PANC-1) appear similar to the ERK panel in Fig 6C despite representing different conditions.
- The corresponding author acknowledged that the following panels are spliced:
  ○ Fig 1, pAKT^Ser473^ and pERK^T202/Y204^.
  ○ Fig 2, p4E-BP1^Thr37/46^, p4E-BP1^Thr70^, pAKT^Ser473^, pERK^T202/Y204^.
  ○ Fig 3 (Panc-1), pS6K^Thr389^, pAKT^Ser473^, pERK^T202/Y204^.
  ○ Fig 3 (Mia PaCa-2), pAKT^Ser473^ and pERK^T202/Y204^.
  ○ Fig 4A, pERK^T202/Y204^.
  ○ Fig 4D, pERK^T202/Y204^, pAKT^Ser473^, and pAKT^Thr308^.
  ○ Fig 6B, pS6K^Thr389^, pS6^Ser235/236^, and pAKT^Ser473^.
  ○ Fig 6C, pS6K^Thr389^, pERK^T202/Y204^ and pACC^Ser79^.

**Fig 3:**
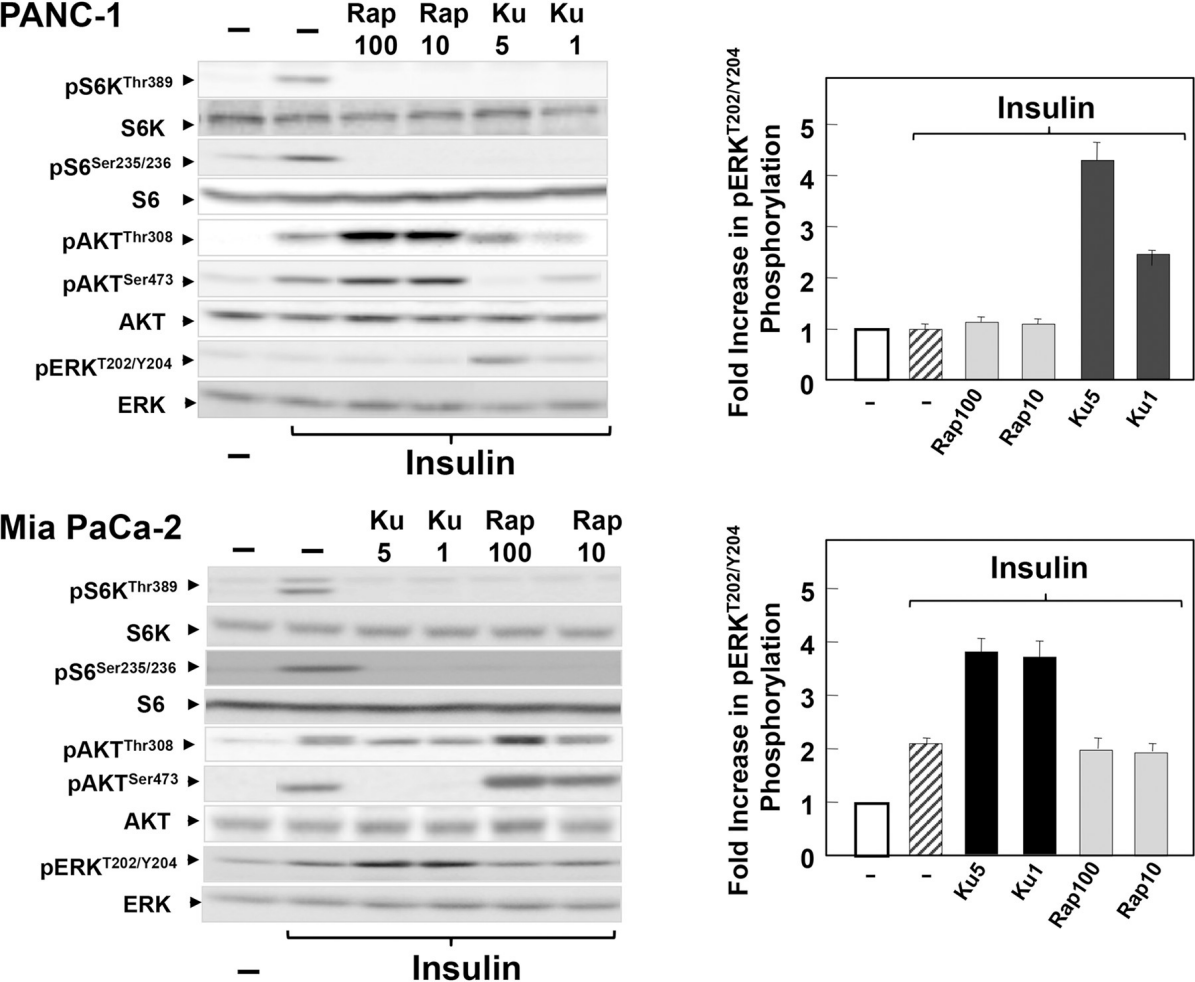
Differential feedback activation of Akt and ERK phosphorylation by rapamycin and KU63794 in insulin-stimulated MiaPaCa-2 and PANC-1 cells. The cultures of PANC-1 (upper panels) and MiaPaCa-2 (lower panels) were incubated in the absence (−) or in the presence of KU63794 (Ku) at 1 μM or 5 μM or rapamycin (Rap) at 10 or 100 nM for 2 h in DMEM containing 5 mM glucose, as indicated. Then, the cells were stimulated for 2 h with 10 ng/ml insulin and lysed with 2×SDS–PAGE sample buffer. The samples were analyzed by SDS-PAGE and immunoblotting with antibodies that detect the phosphorylated state of S6K at Thr^389^, S6 at Ser^235/236^, Akt at Ser^473^ and Thr^308^ and ERK at Thr^202^ and Tyr^204^. Immunoblotting with total S6K, S6, Akt and ERK was used to verify equal gel loading. Fold increase in ERK phosphorylation was quantified using Multi Gauge V3.0 and plotted as bars. Similar results were obtained in 3 independent experiments.

The corresponding author stated that the ERK panel in Fig 3 (PANC-1) is incorrect and provided a replacement that is spliced between lanes 4 and 5. An updated Fig 3 is provided with this notice and original uncropped images underlying all panels in this figure including the replacement ERK and excluding S6 are provided in S1 File.

The corresponding author provided uncropped western blot images underlying several figures, provided here in S1 File. They acknowledged that some of the underlying data is no longer available or cannot be accurately identified, and that panels grouped together were not always measured on the same blot or at the same time. For a small number of panels, the editors and authors were not in agreement that the original and published blots match, however the editors consider the results in the provided uncropped blots to support the corresponding published results. Concerns remain regarding the presentation of proteins together that were measured on different blots and the accuracy of data presentation. In the absence of all underlying data, these issues cannot be resolved.

The corresponding author acknowledged that some of the total protein panels in the figures were repeats, and not measured at the same time as the corresponding phosphorylated protein panels. They stated that phosphorylated protein expression was not normalized to total protein expression because they did not expect change in total protein expression in cell lines plated at the same density and measured at rapid timepoints. The editors remain concerned about the reliability of phosphorylated protein expression results.

The *PLOS ONE* Editors issue of this expression of concern to inform readers of the above concerns and provide the underlying data that remains available.

## Supporting information

S1 File The available original uncropped images underlying western blots in Figs 1, 2, 3, 4, 5, 6. (PDF)
