# Supplementary figures and images for: Different Patterns of Akt and ERK Feedback Activation in Response to Rapamycin, Active-Site mTOR Inhibitors and Metformin in Pancreatic Cancer Cells
Source: PLoS One. 2013 Feb 21;8(2):e57289. doi: 10.1371/journal.pone.0057289 (PMC3578870; doi:10.1371/journal.pone.0057289)

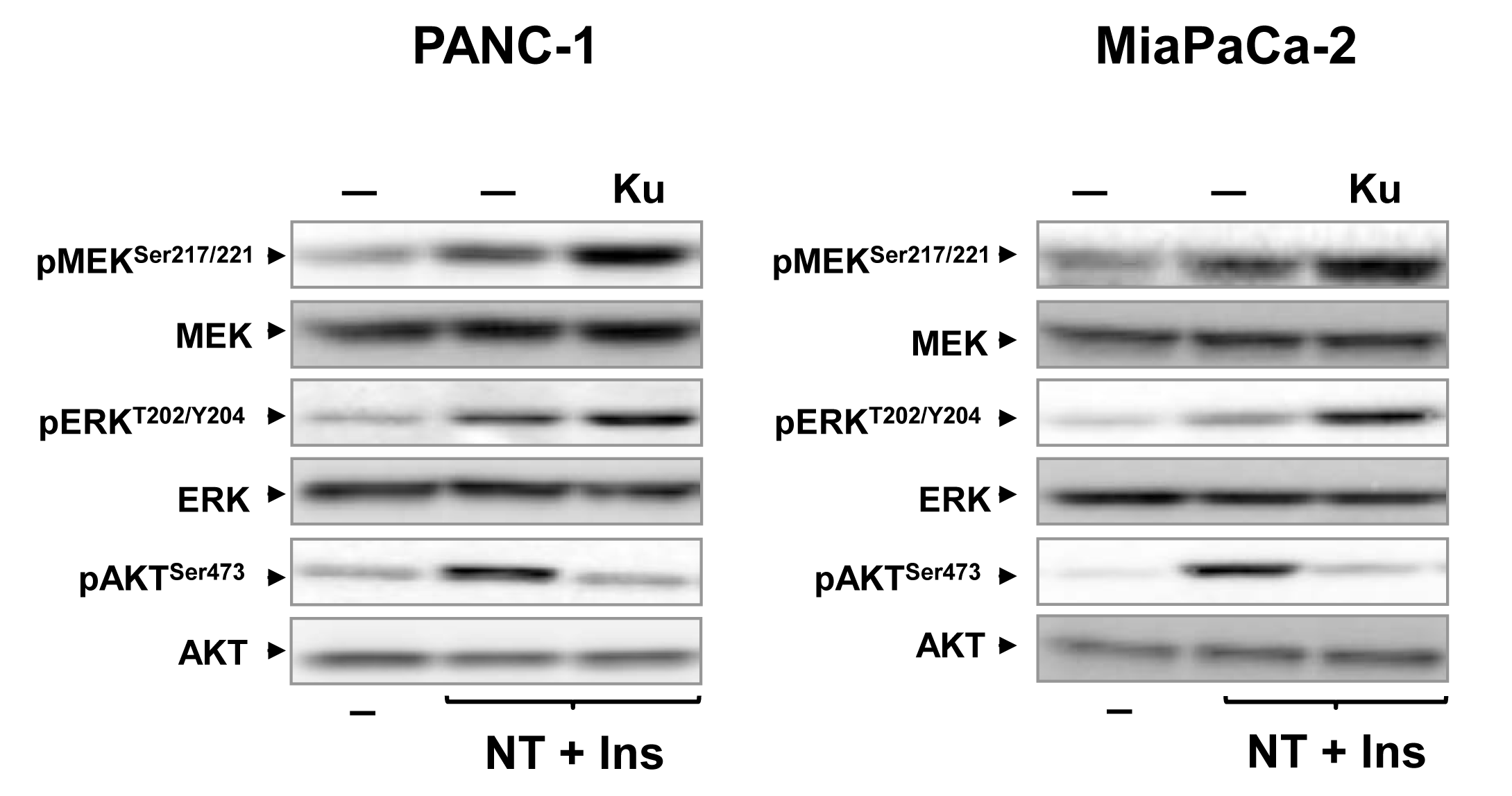

Supplement: Figure S1 — Treatment with KU63794 causes over-activation of MEK and ERK phosphorylation in PANC-1 and MiaPaCa-2 cells. The cultures of PANC-1 and MiaPaCa-2 were incubated in the absence (−) or in the presence of KU63794 (Ku) at 5 mM for 2 h in DMEM containing 5 mM glucose, as indicated. Then, the cells were stimulated with 10 ng/ml insulin and 5 nM neurotensin (NT+Ins) for 2 h and lysed with 2×SDS–PAGE sample buffer. The samples were analyzed by SDS-PAGE and immunoblotting with antibodies that detect the phosphorylated state of MEK at Ser217/221, ERK at Thr202 and Tyr204 and Akt at Ser473 Immunoblotting with total MEK, ERK and Akt was used to verify equal gel loading. (TIF) [file pone.0057289.s001.tif]
