# Supplementary material for: Ex pression of Concern: Different Patterns of Akt and ERK Feedback Activation in Response to Rapamycin, Active-Site mTOR Inhibitors and Metformin in Pancreatic Cancer Cells
Source: PLoS One. 2023 Sep 28;18(9):e0292422. doi: 10.1371/journal.pone.0292422 (PMC10538731; doi:10.1371/journal.pone.0292422)

FIGURE 1

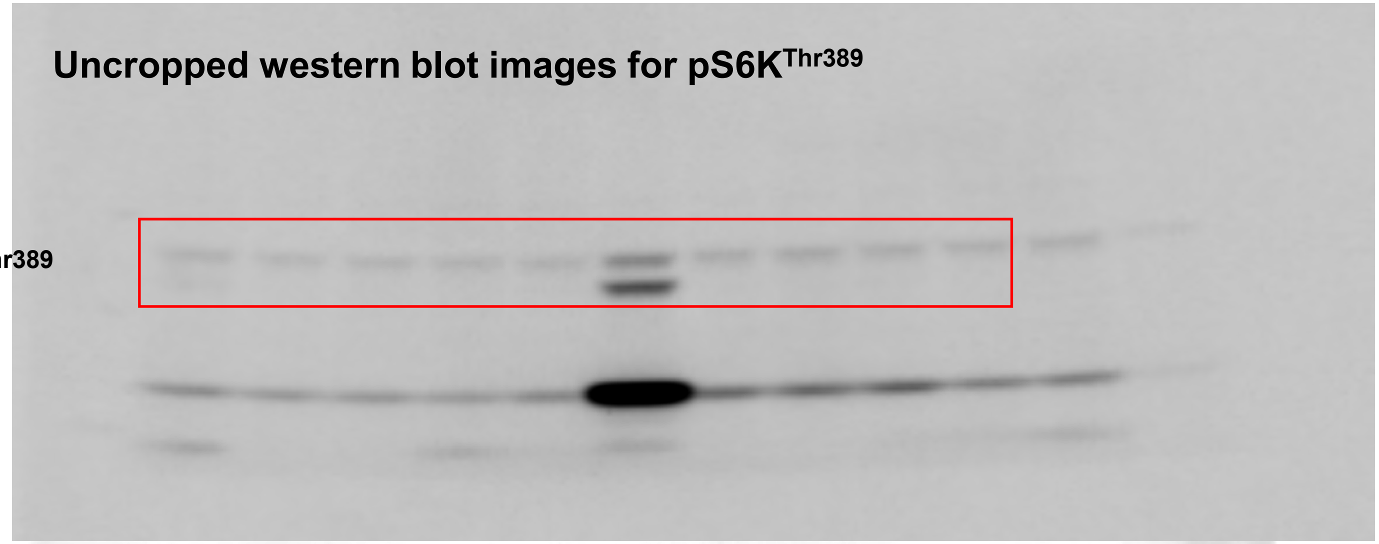

pS6K^{Thr389}

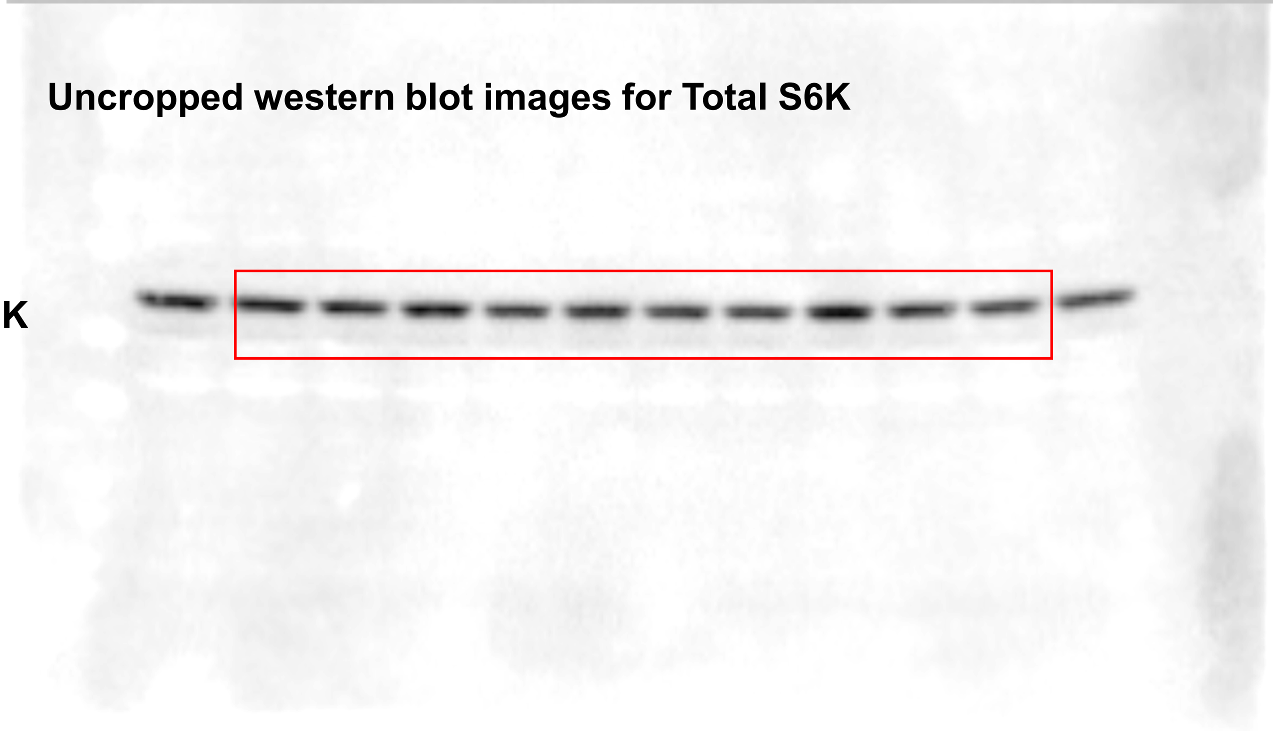

Total S6K

FIGURE 1

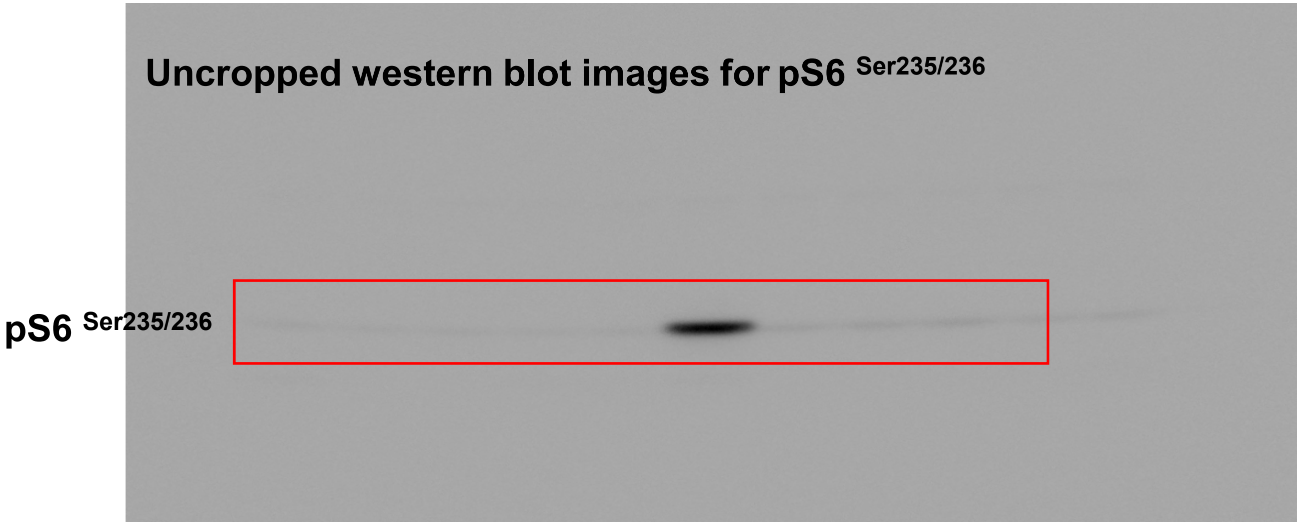
Uncropped western blot images for pS6 Ser235/236
pS6 Ser235/236

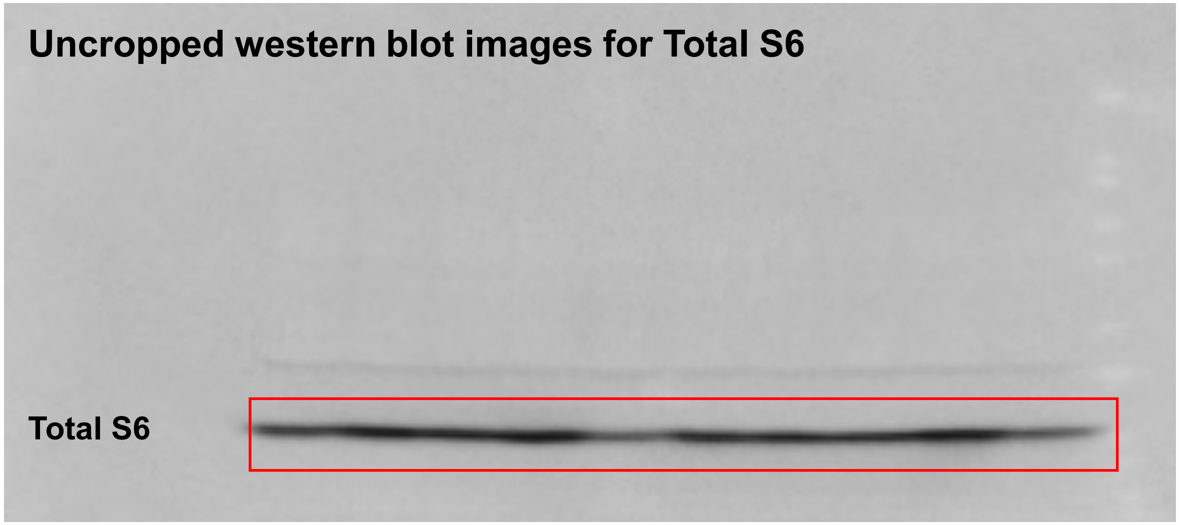
Uncropped western blot images for Total S6
Total S6

FIGURE 1

p4E-BP1$^{Thr37/46}$

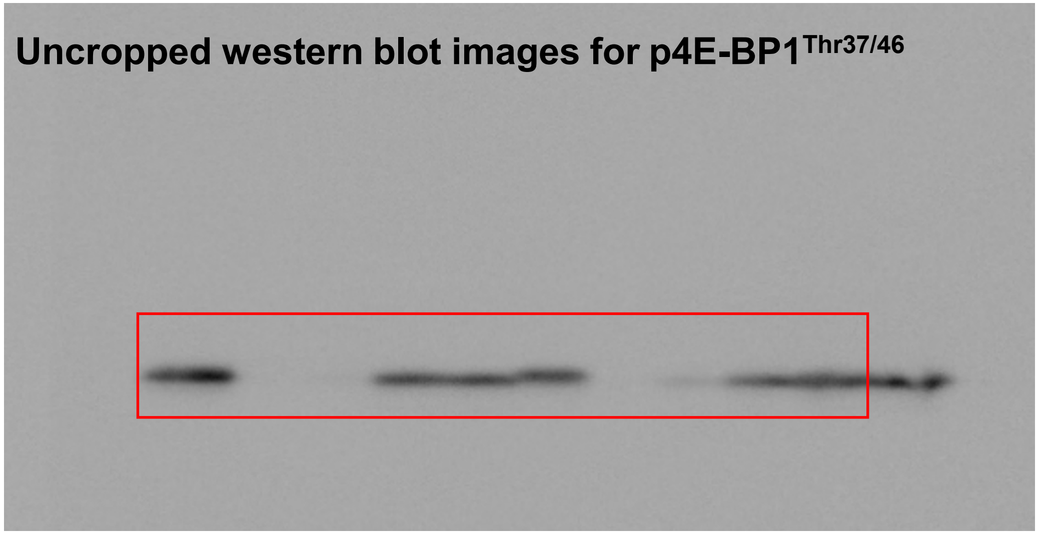

p4E-BP1^{Thr70}

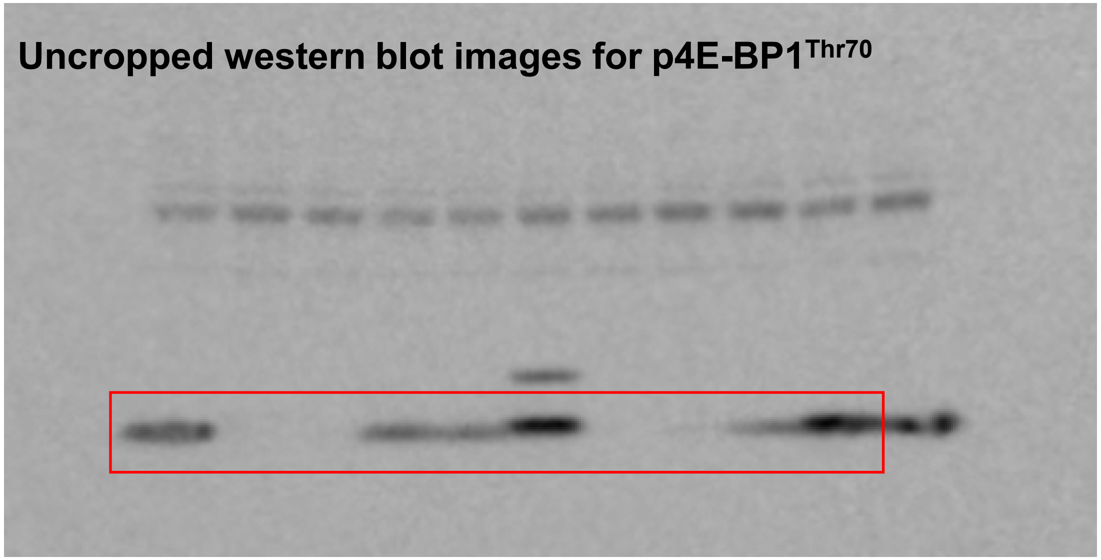

FIGURE 1

Rerun OF Samples

Uncropped western blot images for Total 4E-BP1

Total 4E-BP1

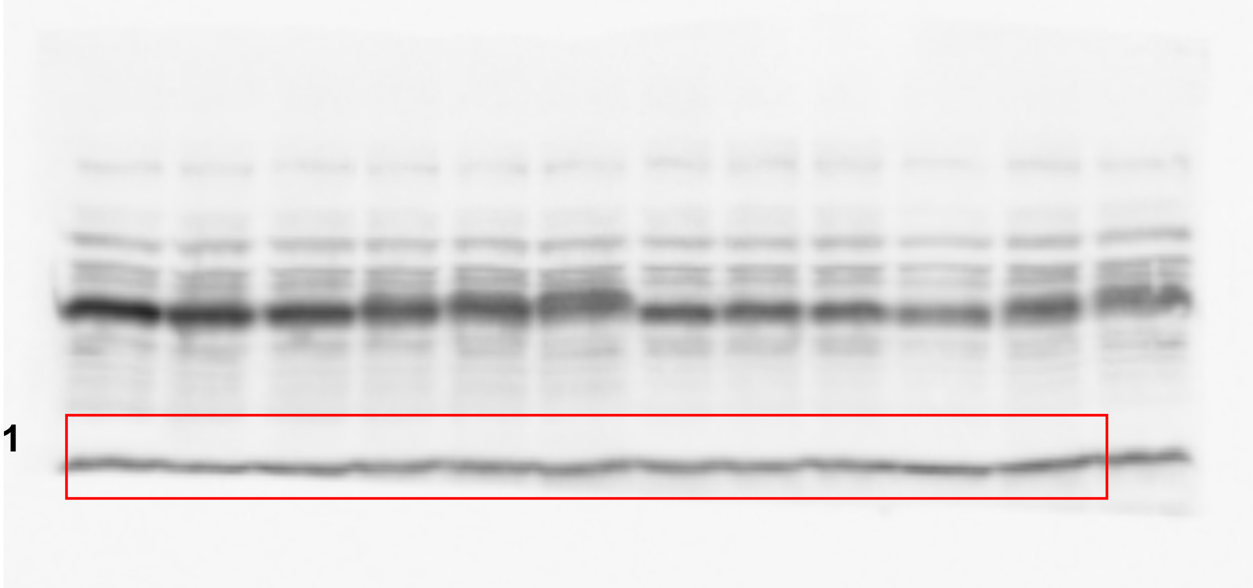

FIGURE 1

Uncropped western blot images for AKT ^{Ser473}

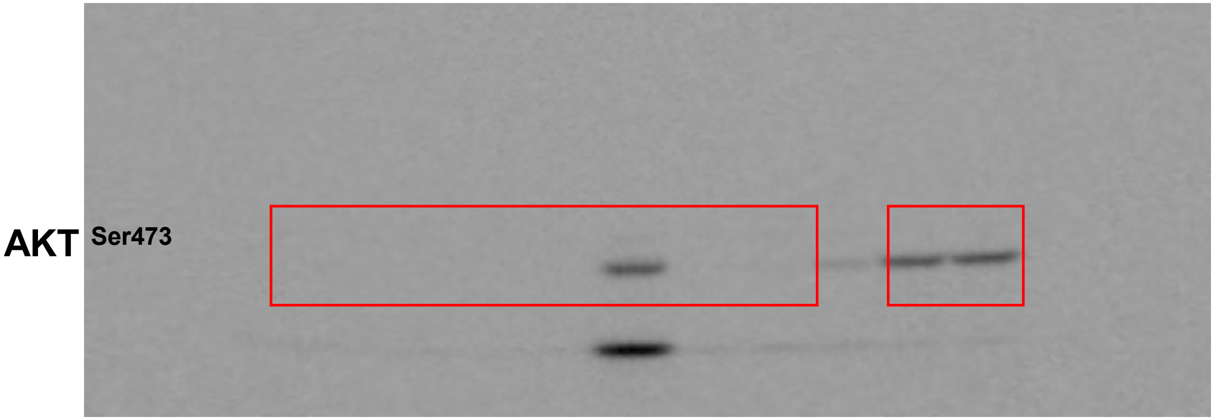

Uncropped western blot images for AKT ^{Thr308}

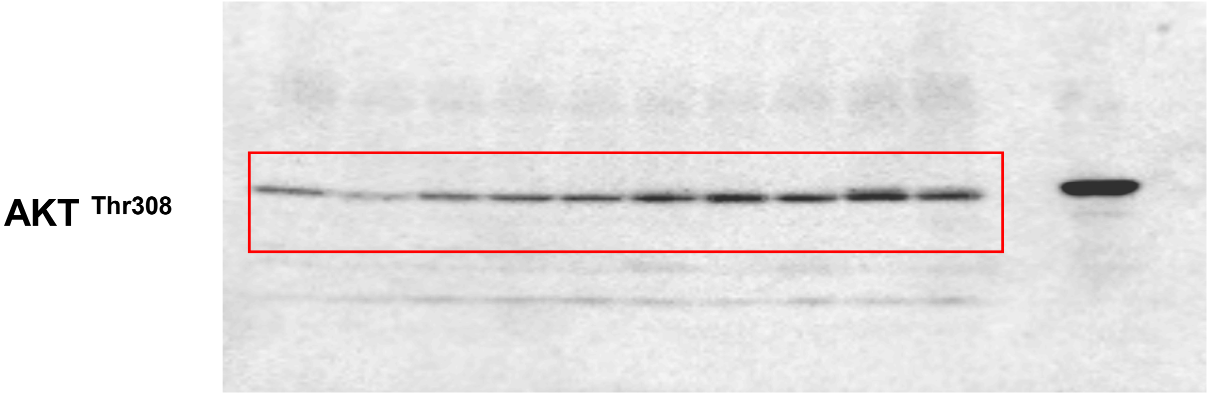

FIGURE 1

Uncropped western blot images for pERK$^{T202/Y204}$

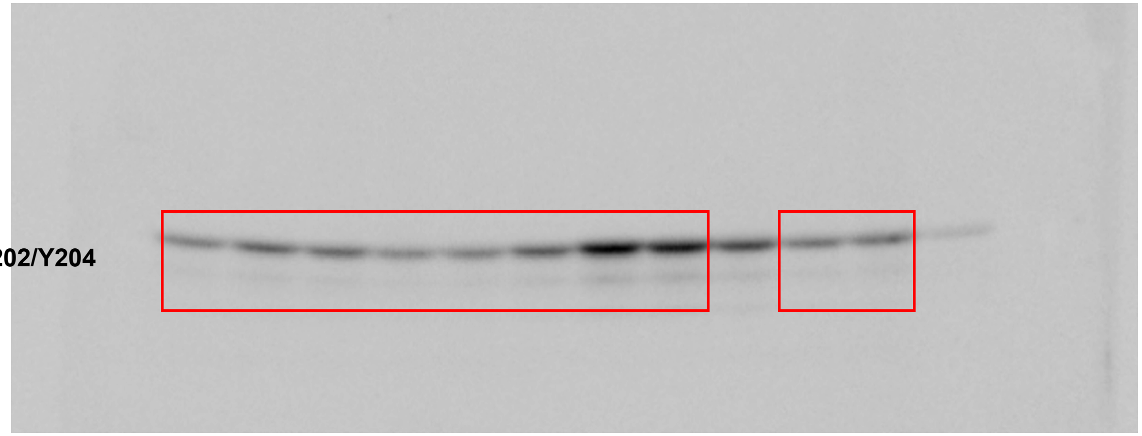

FIGURE 2

Uncropped western blot images for pS6K^{Thr389}

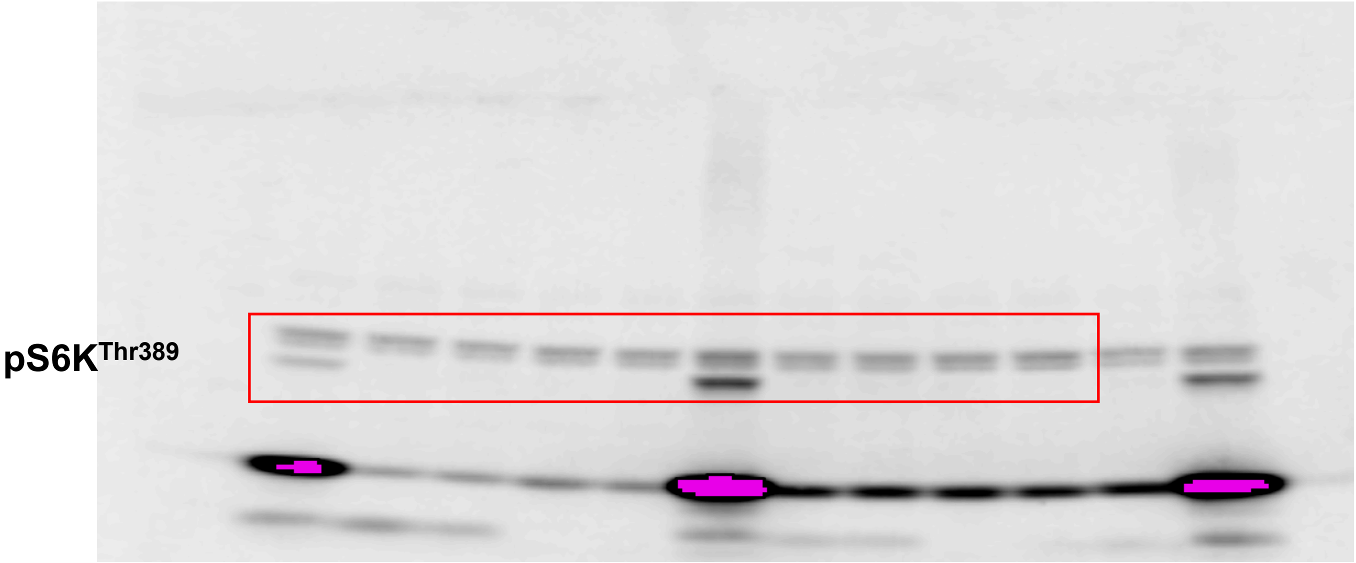

Uncropped western blot images for S6K

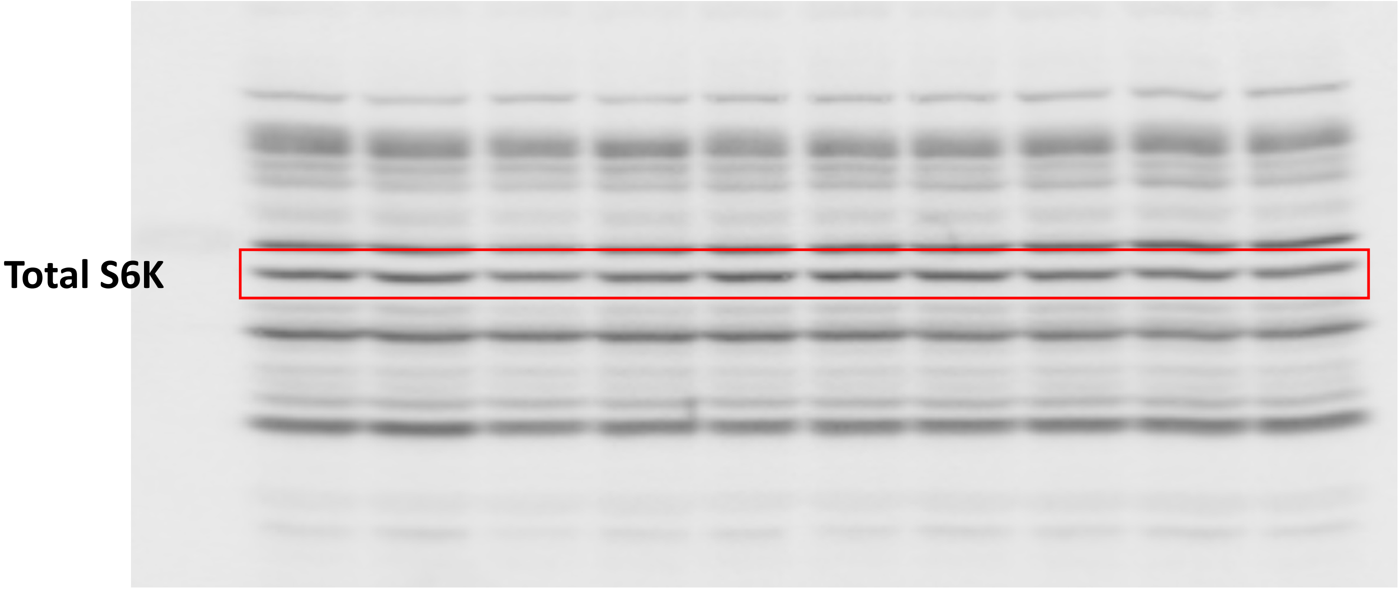

FIGURE 2

Uncropped western blot images for pS6 $^{Ser235/236}$

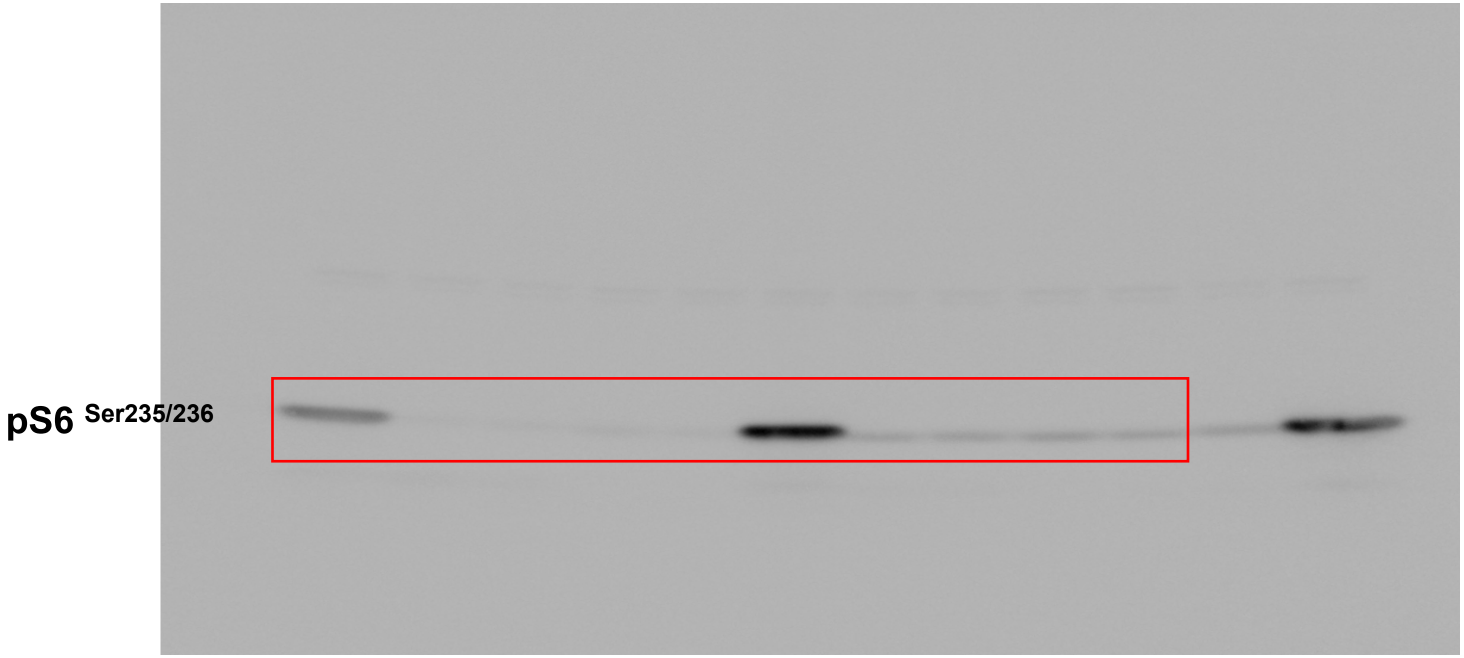

Uncropped western blot images for p4E-BP1$^{Thr37/46}$

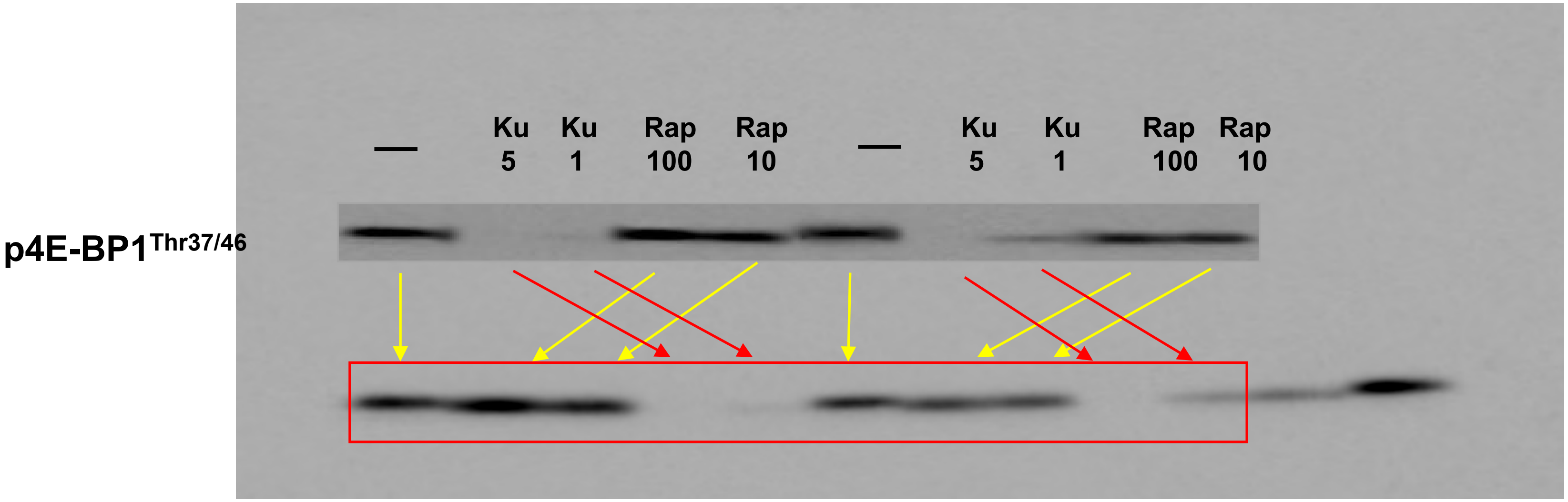

FIGURE 2

Uncropped western blot images for p4E-BP1^{Thr70}

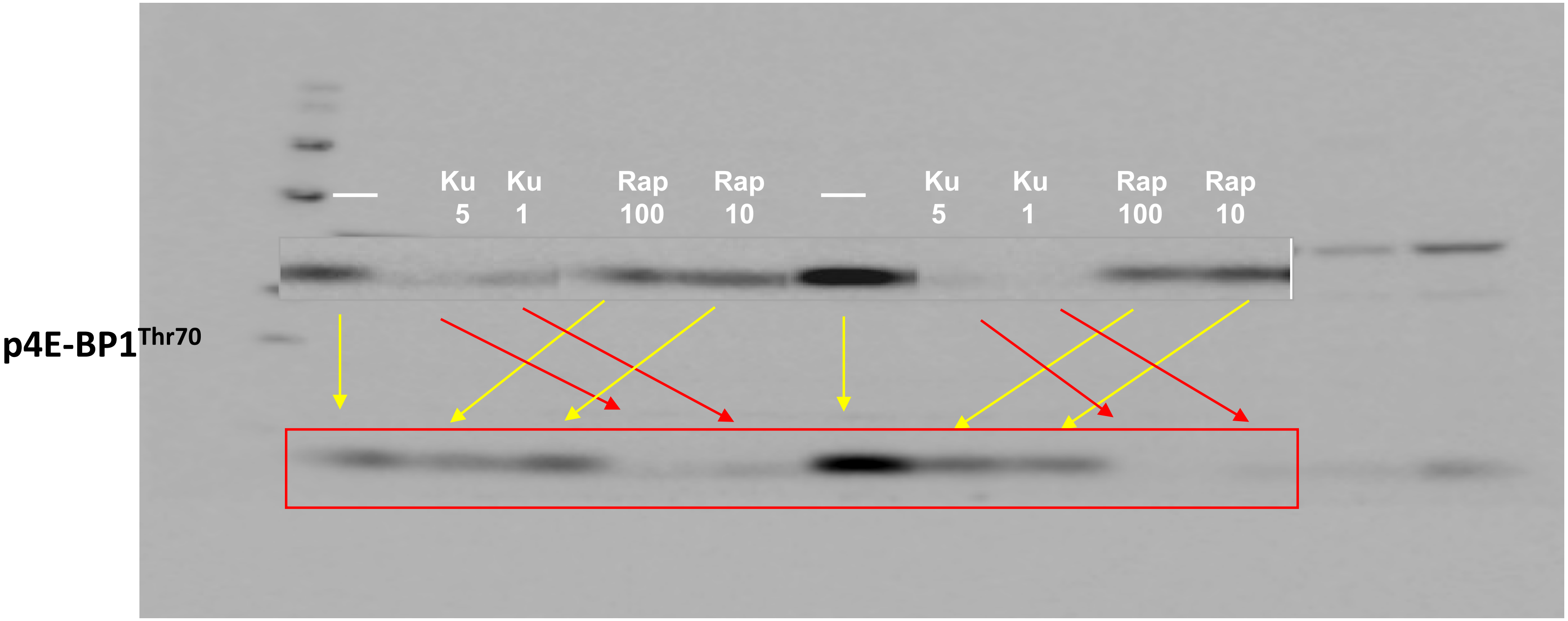

Uncropped western blot images for pAKT^{Thr308}

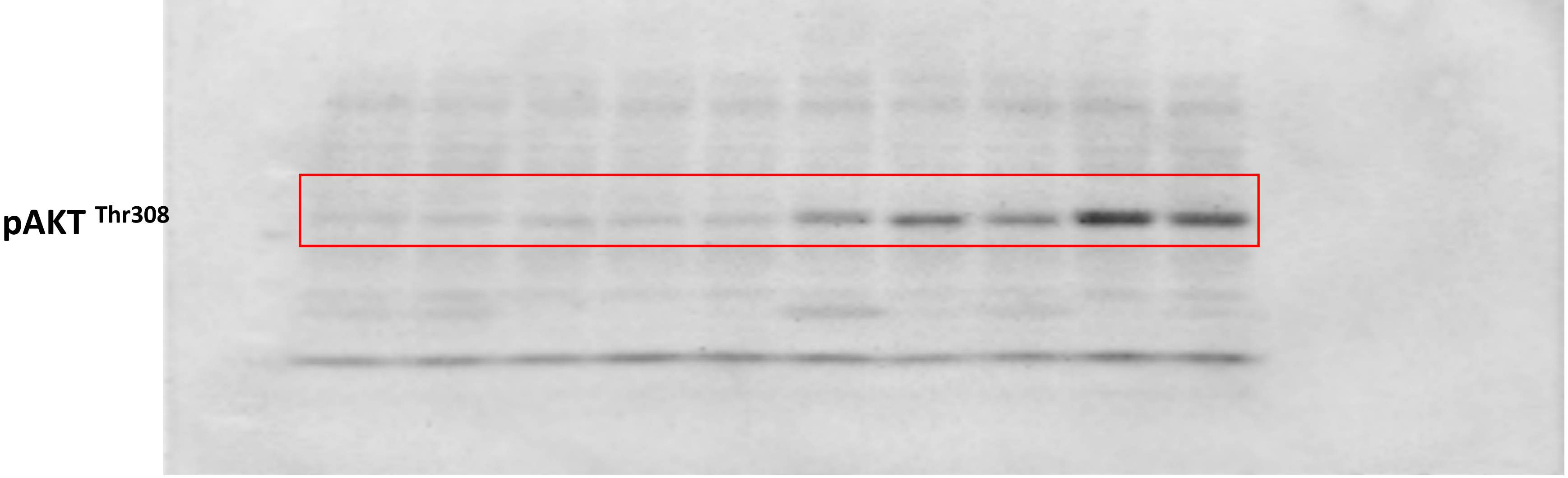

FIGURE 2

Uncropped western blot images for pAKT Ser473

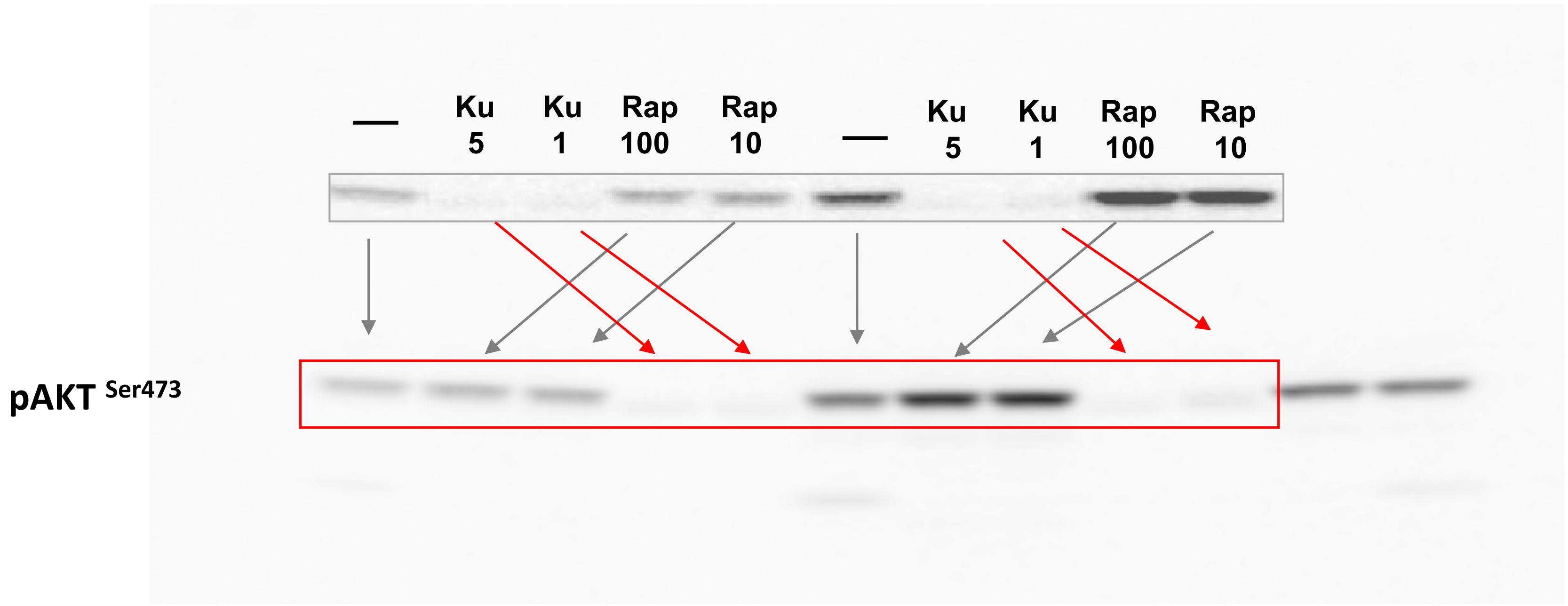

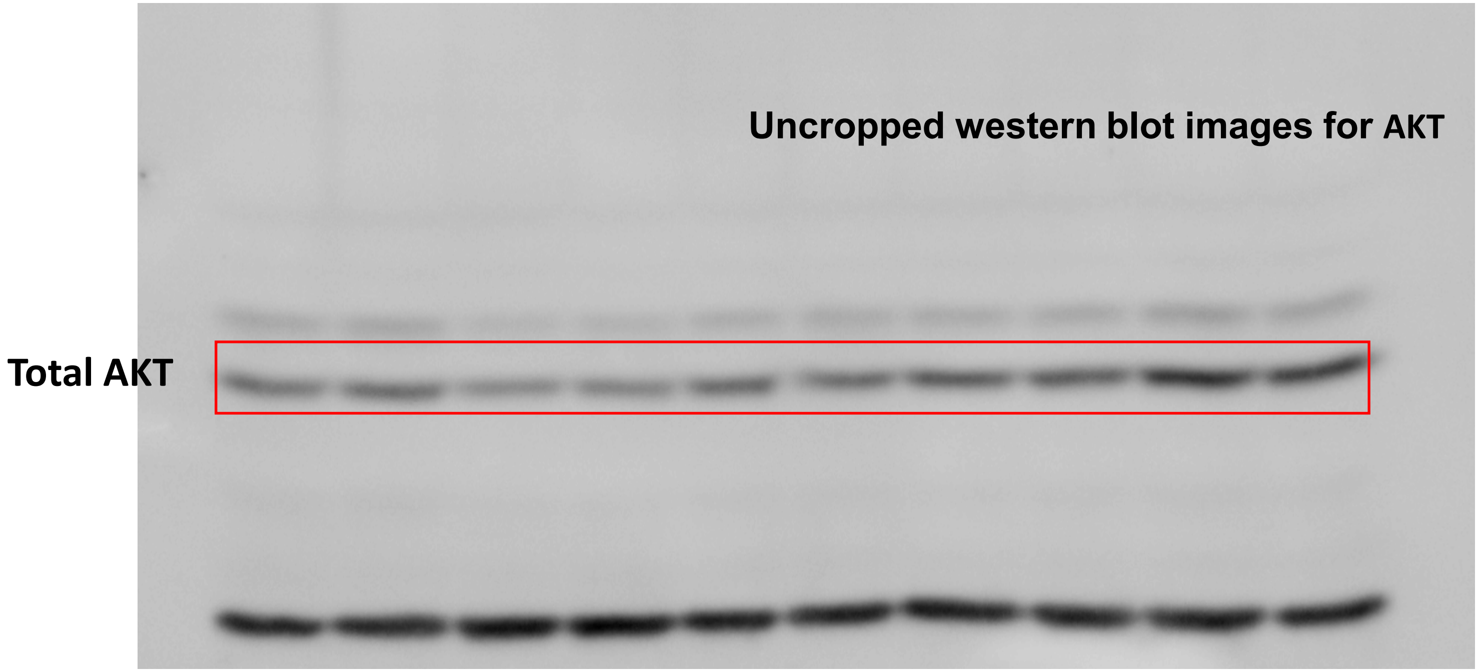

FIGURE 2

Uncropped western blot images for pERK$^{T202/Y204}$

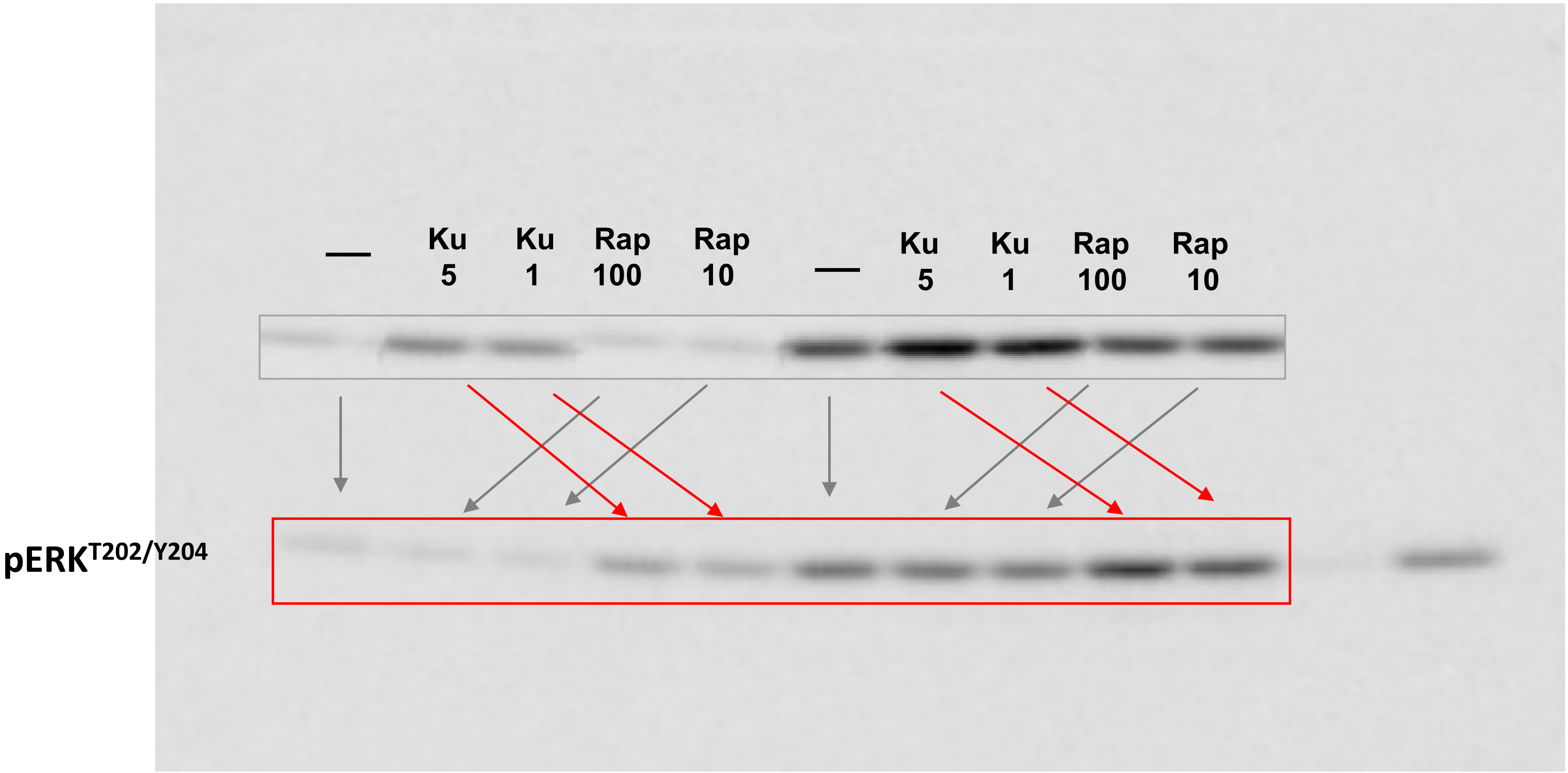

FIGURE 3 PANC-1

Uncropped western blot images for pS6K^{Thr389}

pS6K^{Thr389}

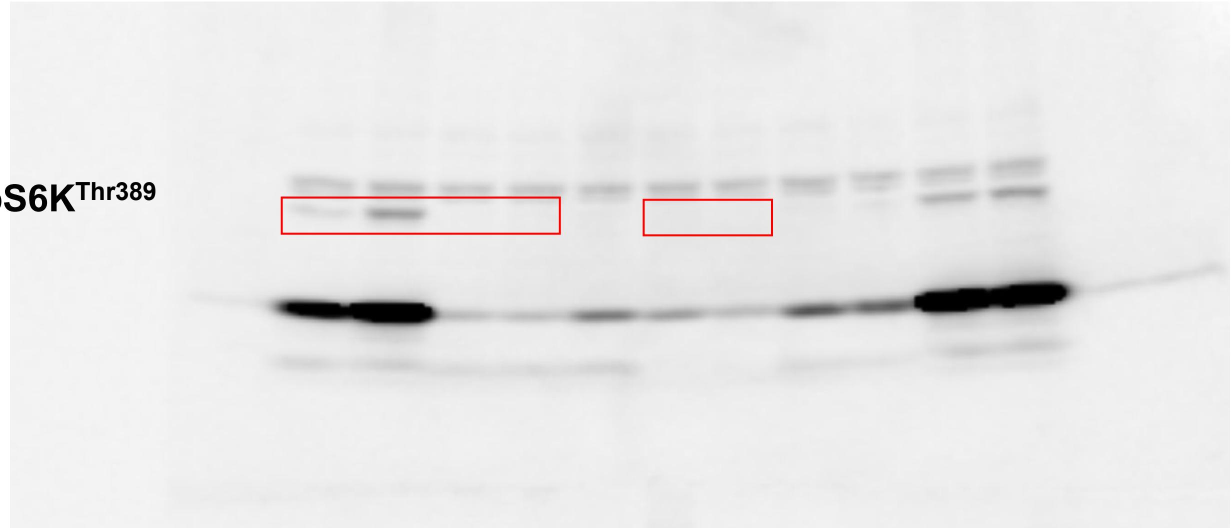

FIGURE 3 PANC-1

Uncropped western blot images for pS6$^{Ser235/236}$

pS6$^{Ser235/236}$

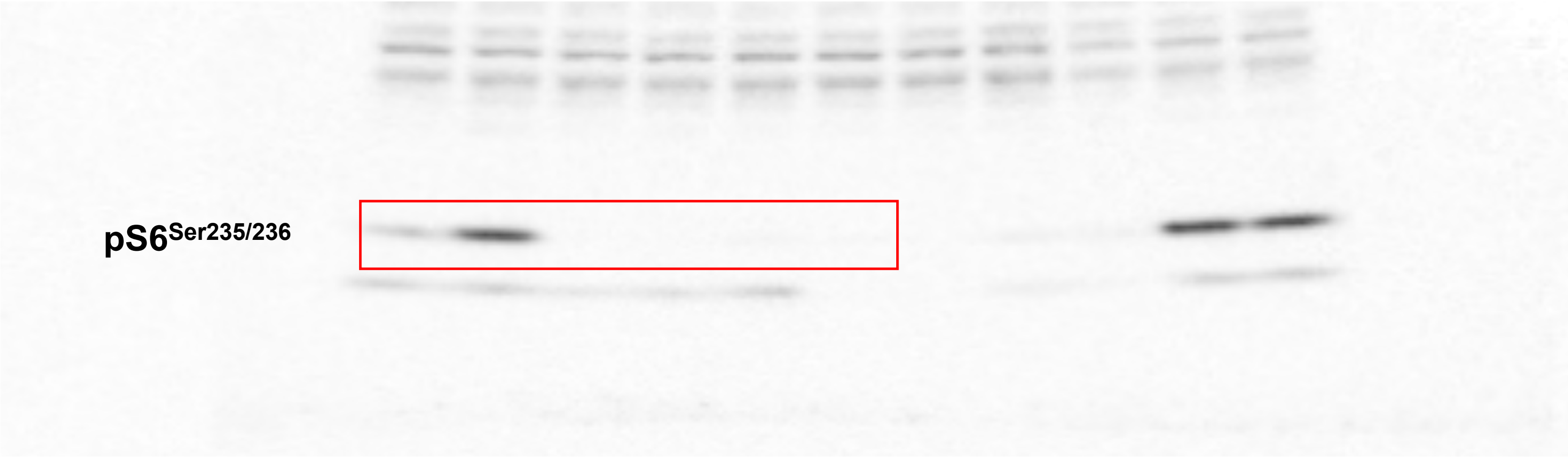

FIGURE 3 PANC-1

Uncropped western blot images for S6

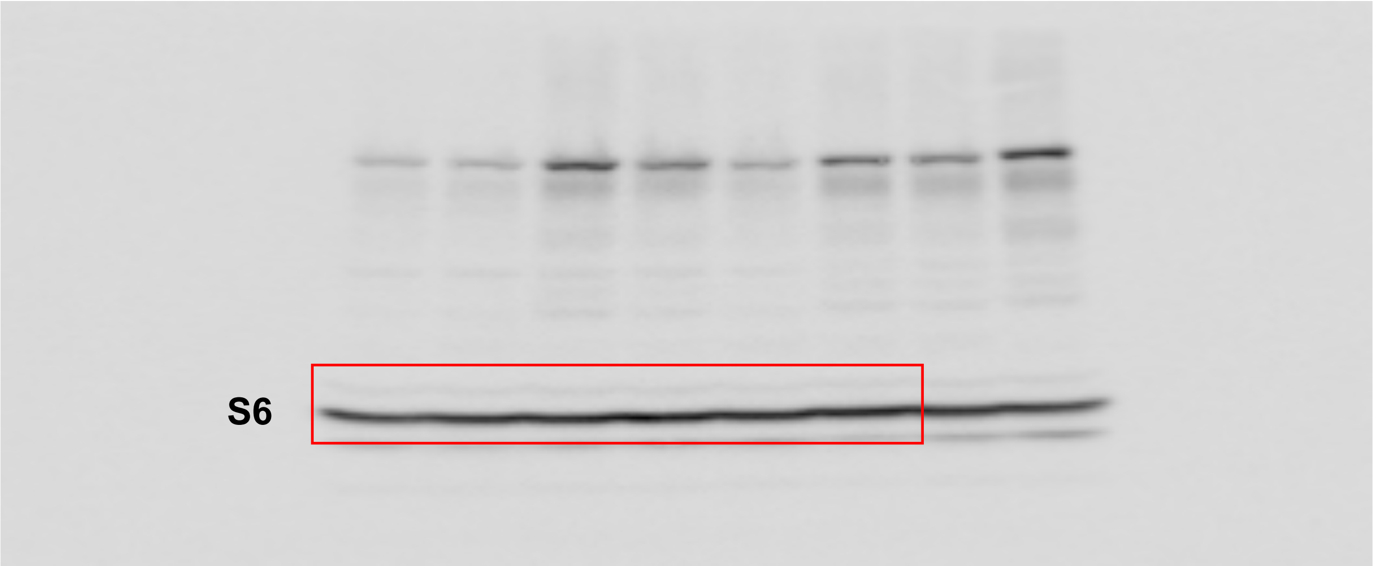

FIGURE 3 PANC-1

Uncropped western blot images for pAKT Thr308

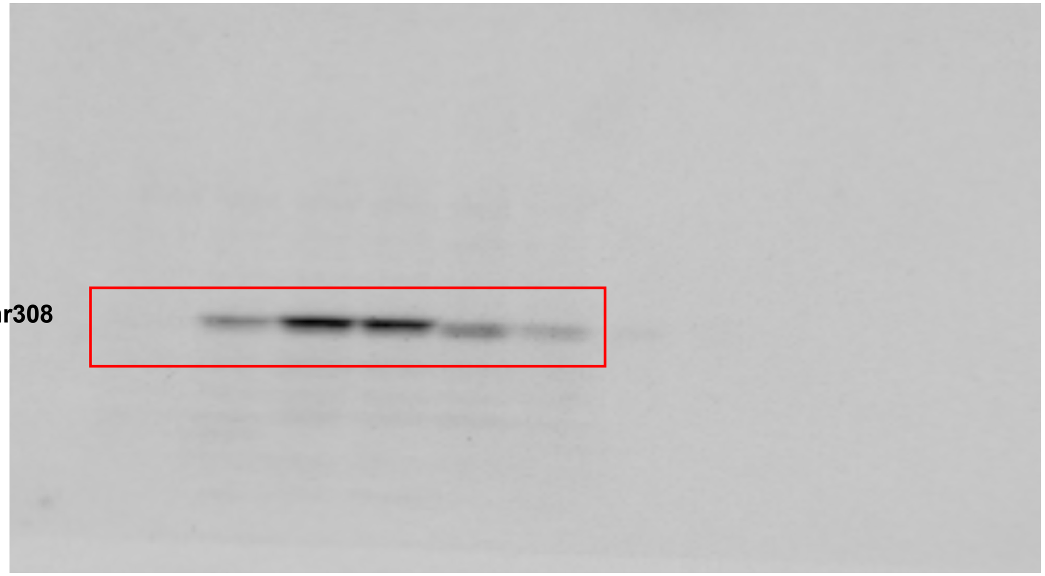

FIGURE 3 PANC-1

Uncropped western blot images for pAKT Ser473

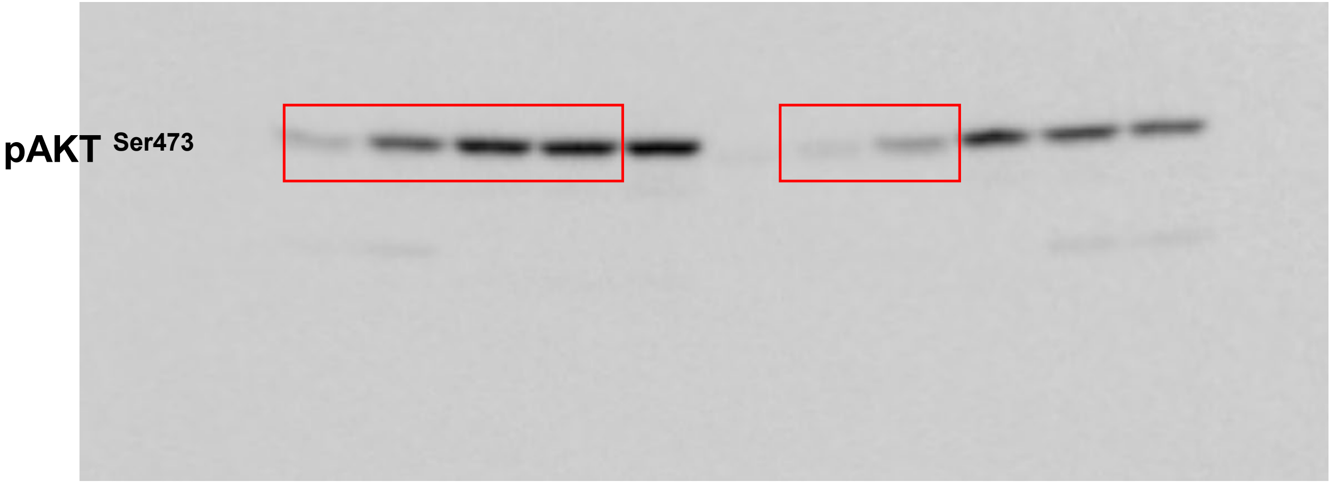

FIGURE 3 PANC-1

Uncropped western blot images for AKT

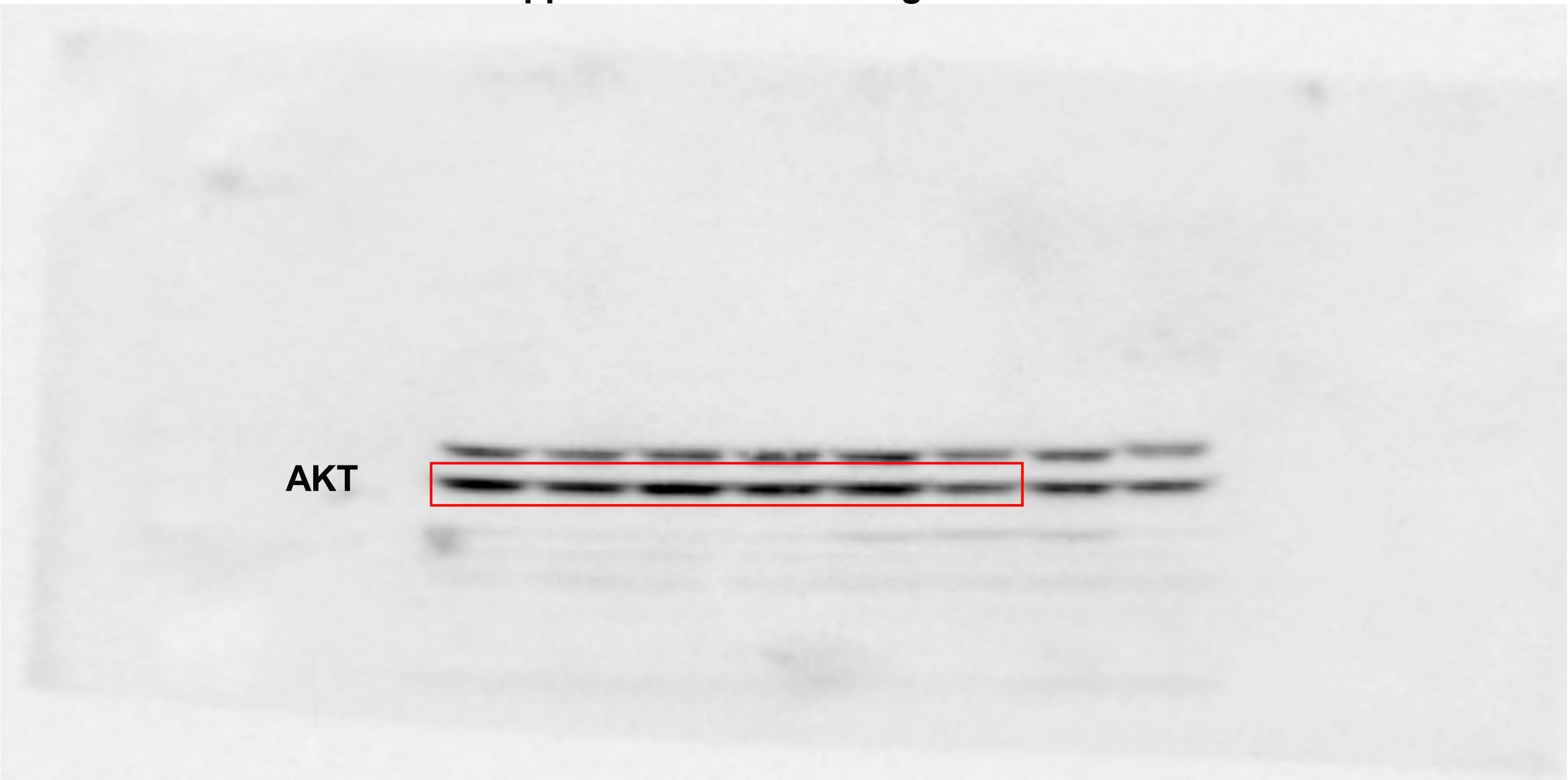

FIGURE 3 PANC-1

Uncropped western blot images for $pERK^{T202Y/204}$

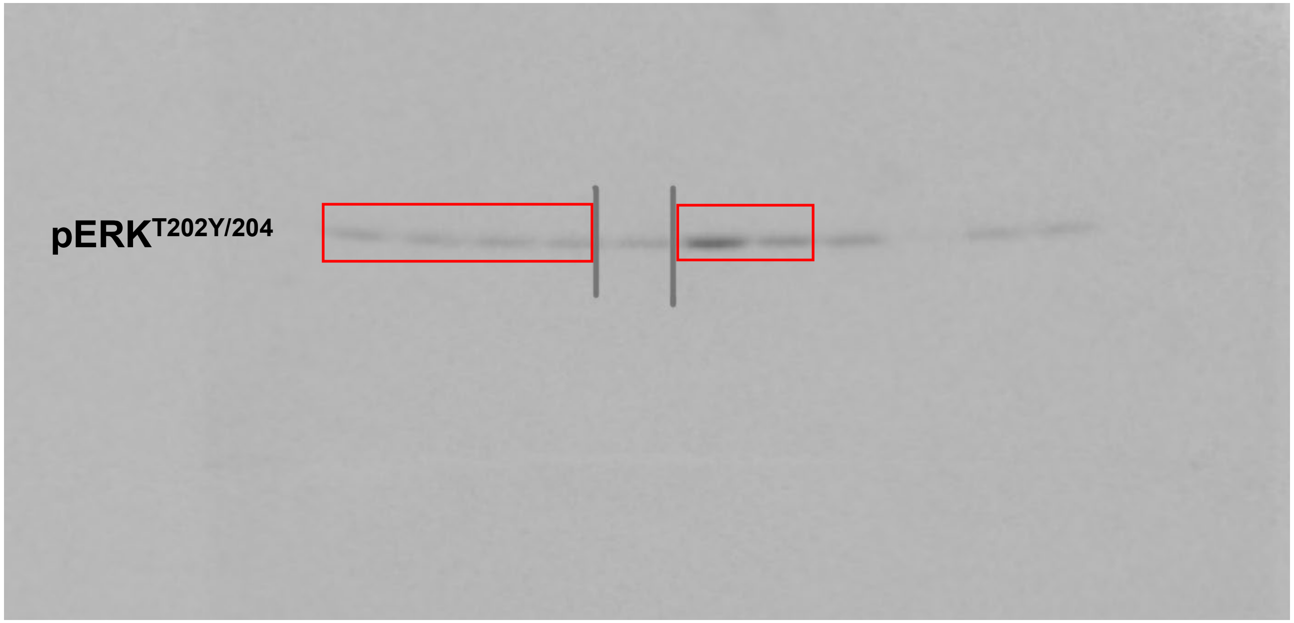

FIGURE 3 PANC-1

Uncropped western blot images for Total ERK

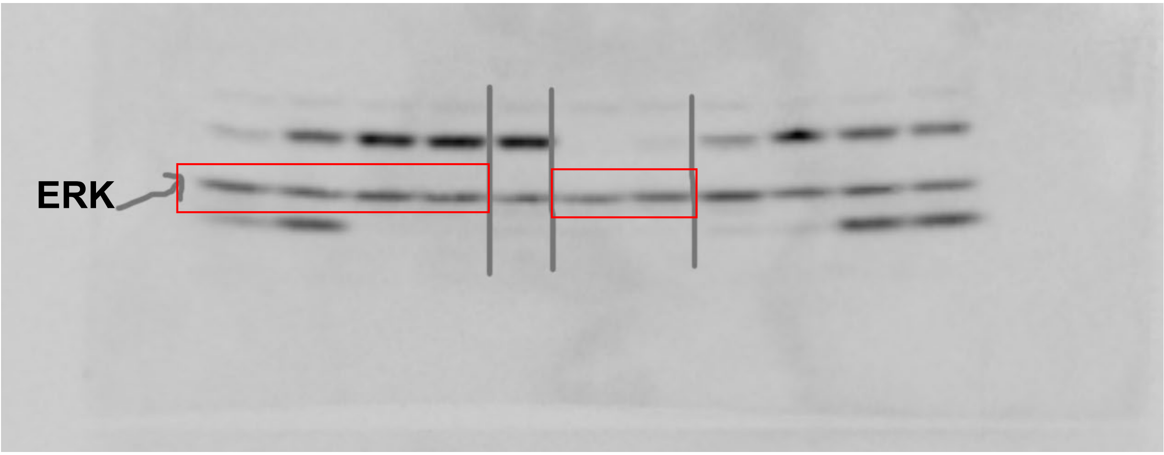

FIGURE 3 Mia PaCa-2

Uncropped western blot images for pS6K^{Thr389}

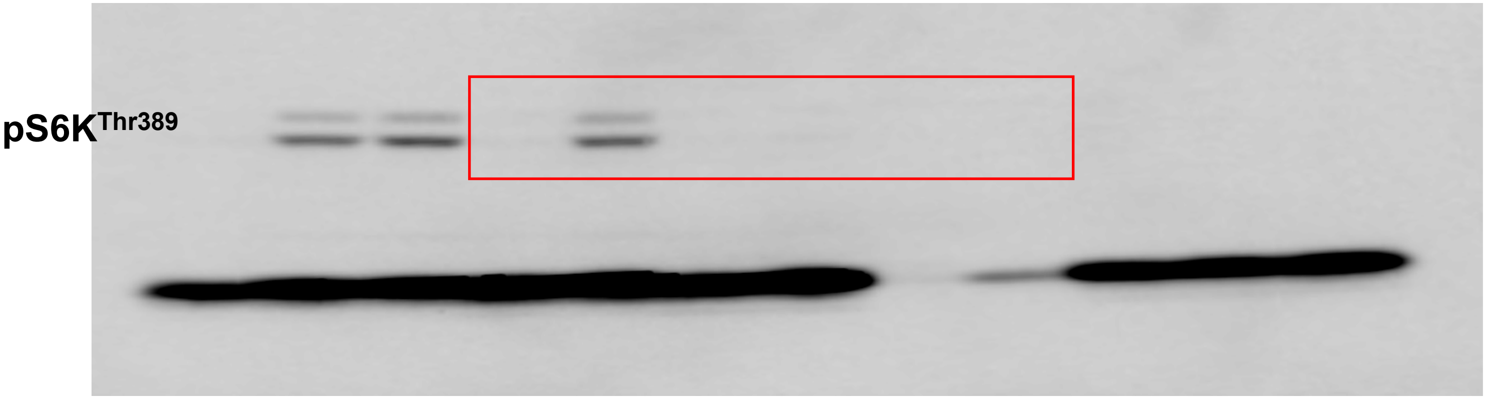

FIGURE 3 Mia PaCa-2

Uncropped western blot images for S6K

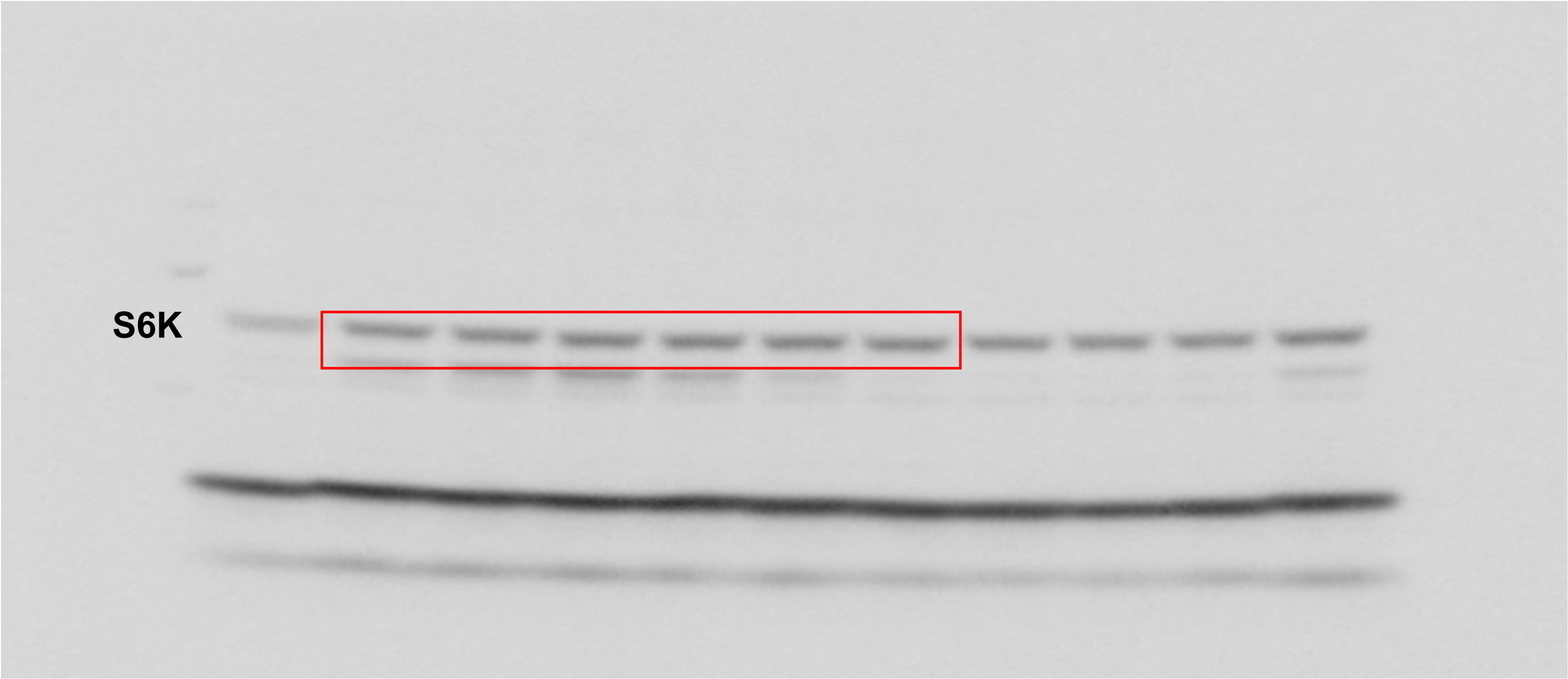

FIGURE 3 Mia PaCa-2

Uncropped western blot images for pS6$^{Ser235/236}$

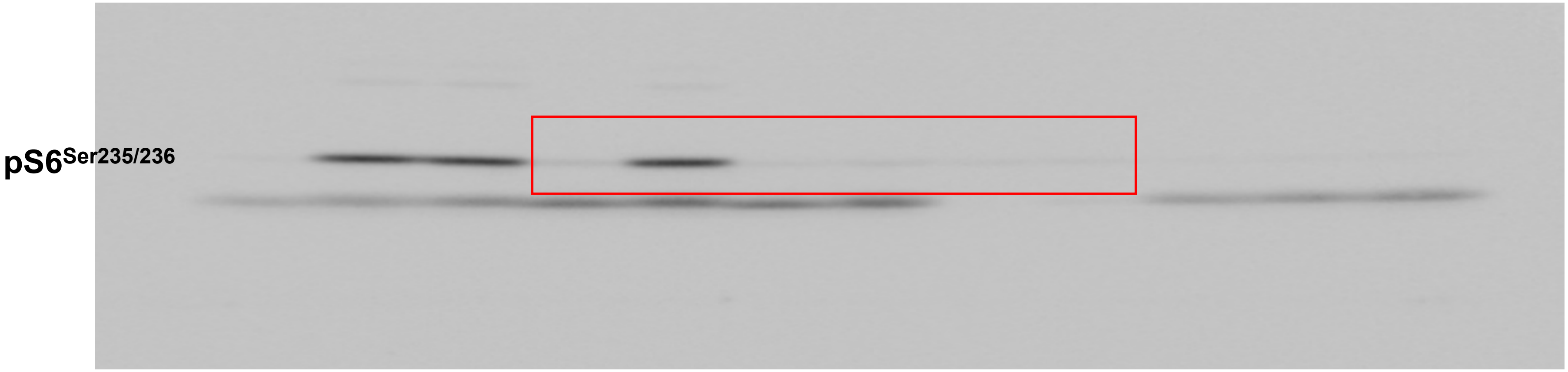

FIGURE 3 Mia PaCa-2

Uncropped western blot images for Total S6

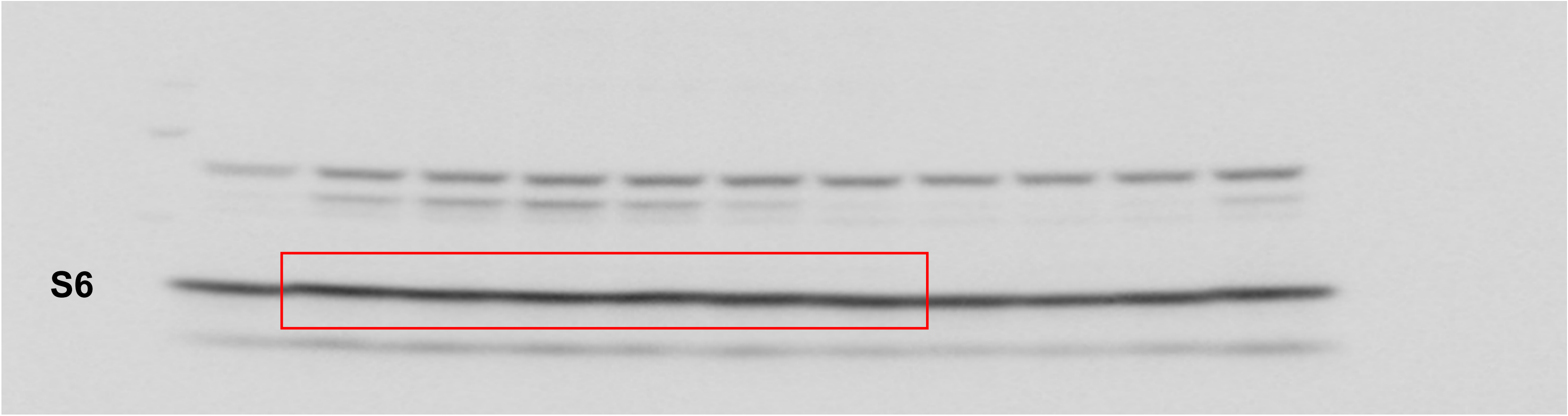

FIGURE 3 Mia PaCa-2

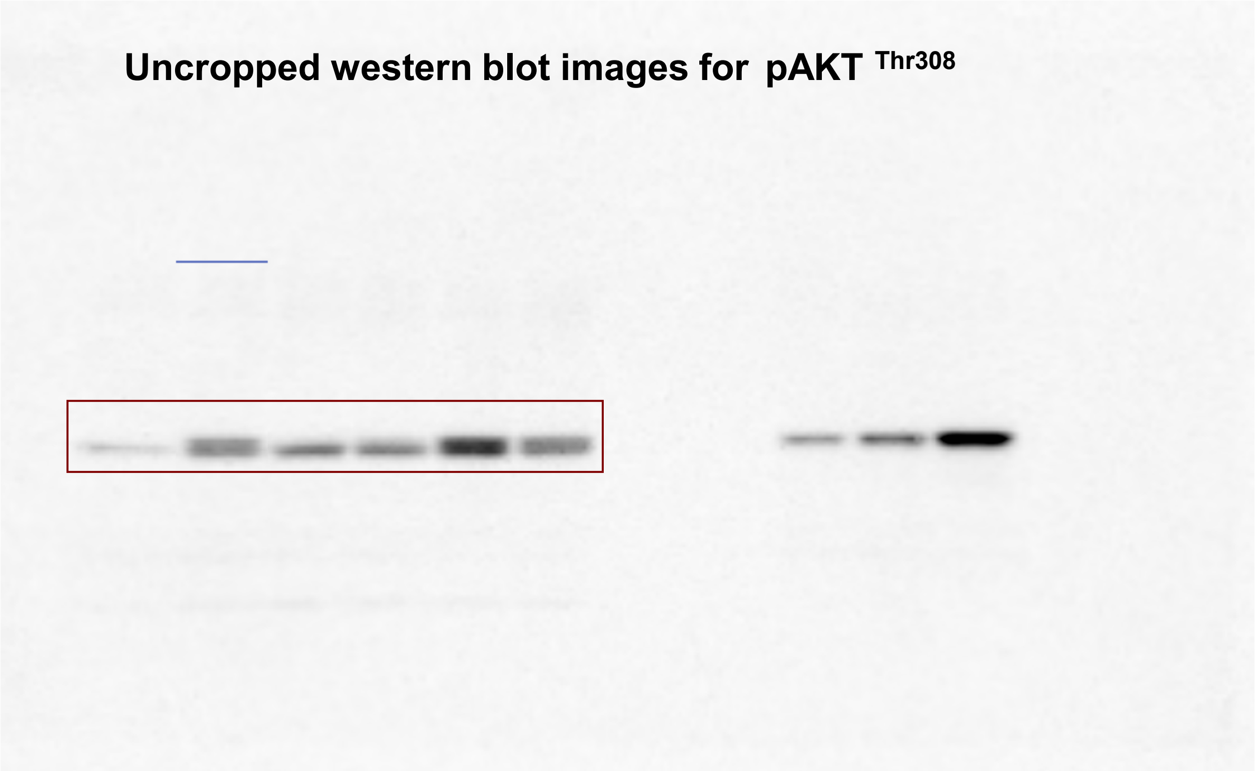

pAKT ^{Thr308}

FIGURE 3 Mia PaCa-2

Uncropped western blot images for pAKT Ser473

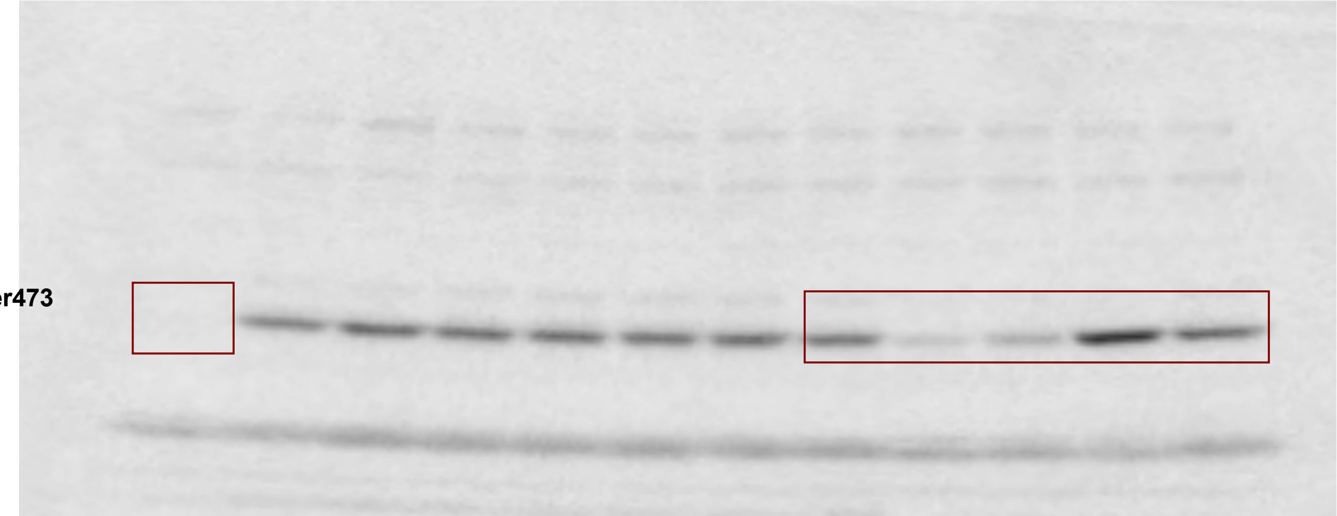

FIGURE 3 Mia PaCa-2

Uncropped western blot images for Total AKT

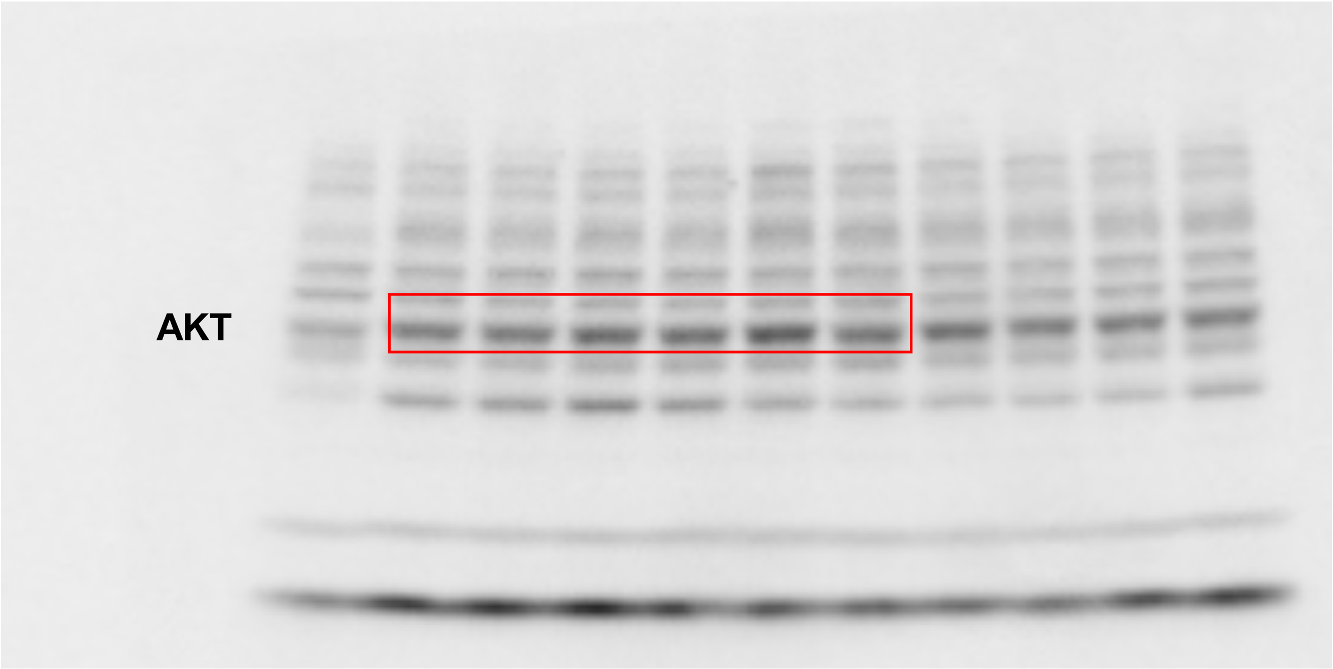

FIGURE 3 Mia PaCa-2

Uncropped western blot images for $pERK^{T202/Y204}$

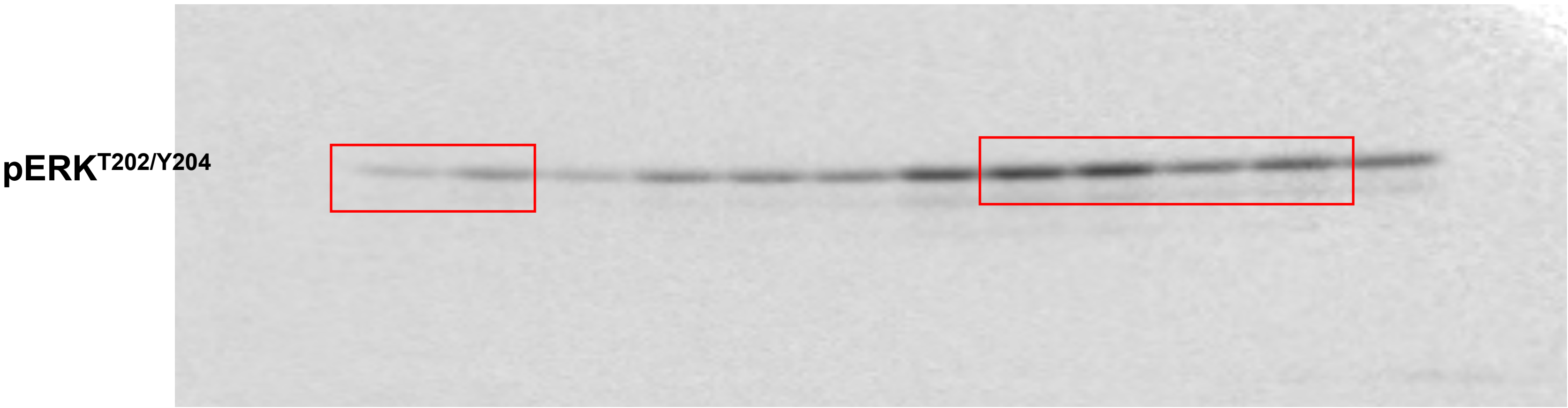

FIGURE 4 PANEL A

Uncropped western blot images for pS6K^{Thr389}

pS6K^{Thr389}

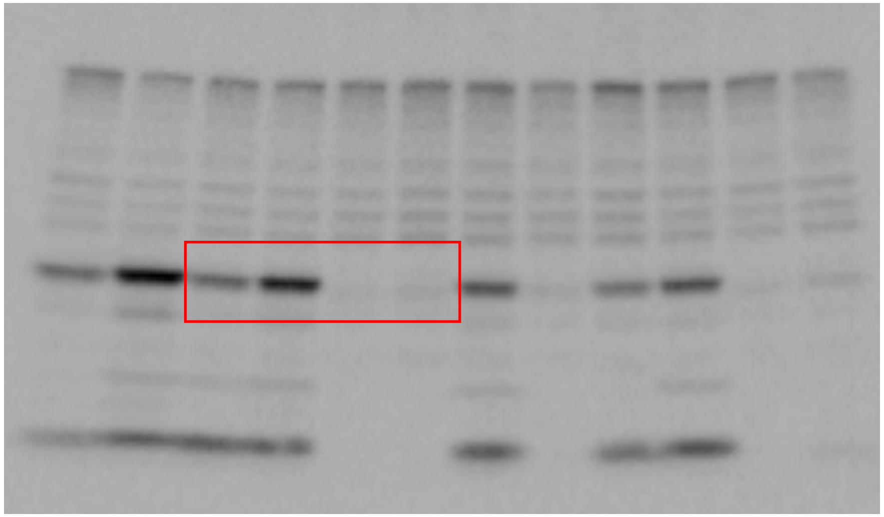

FIGURE 4 PANEL A

Uncropped western blot images for pS6Ser$^{235/236}$

pS6Ser$^{235/236}$

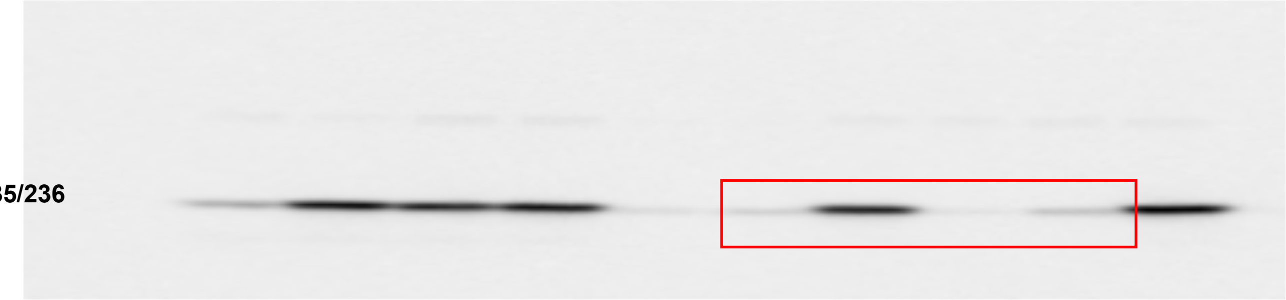

FIGURE 4 PANEL A

Uncropped western blot images for p4E-BP1$^{Thr37/46}$

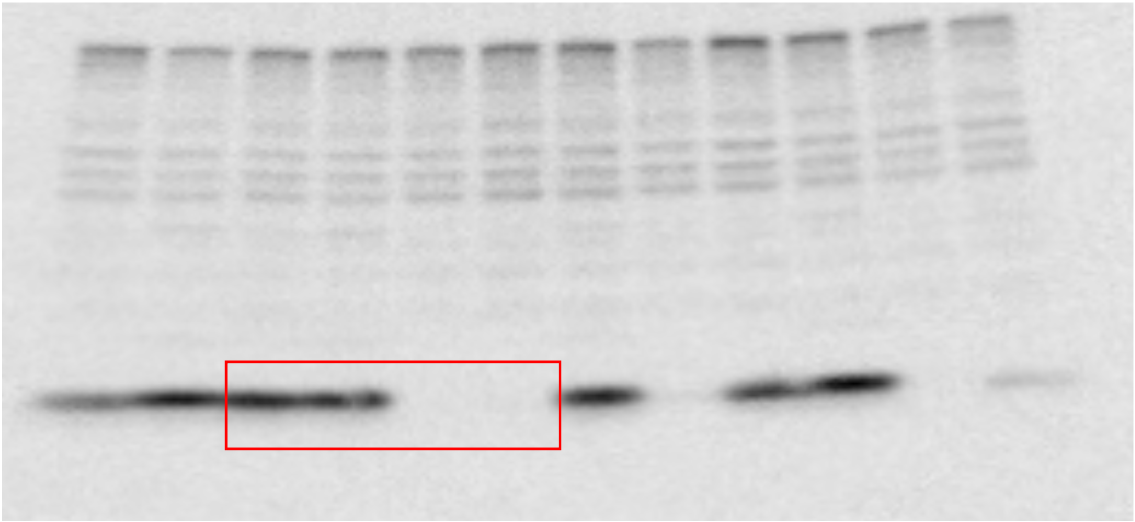

FIGURE 4 PANEL A

Uncropped western blot images for 4E-BP1

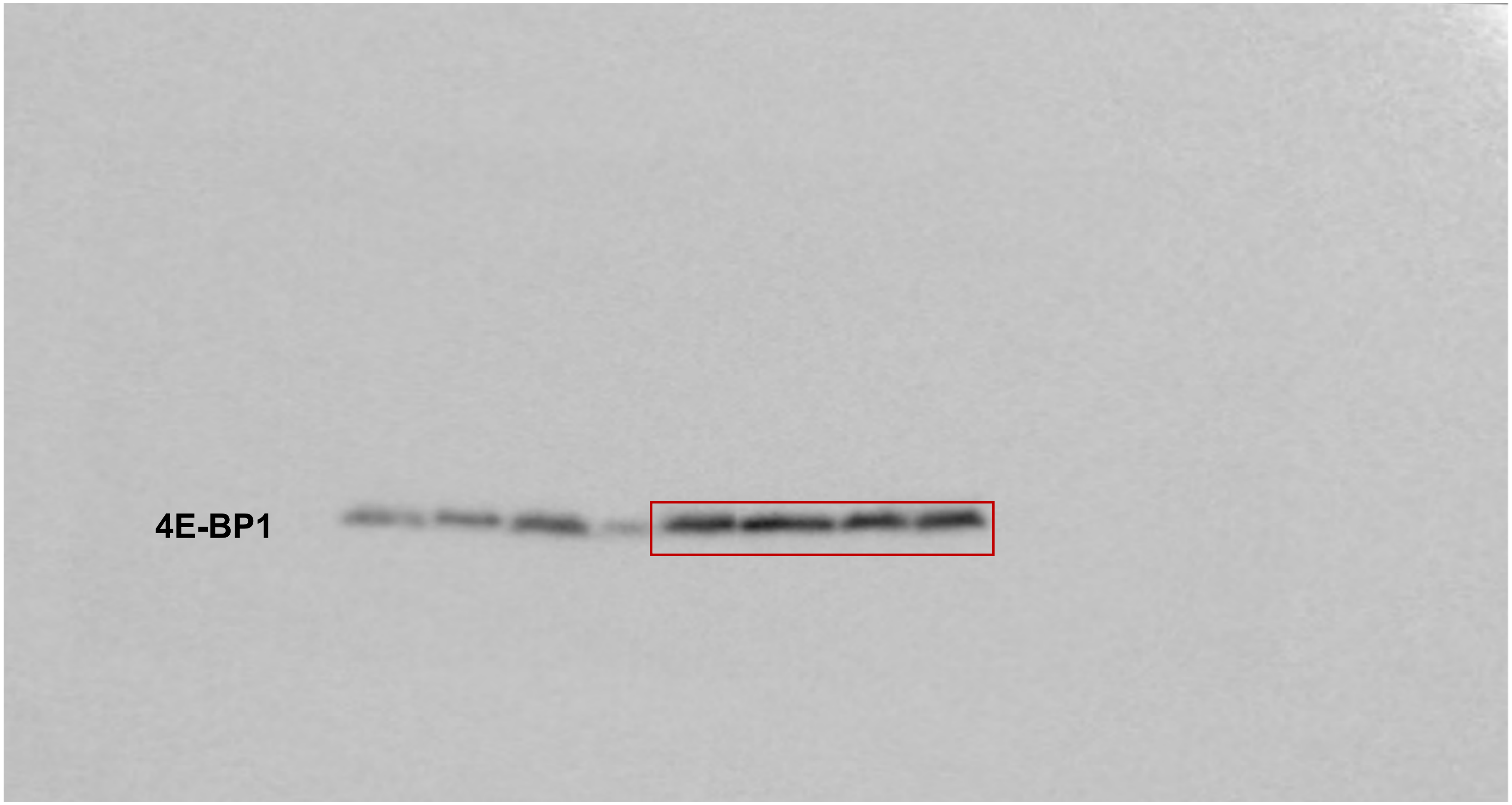

FIGURE 4 PANEL A

Uncropped western blot images for pAKT^{Ser473}

pAKT^{Ser473}

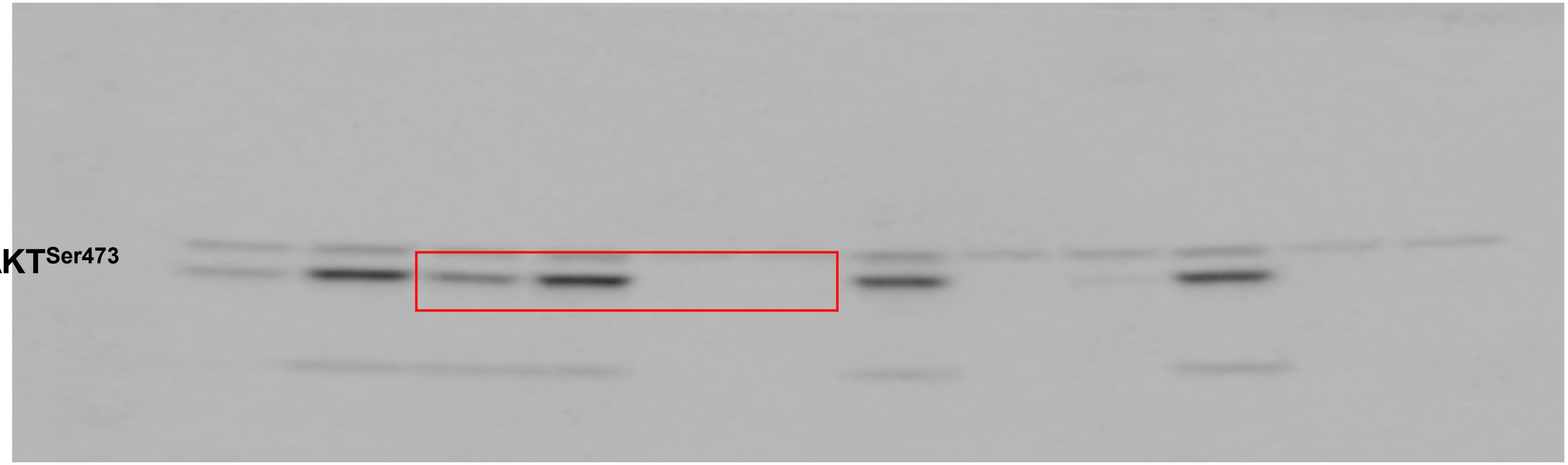

FIGURE 4 PANEL A

Uncropped western blot images for Total AKT

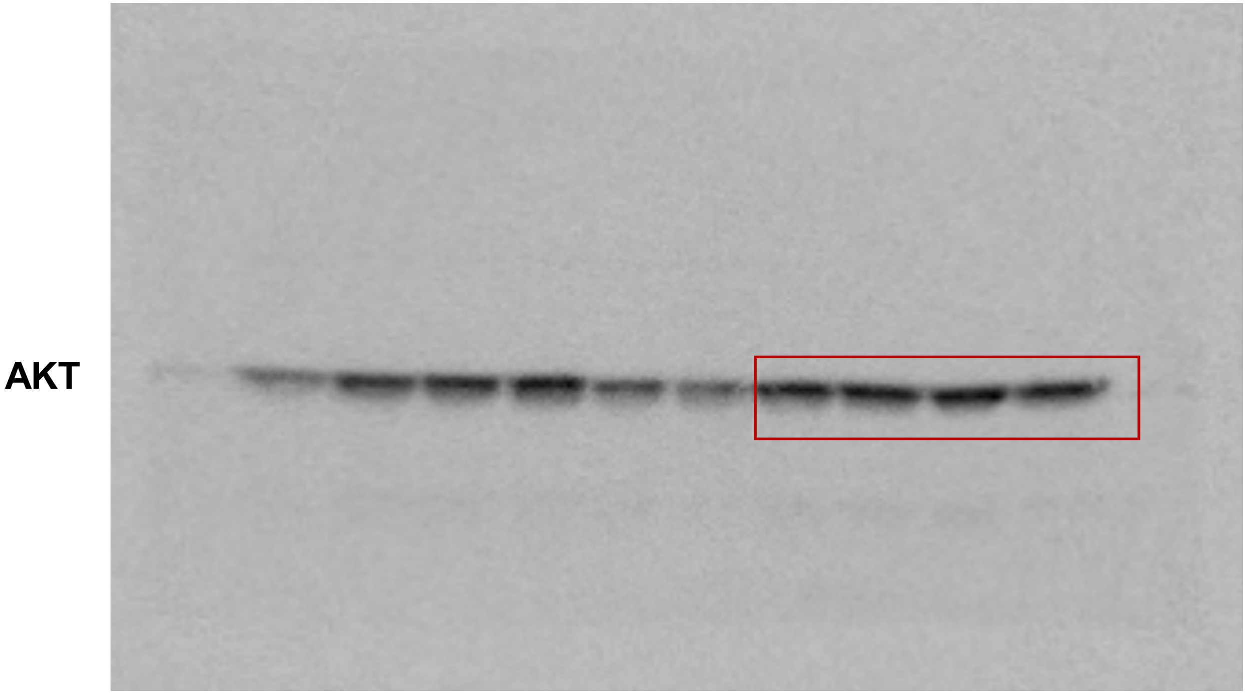

FIGURE 4 PANEL A

Uncropped western blot images for $pERK^{T202/Y204}$

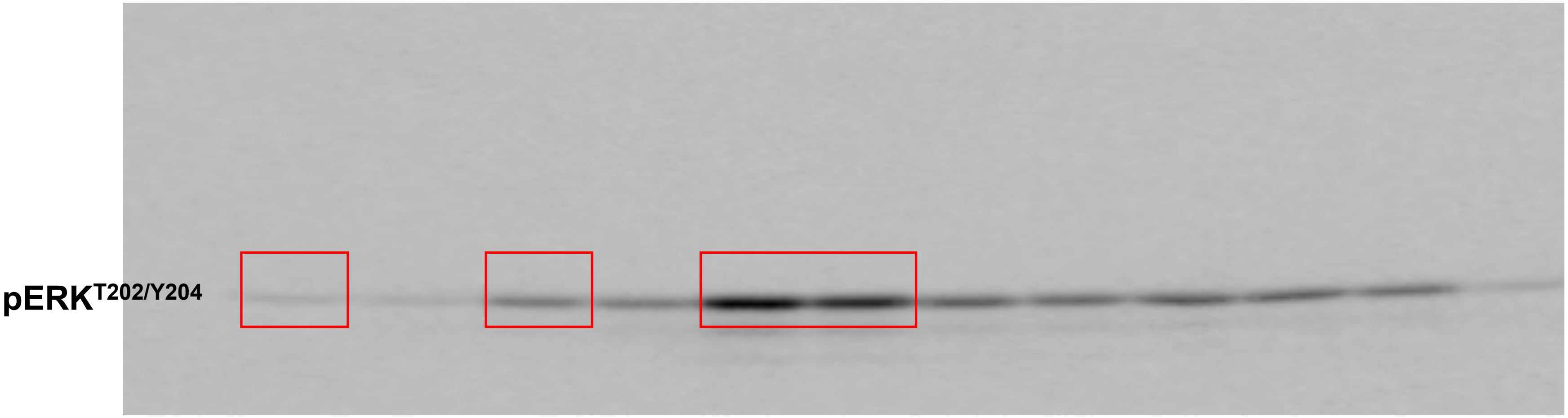

FIGURE 4 PANEL A

Uncropped western blot images for Total ERK

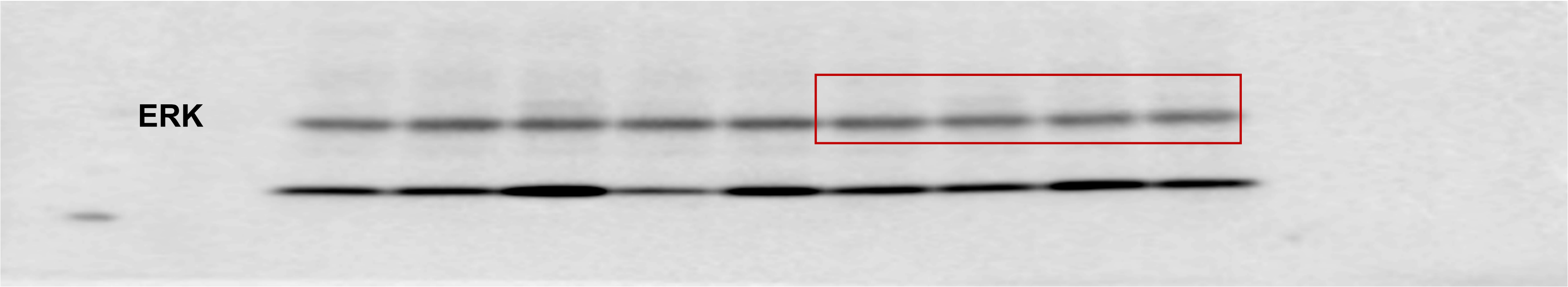

FIGURE 4 PANEL C

Uncropped western blot images for pS6$^{Ser235/236}$

pS6 $^{Ser235/236}$

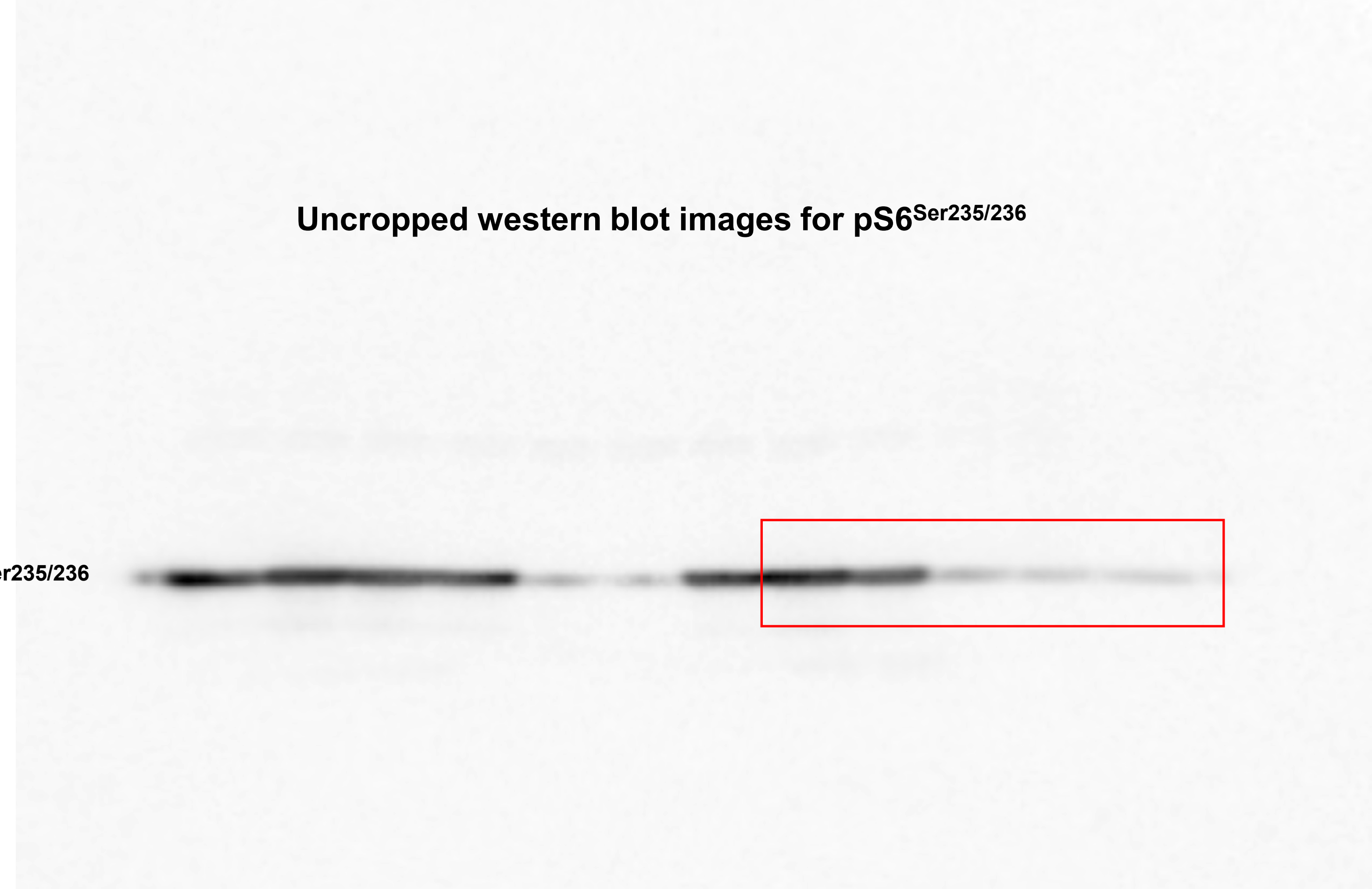

FIGURE 4 PANEL C

Uncropped western blot images for pERKThr202/204

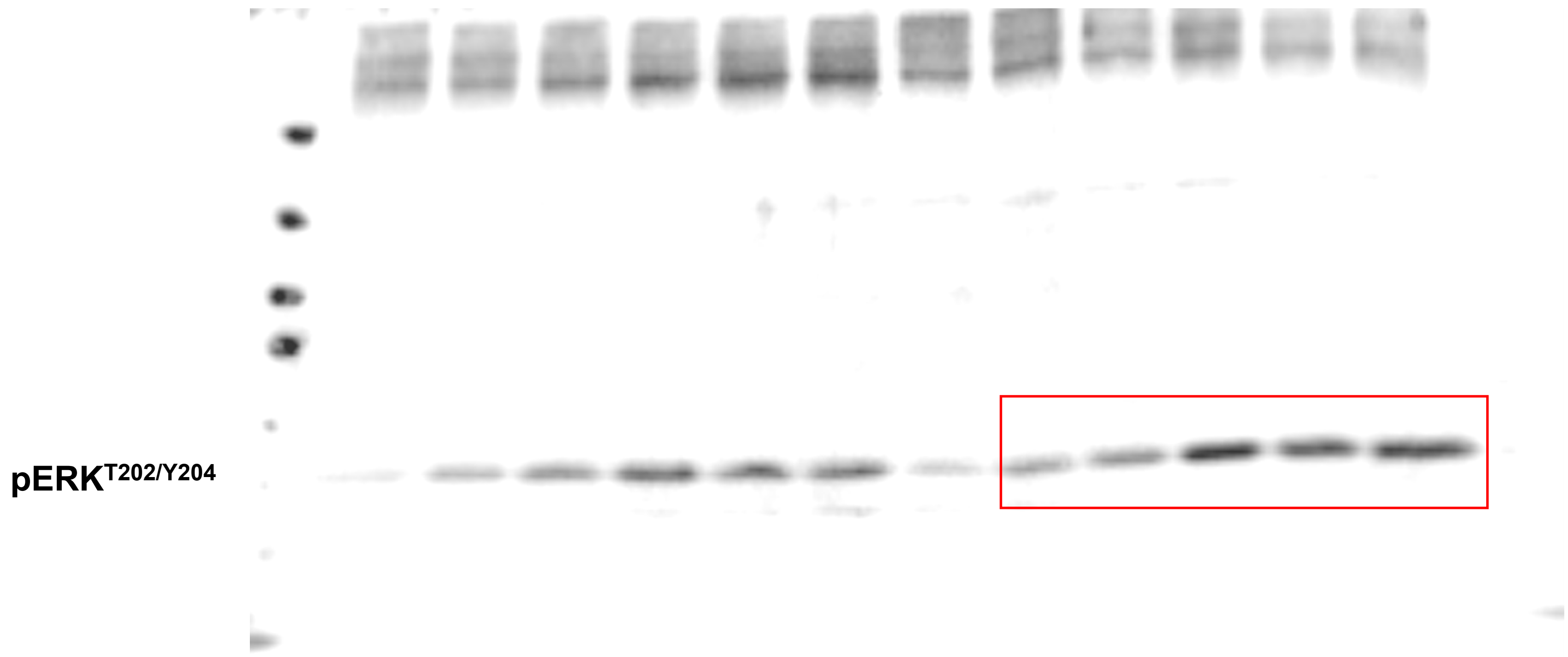

FIGURE 4 PANEL C

Uncropped western blot images for Total ERK

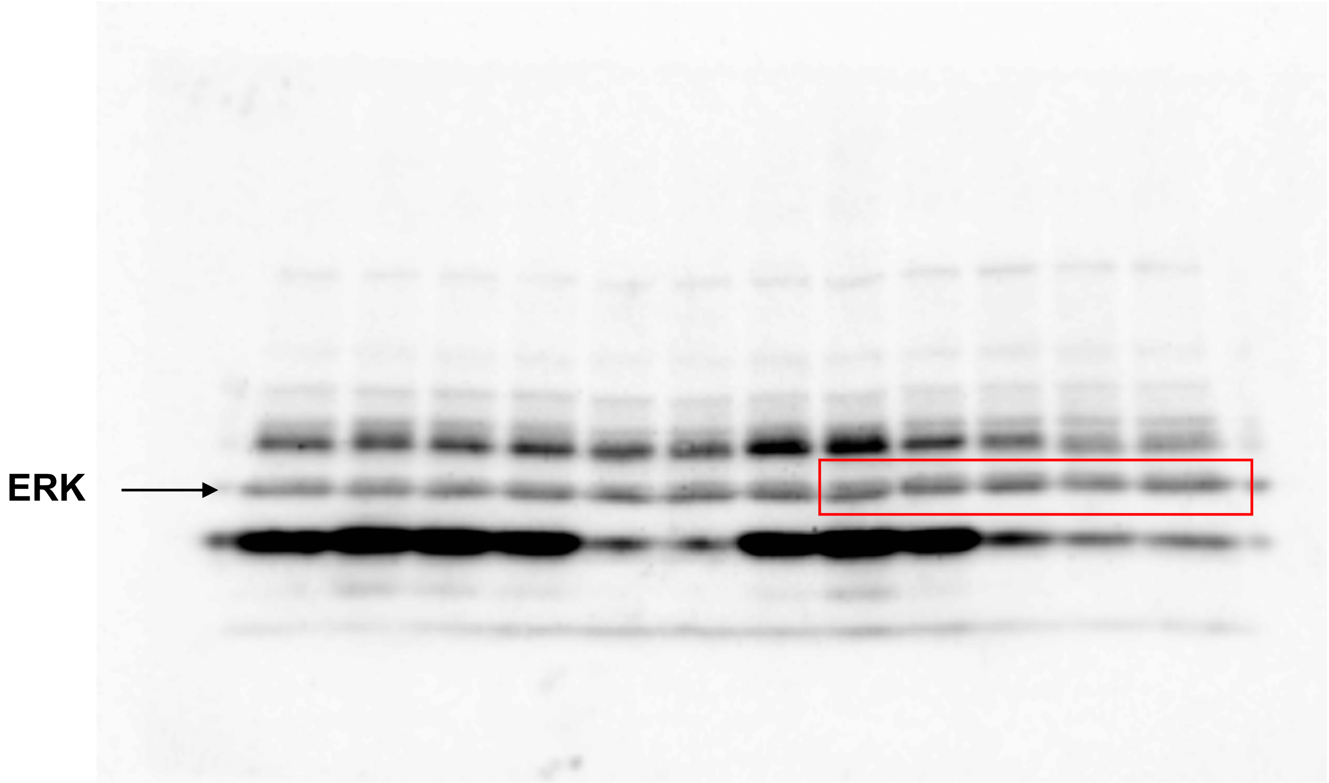

FIGURE 4 PANEL D

Uncropped western blot images for pERK$^{T202/Y204}$

pERK$^{T202/Y204}$

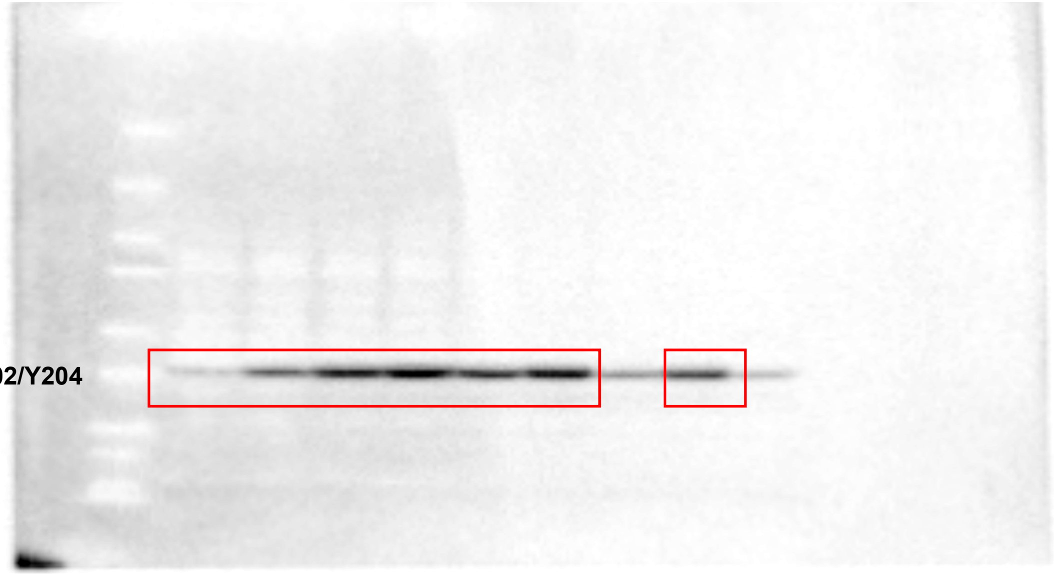

FIGURE 4 PANEL D

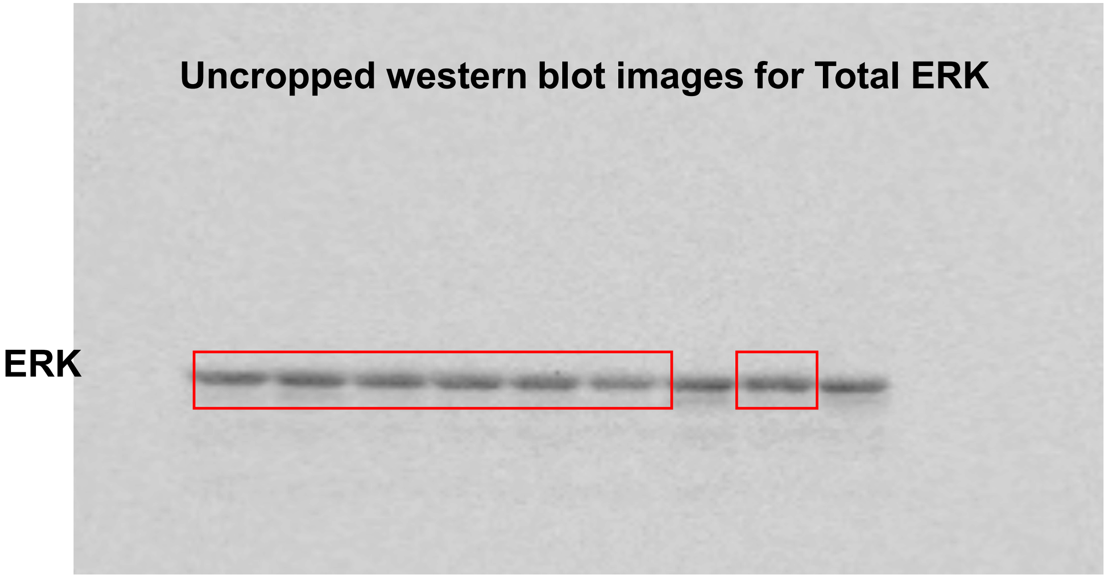

FIGURE 4 PANEL D

Uncropped western blot images for pAKT Ser473

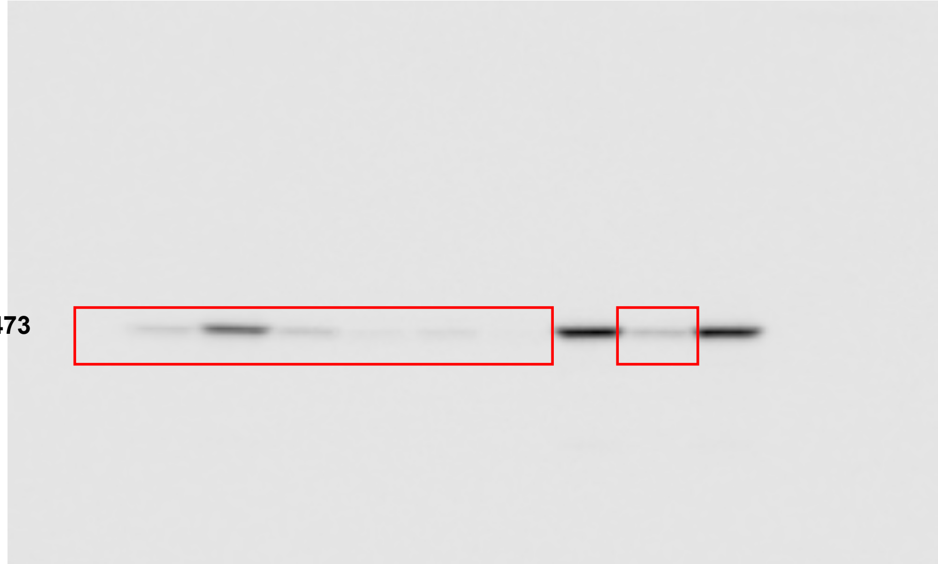

FIGURE 4 PANEL D

Uncropped western blot images for pAKT^{Thr308}

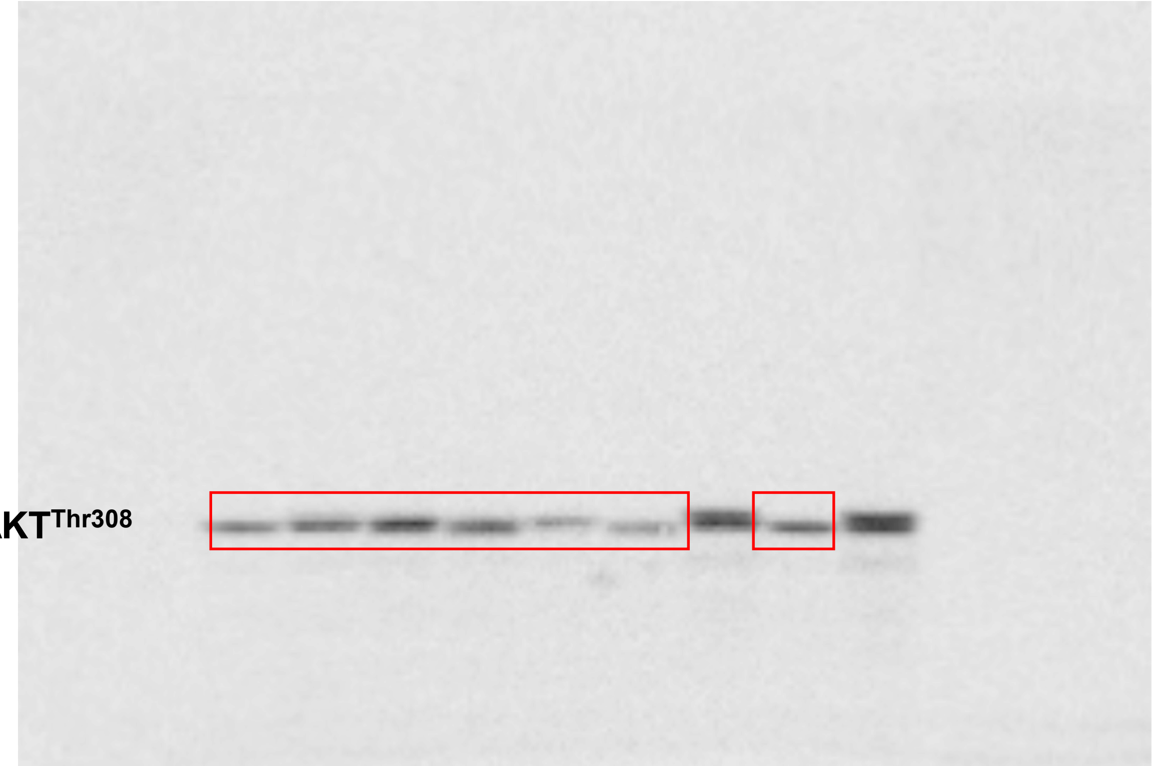

FIGURE 5

Uncropped western blot images for pAKT^{Ser473}

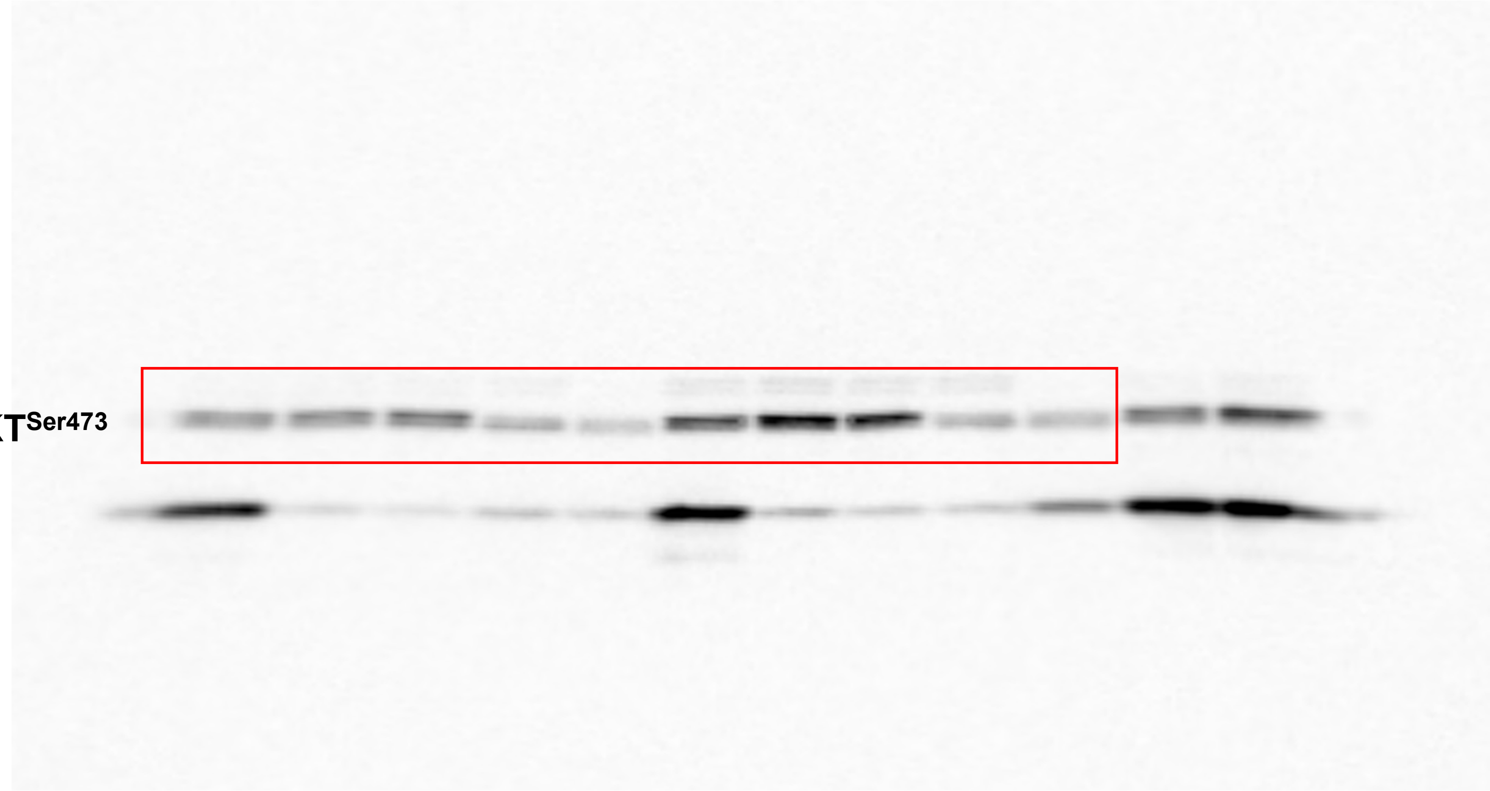

FIGURE 5

Uncropped western blot images for pS6$^{Ser235/236}$

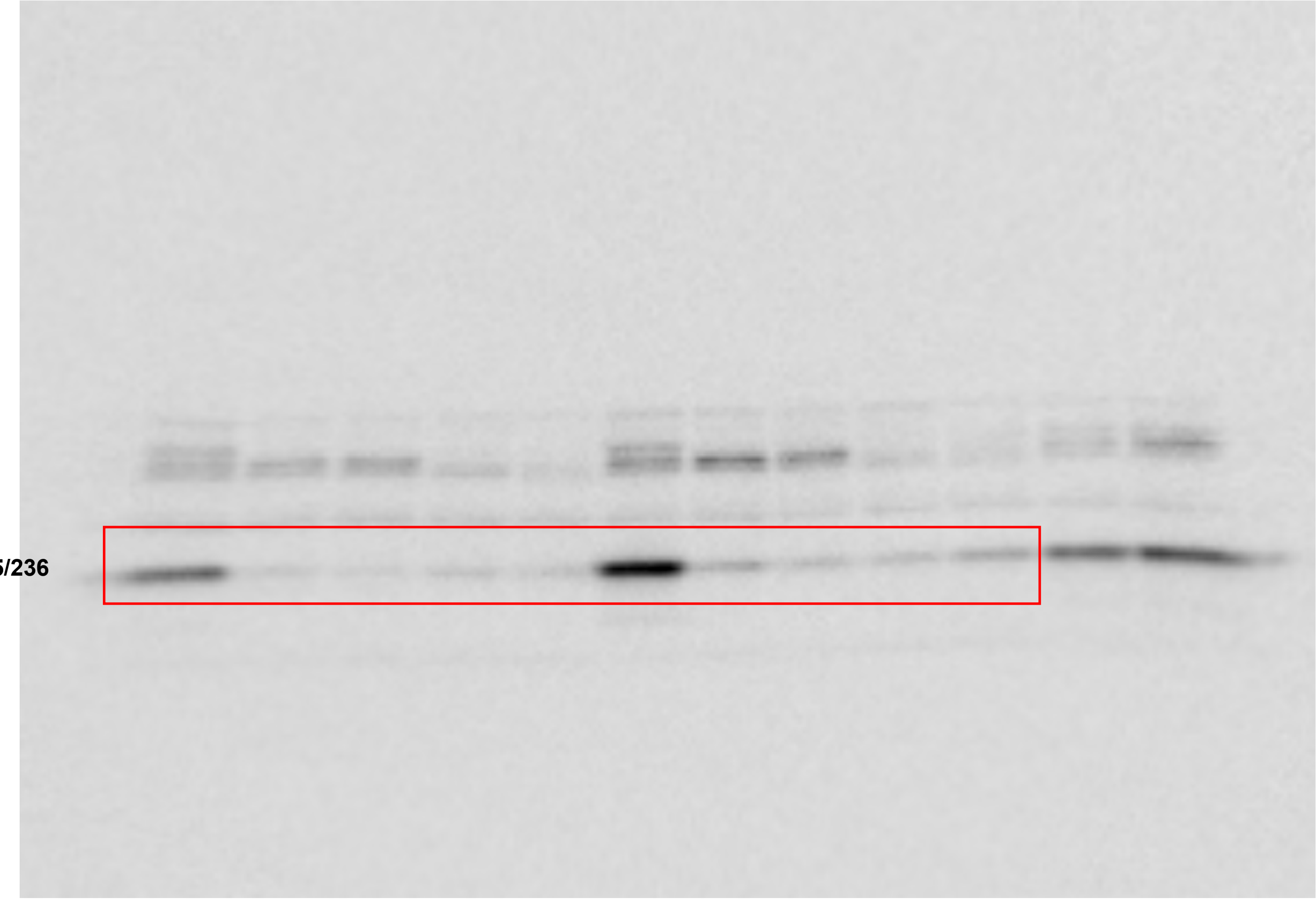

pS6$^{Ser235/236}$

FIGURE 5

Uncropped western blot images for Total S6

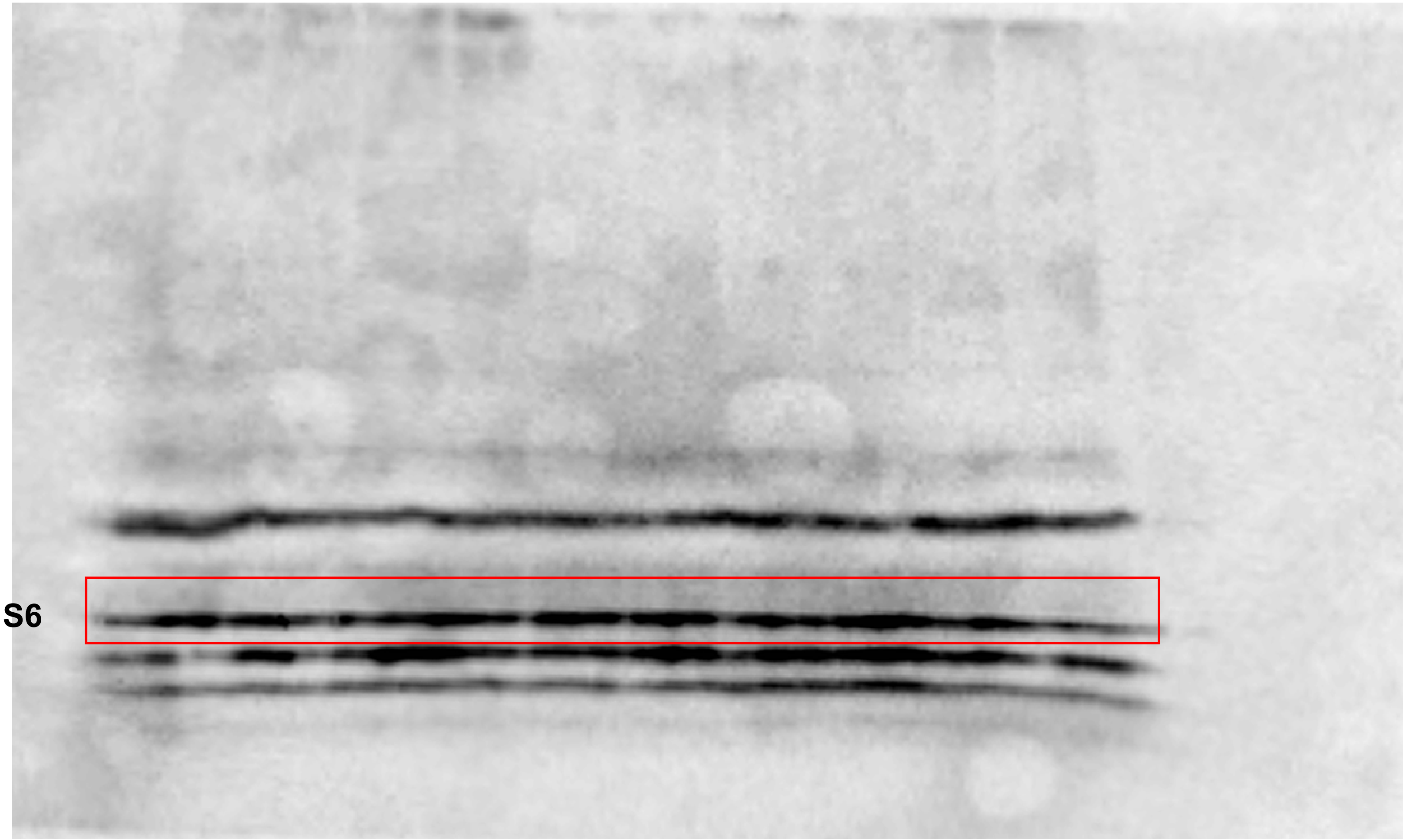

FIGURE 5

Uncropped western blot images for pERKT202/Y204

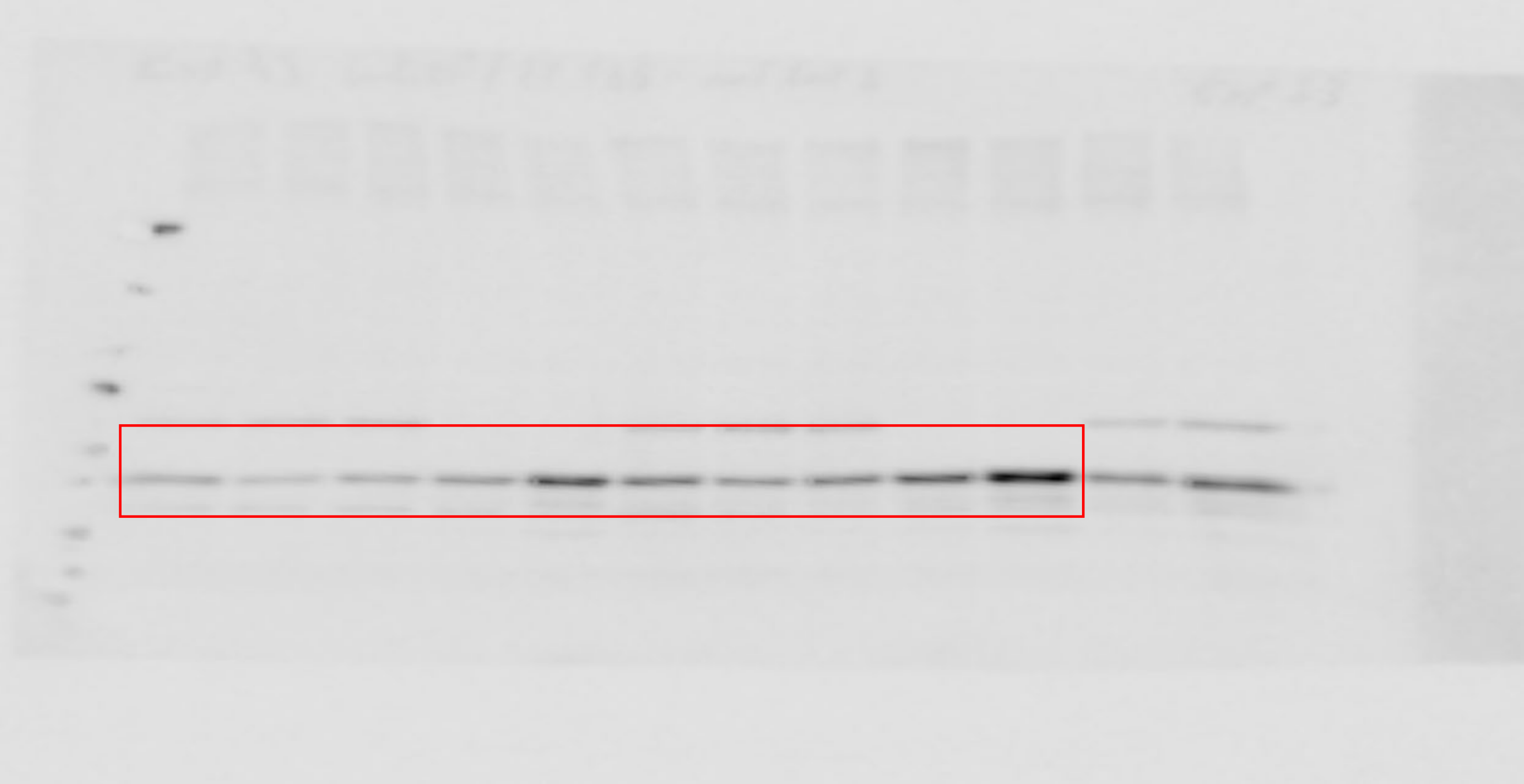

FIGURE 5

Uncropped western blot images for ERK

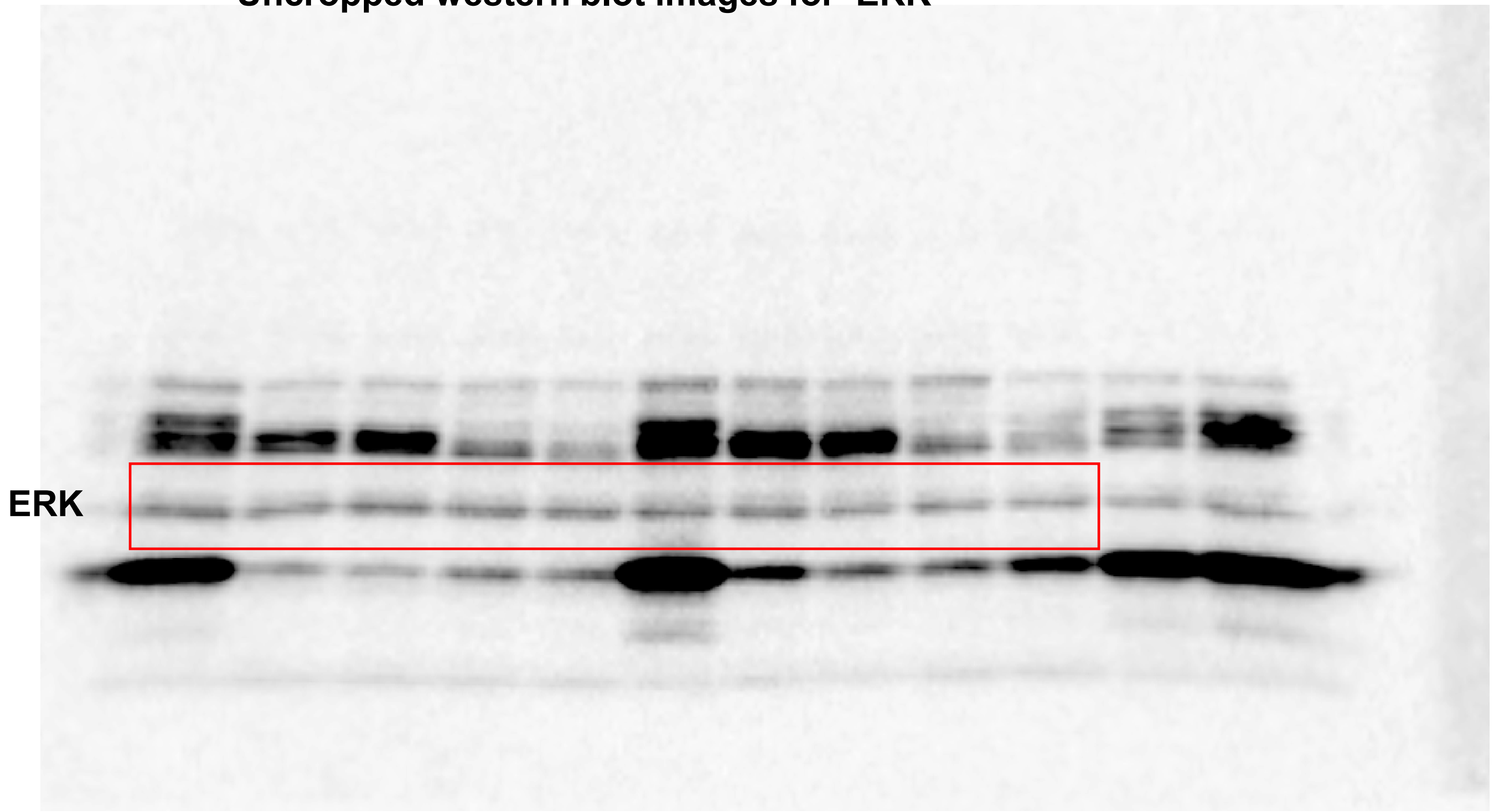

FIGURE 5

Uncropped western blot images for GAPDH

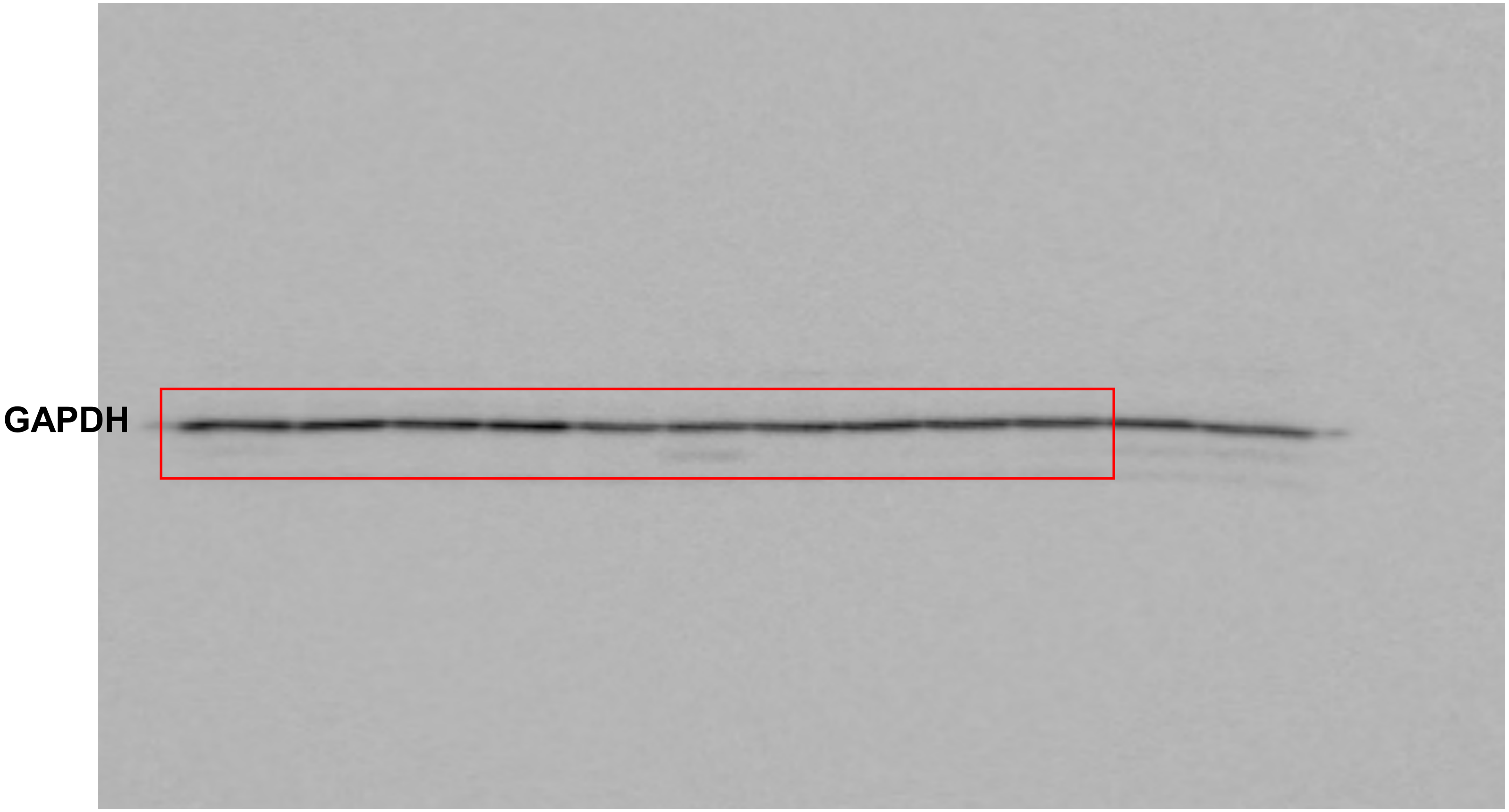

FIGURE 6 PANEL A

Uncropped western blot images for pS6K^{Thr389}

pS6K^{Thr389}

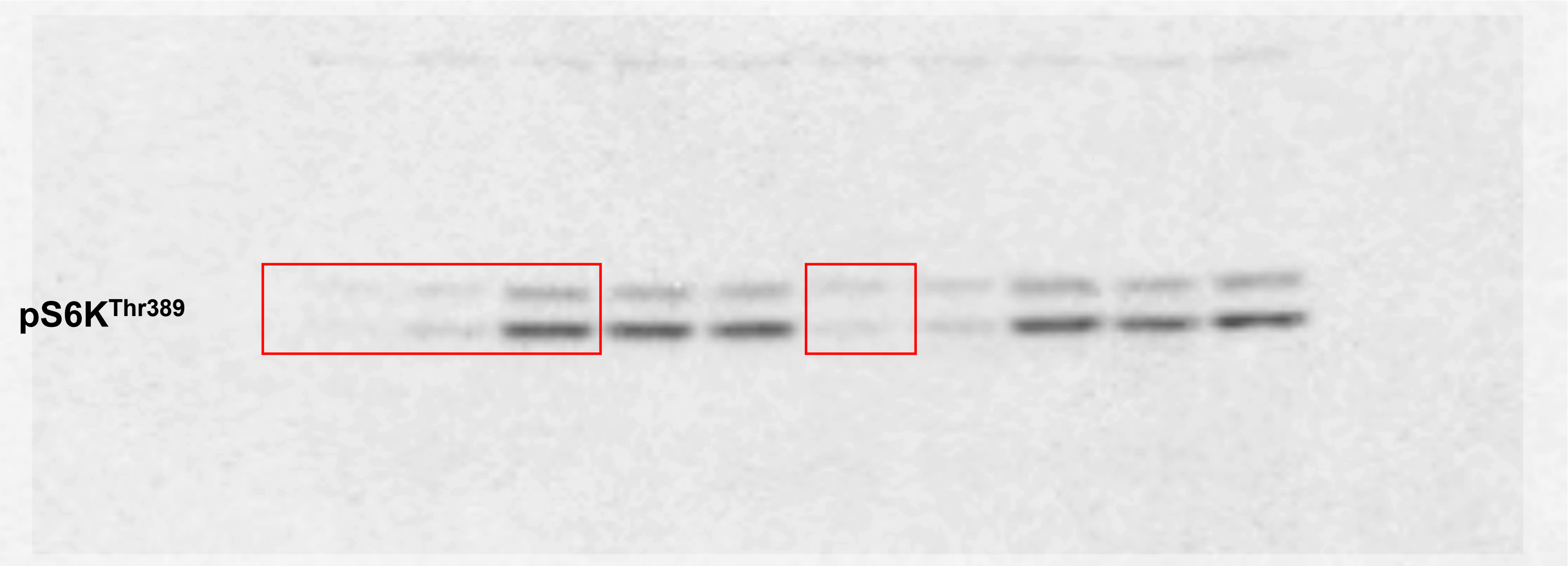

FIGURE 6 PANEL A

Uncropped western blot images for pS6$^{Ser235/236}$

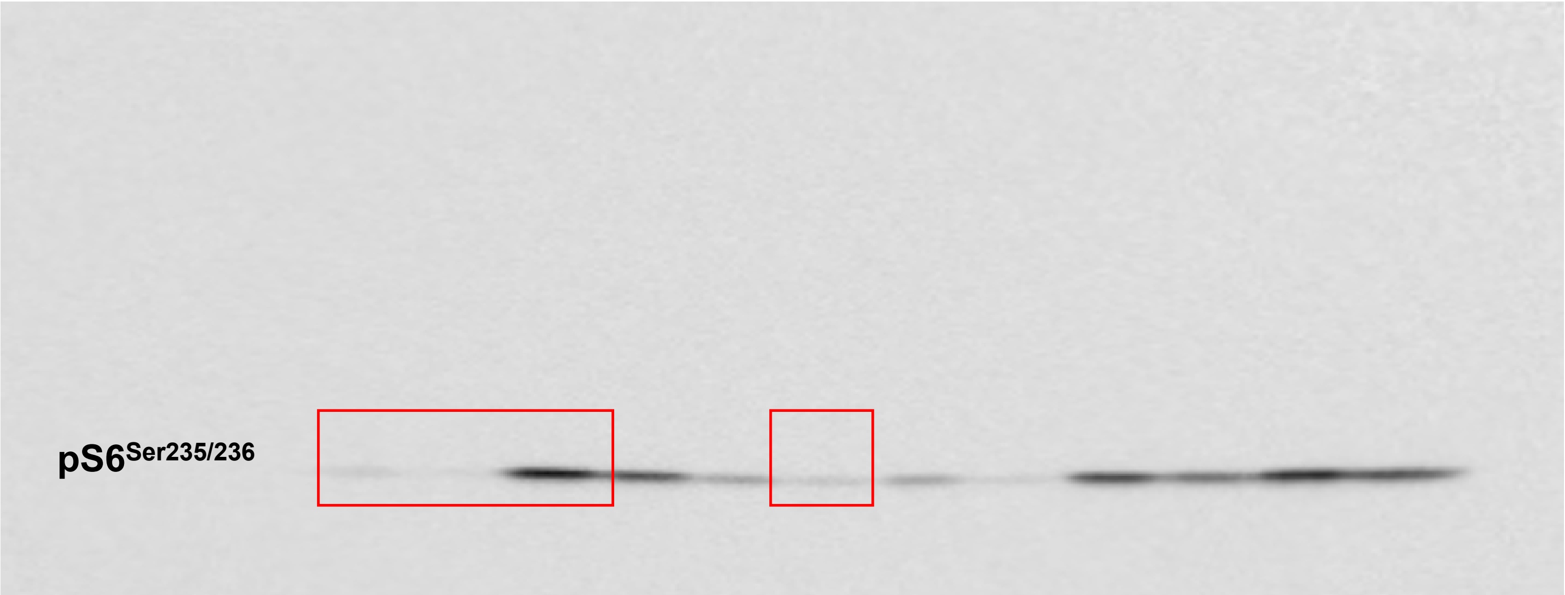

FIGURE 6 PANEL A

Uncropped western blot images for pERK$^{T202/Y204}$

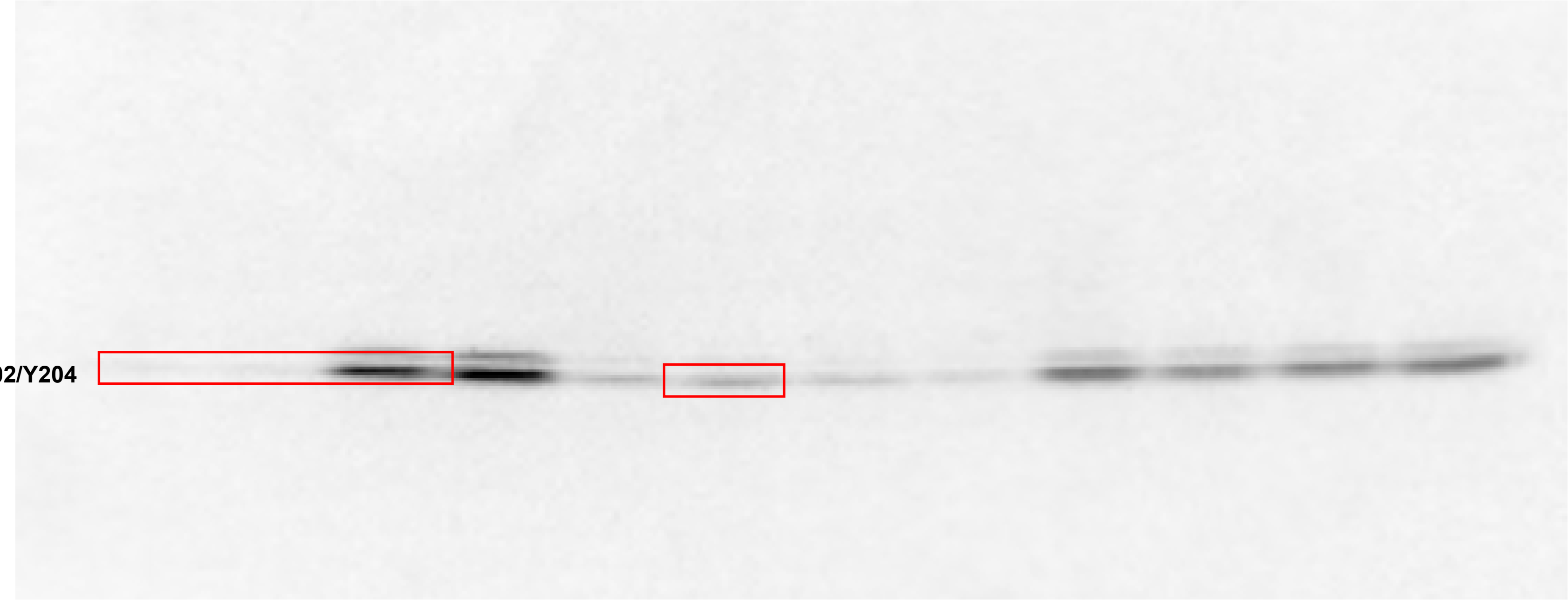

FIGURE 6 PANEL A

Uncropped western blot image for pACCSer79

pACC^{Ser79}

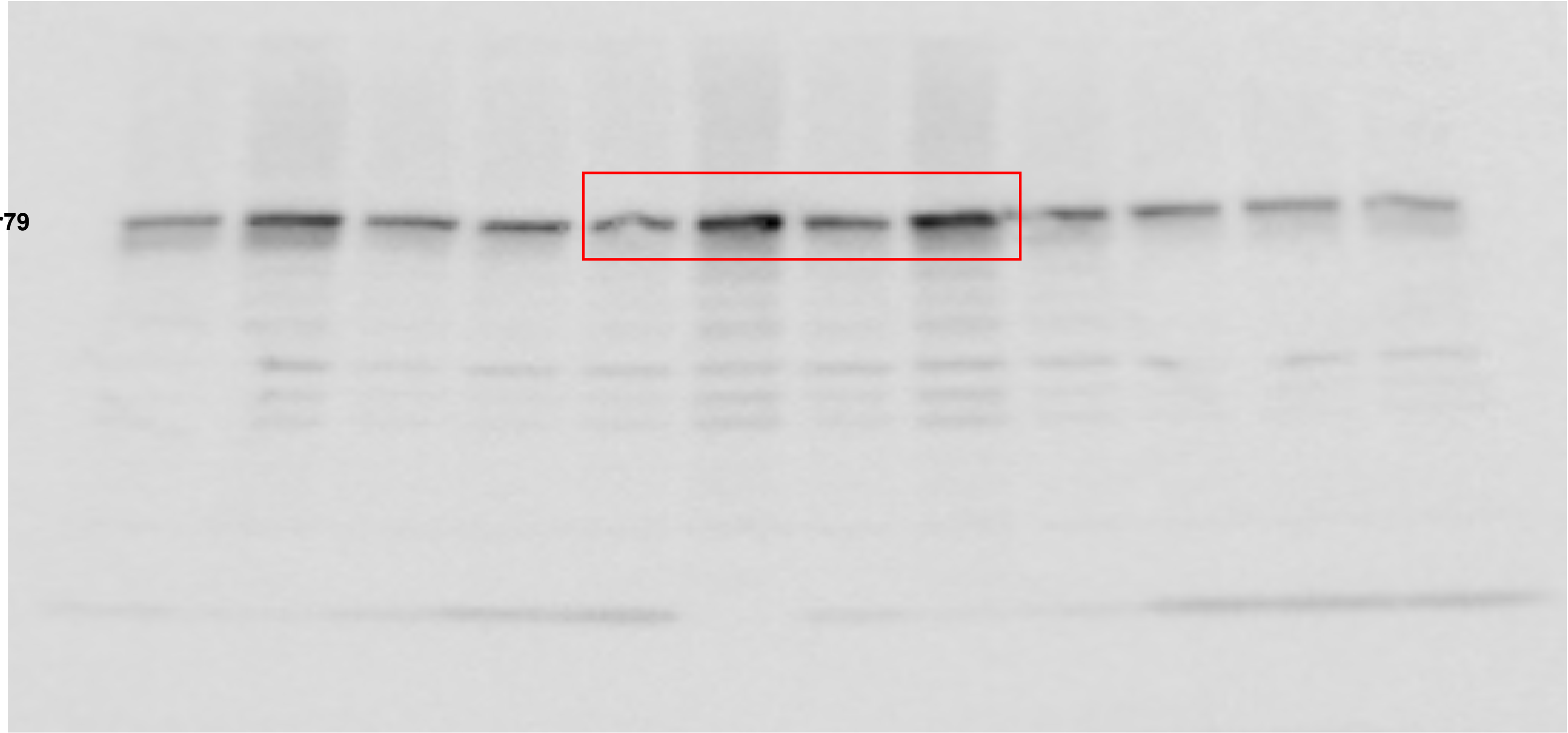

FIGURE 6 PANEL A

Uncropped western blot image for GAPDH

GAPDH

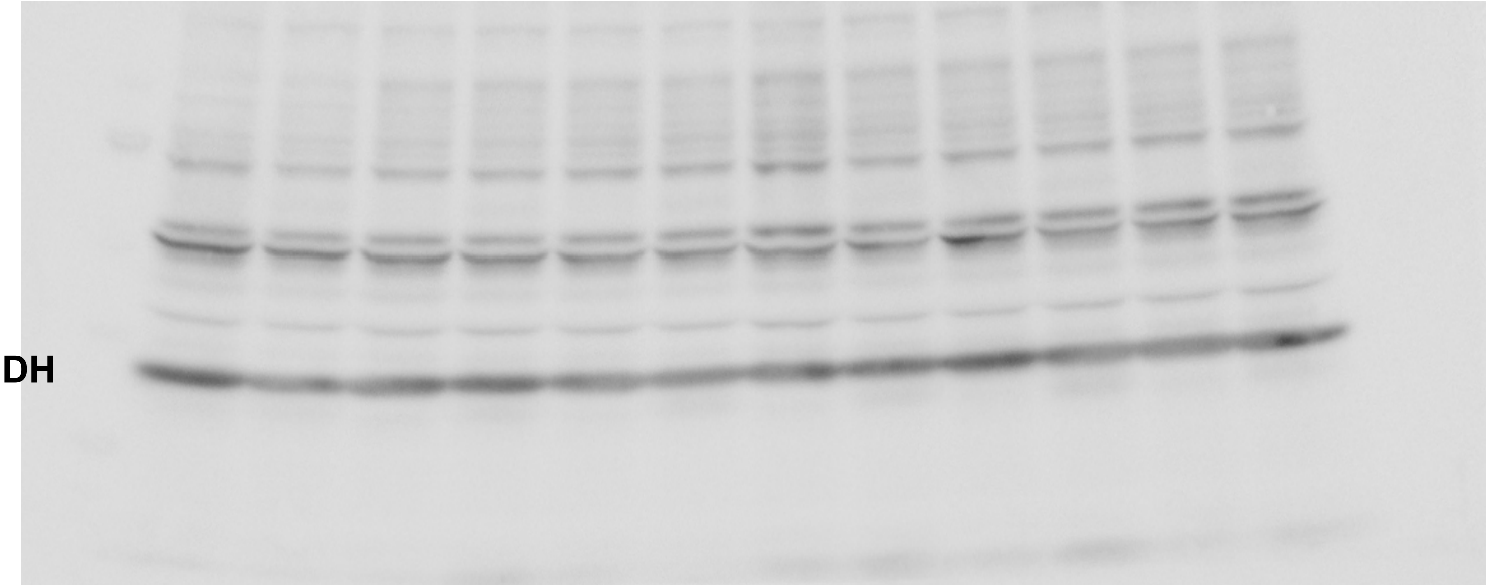

FIGURE 6 PANEL B

Uncropped western blot images for pS6K^{Thr389}

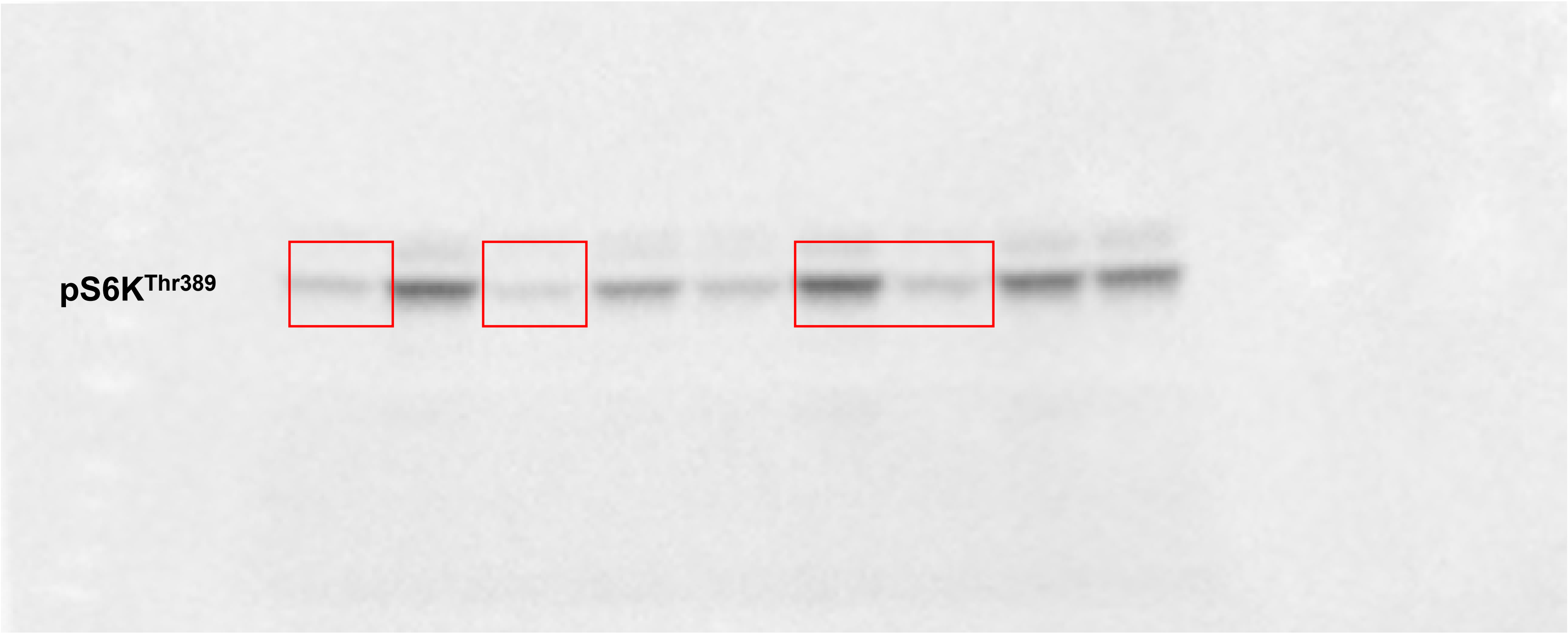

FIGURE 6 PANEL B

Uncropped western blot images for pS6$^{Ser235/236}$

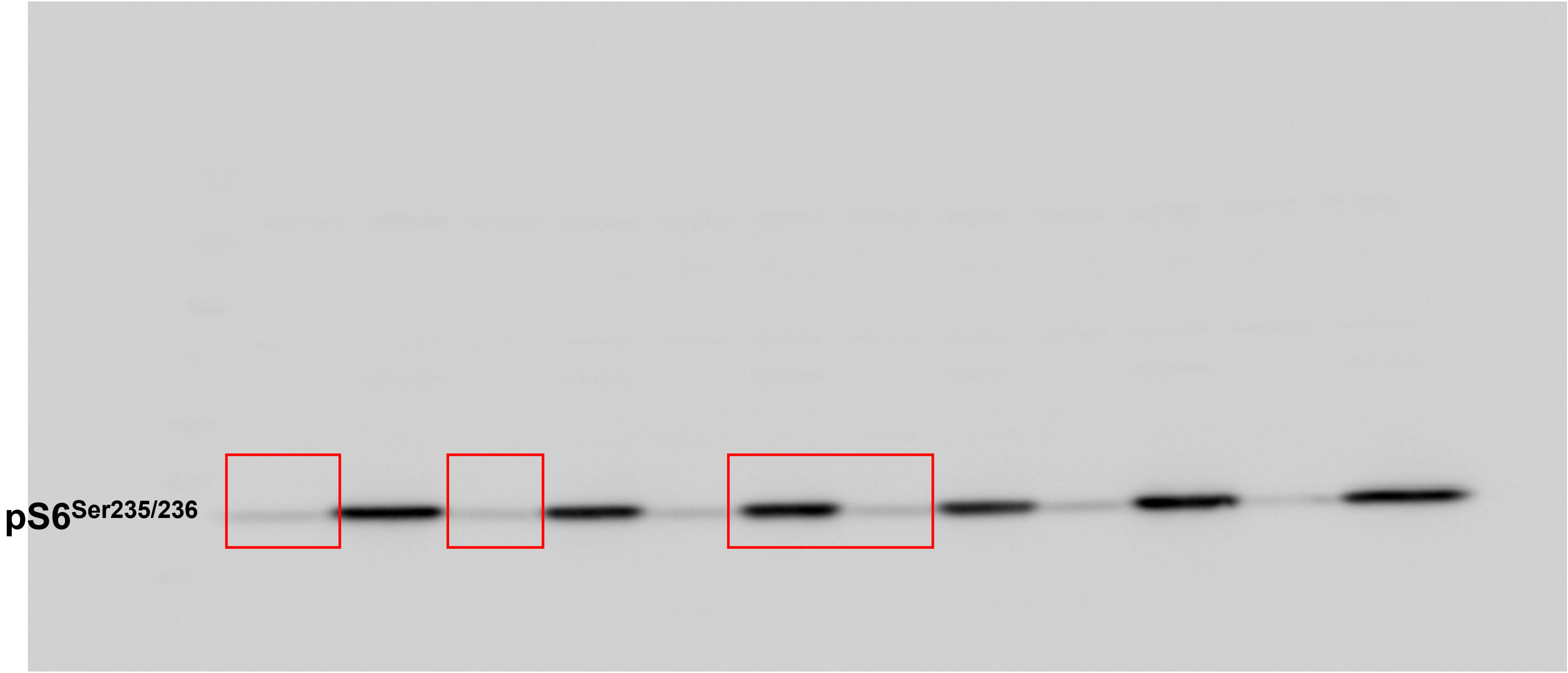

FIGURE 6 PANEL B

Uncropped western blot images for S6

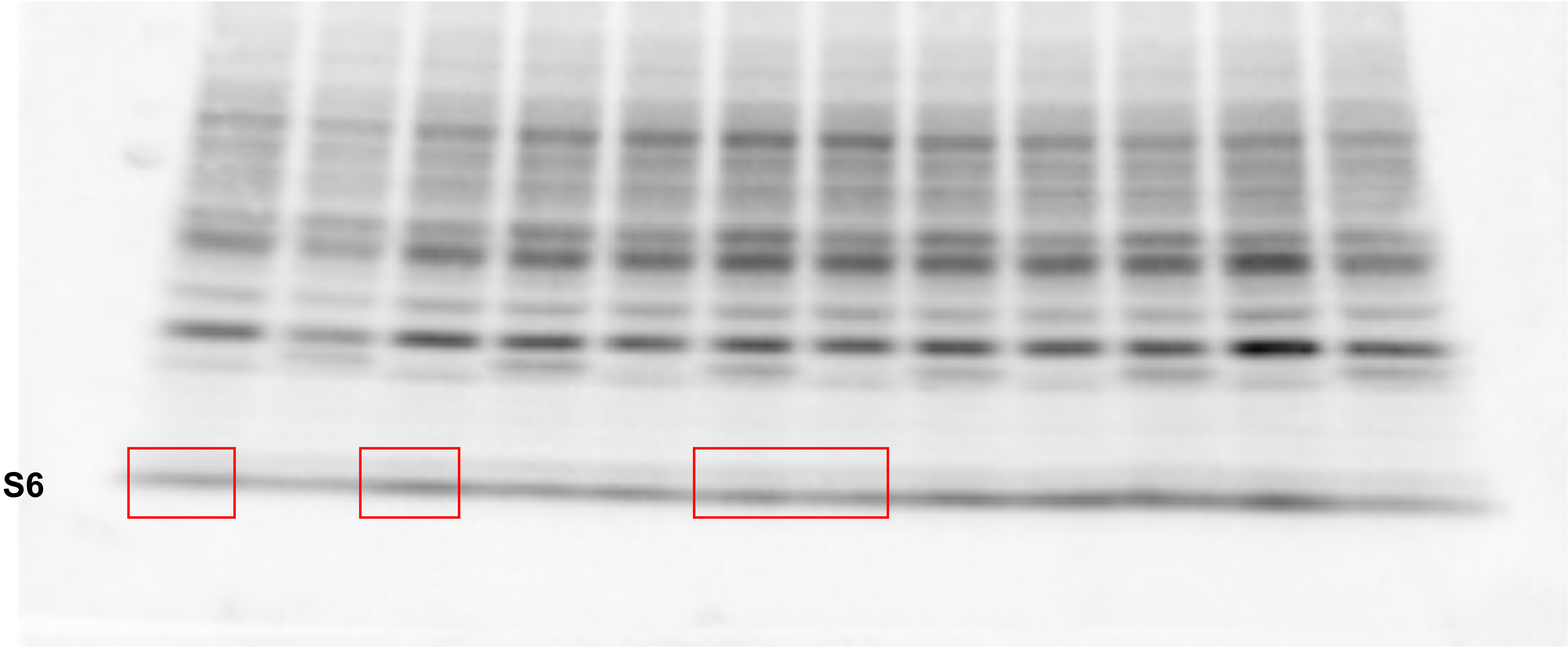

FIGURE 6 PANEL B

Uncropped western blot images for $pAKT^{Ser473}$

$pAKT^{Ser473}$

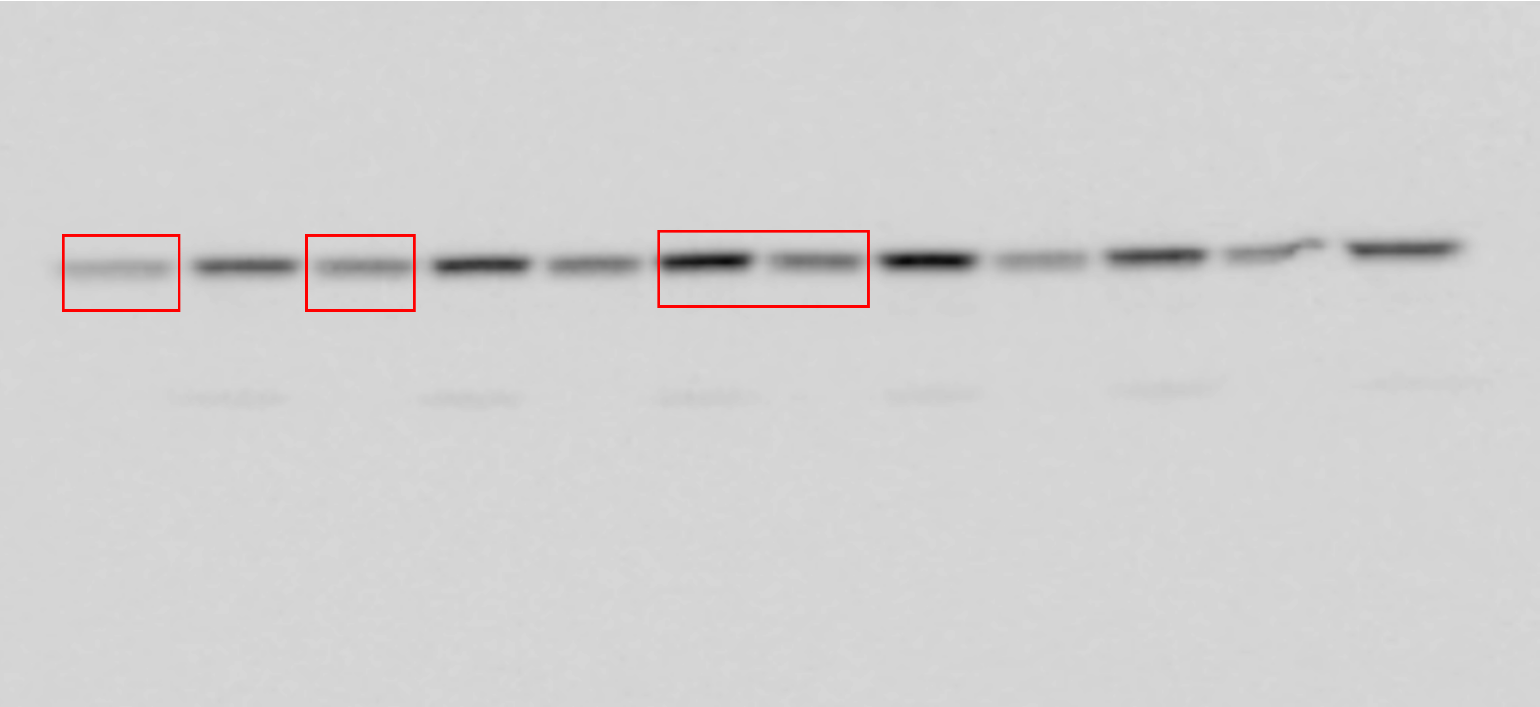

FIGURE 6 PANEL B

Uncropped western blot images for pERK$^{T202/Y204}$

pERK$^{T202/Y204}$

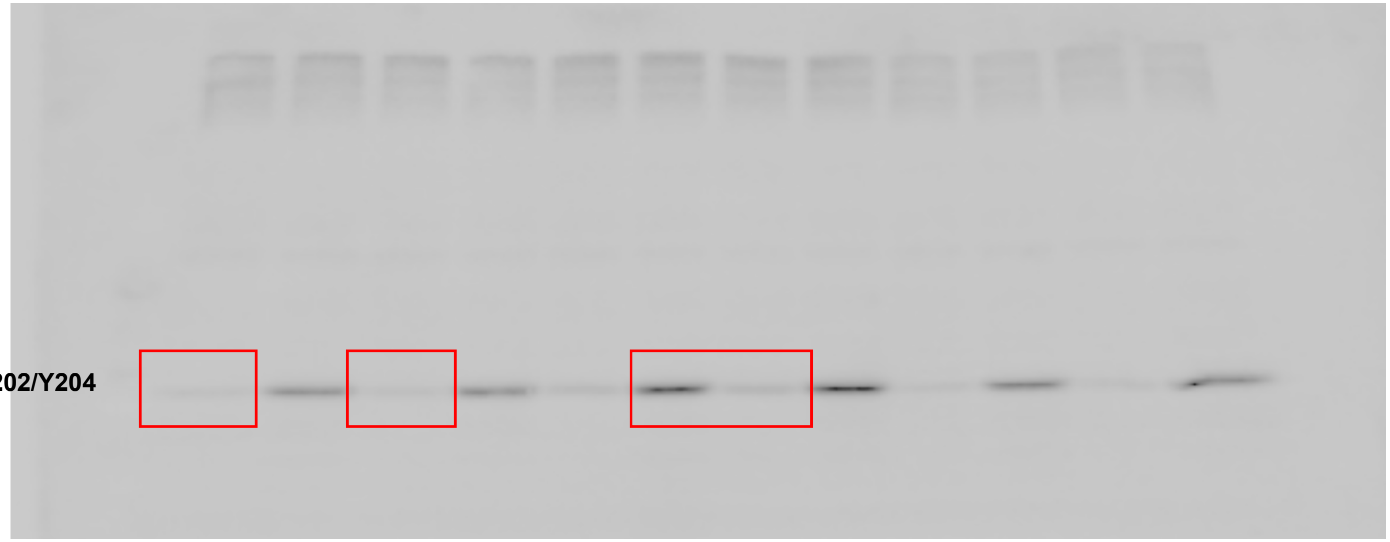

FIGURE 6 PANEL B

Uncropped western blot images for ERK

ERK

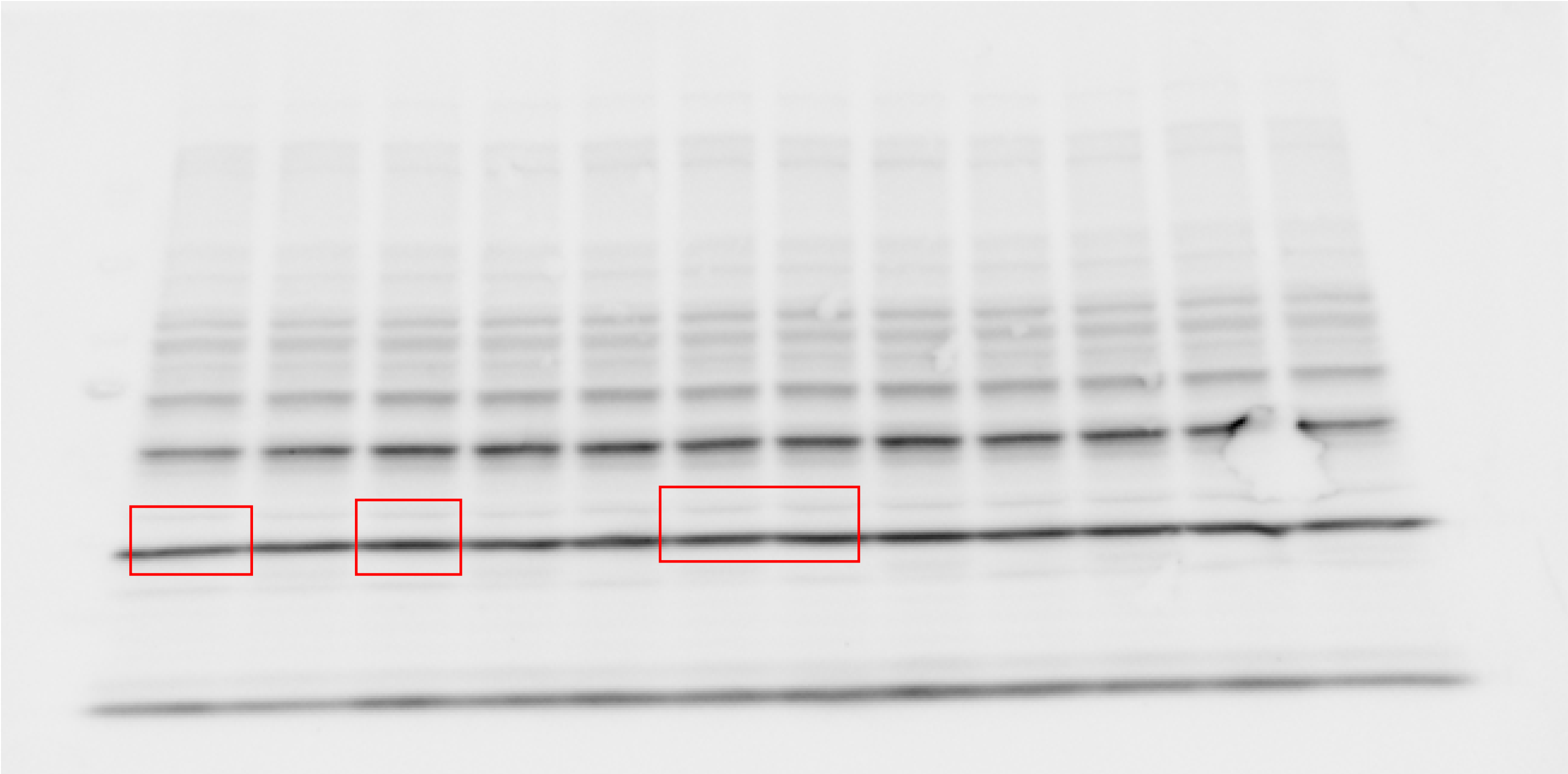

FIGURE 6 PANEL C

Uncropped western blot image for pS6KThr389

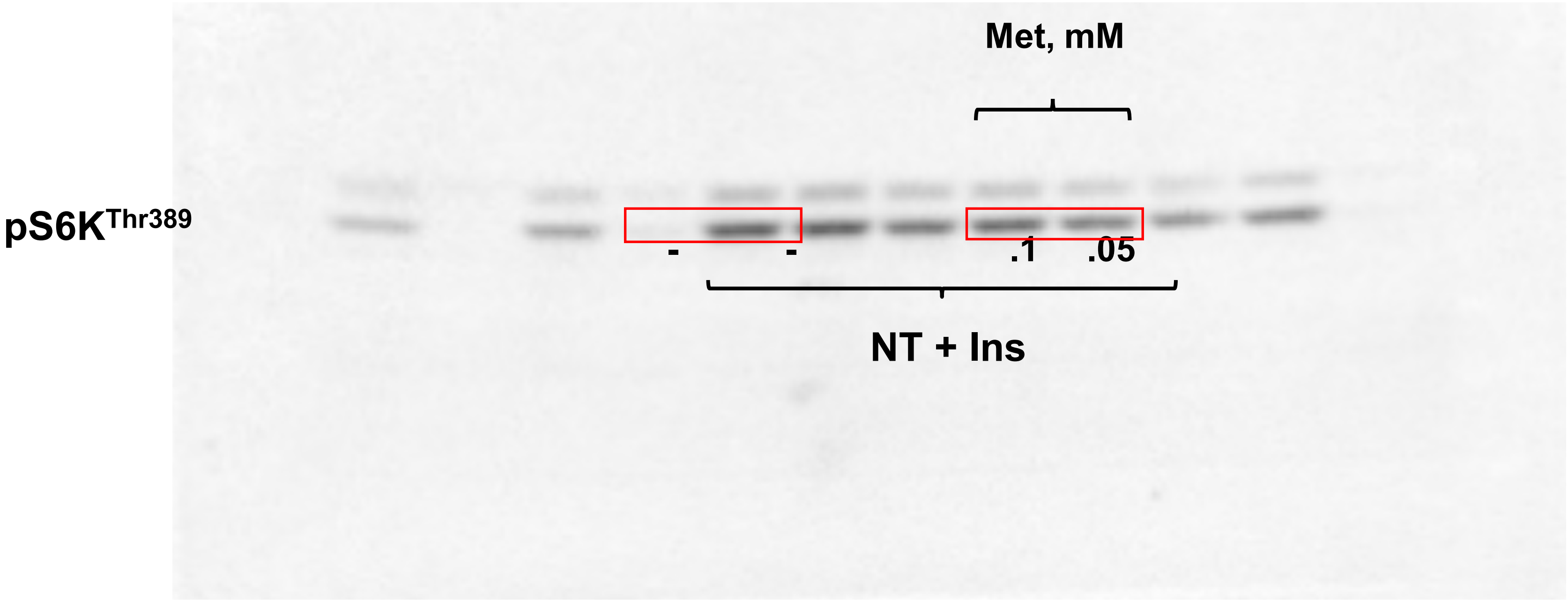

FIGURE 6 PANEL C

Uncropped western blot image for GAPDH

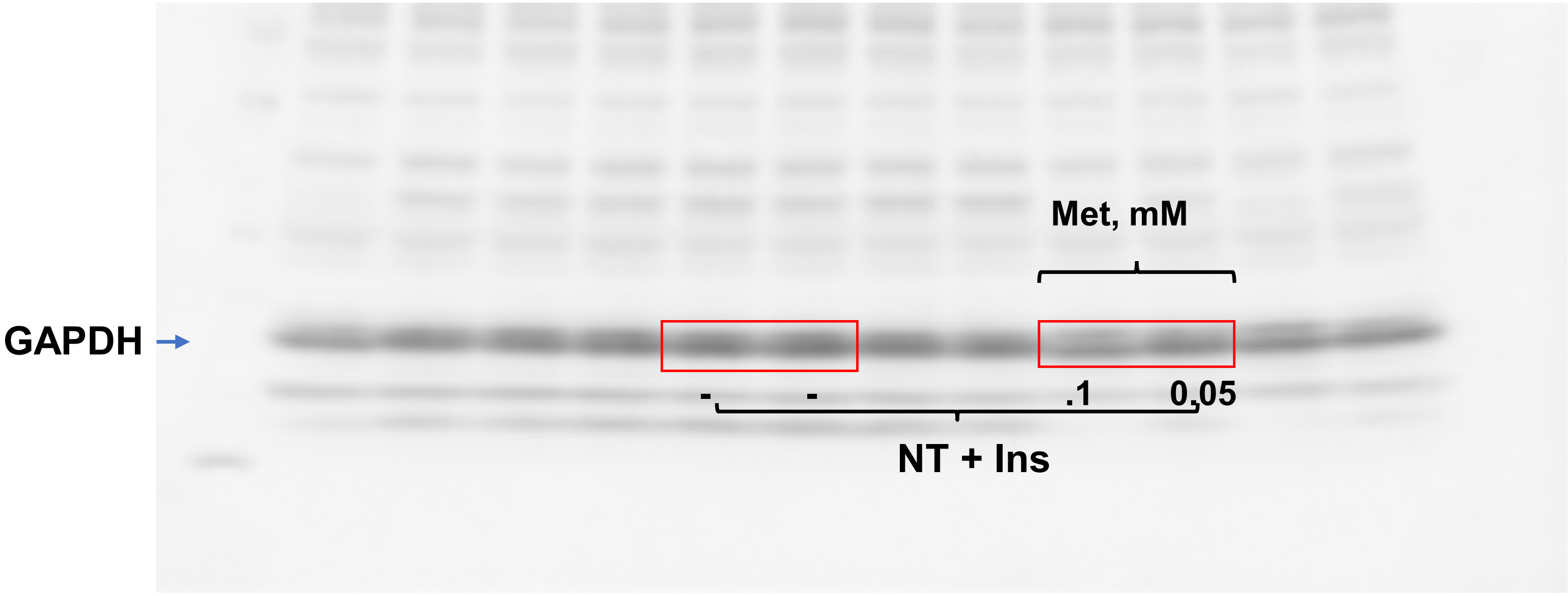

FIGURE 6 PANEL C

Uncropped western blot image for pERKT202/Y204

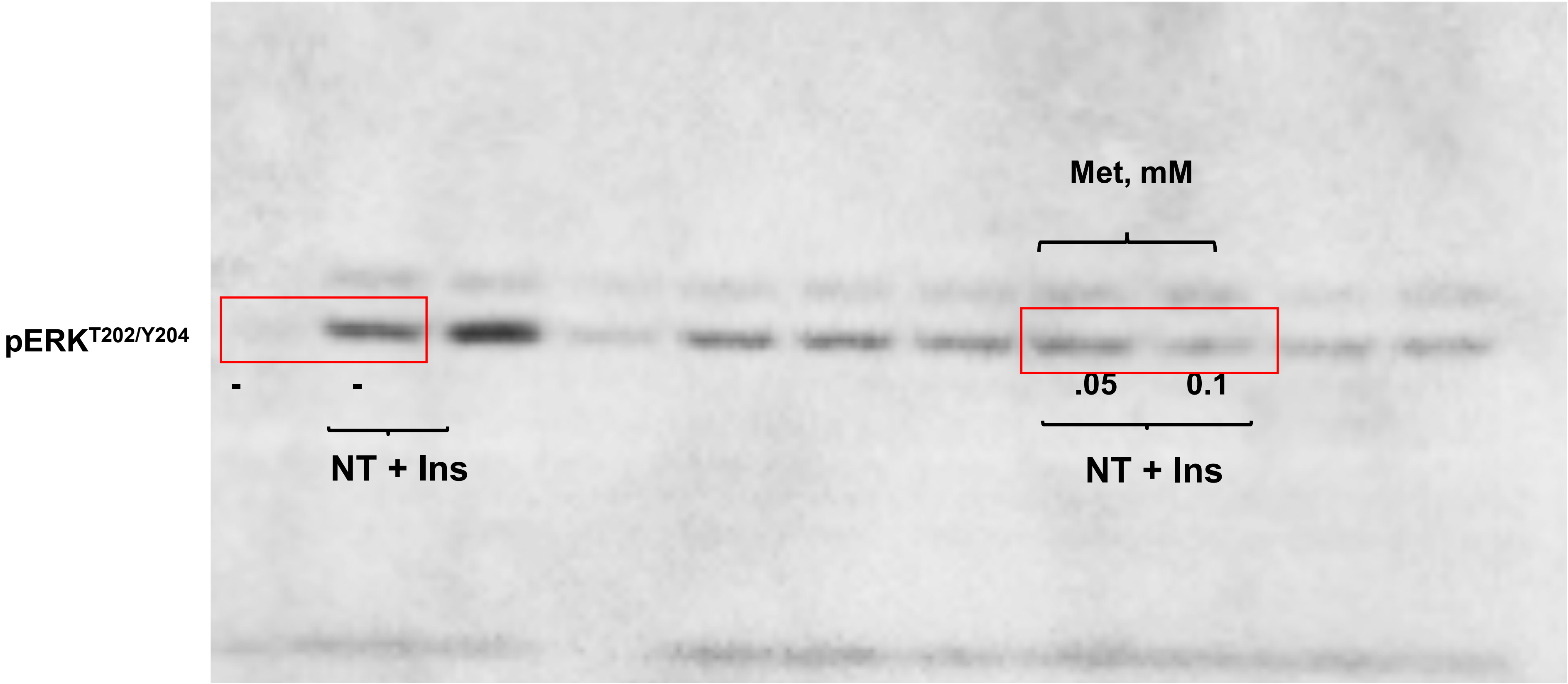

FIGURE 6 PANEL C

Uncropped western blot image for pACCSer79

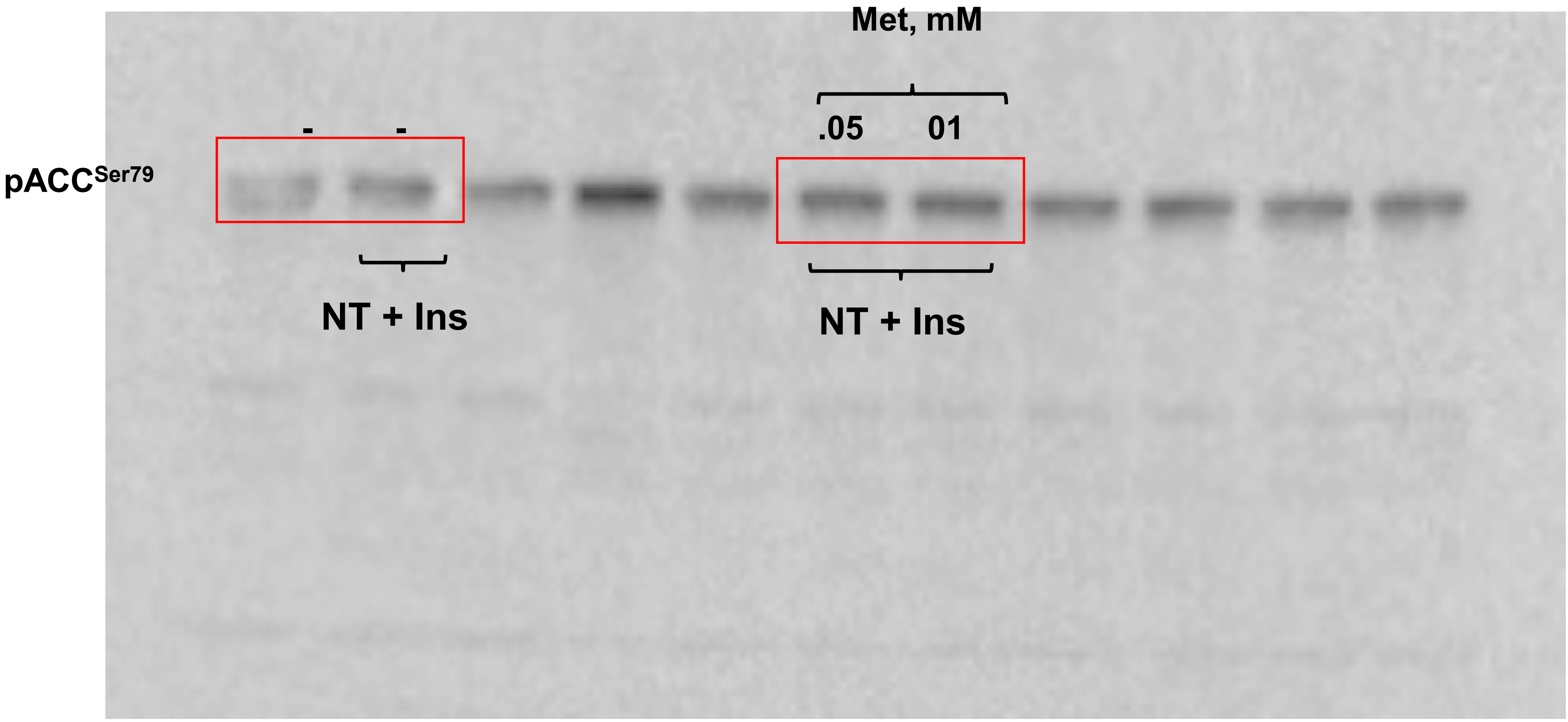

Supplement: S1 File — (PDF) [file pone.0292422.s001.pdf]
